# A Concise Total Synthesis of Tryprostatins A and B and Epimers

**DOI:** 10.1021/acsomega.5c07992

**Published:** 2025-10-08

**Authors:** Sophia Seideman, Khorshada Jahan, Frank Holger Foersterling, M. Mahmun Hossain

**Affiliations:** Department of Chemistry and Biochemistry, 14751 University of Wisconsin-Milwaukee, 2000 E Kenwood Blvd., Milwaukee, Wisconsin 53211-3029, United States

## Abstract

A concise four-step total synthesis of tryprostatins A and B has been developed, starting from commercially available gramine. The key step features a coupling between a *C*2*,N′*-diprenylated gramine salt and a diketopiperazine mediated by quinine as a bifunctional base. The synthesis afforded tryprostatins A and B along with their respective epimers in 1:1 ratios as determined by isolation and separation via column chromatography. The overall combined yields were 22% for tryprostatin A and its epimer and 44% for tryprostatin B and its epimer.

## Graphical abstract

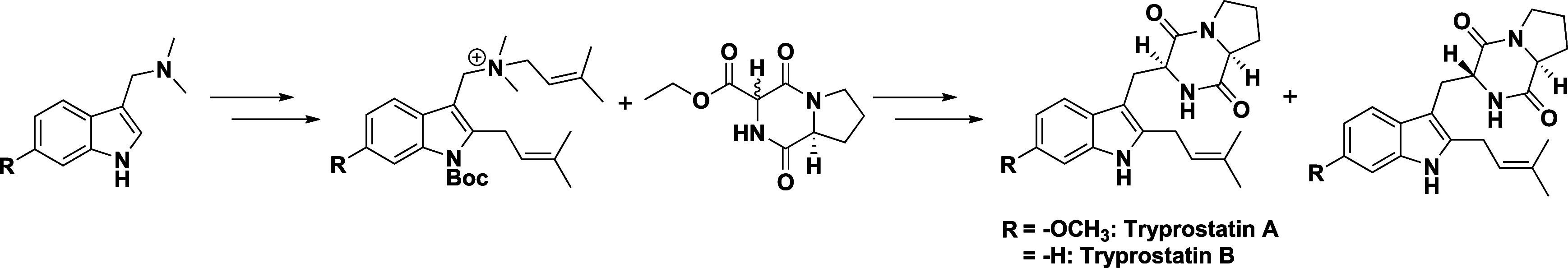

## Introduction

Tryprostatins A (**1**) and B (**2**) (TPS A and TPS B) are *C*2-prenylated Trp-Pro diketopiperazine alkaloids. First isolated in 1995 by Osada and colleagues from *Aspergillus fumigatus* BM939, these natural products are notable for their ability to inhibit the cell cycle of mouse tsFT210 cells, with minimum inhibitory concentrations (MICs) of 16.4 μM for TPS A and 4.4 μM for TPS B.^1^ TPS A primarily induces G2/M phase cell cycle arrest by inhibiting tubulin polymerization.^1c^ and has shown potential in reversing drug resistance by targeting the breast cancer resistance protein (BCRP/ABCG2).^2^ While TPS B exhibits greater potency than TPS A, its cytotoxicity is not restricted to the M phase. Due to their limited natural availability, potent biological activity, and structurally intriguing frameworks, TPS A and B have attracted considerable interest from the synthetic chemistry community. As a result, several total syntheses have been reported, typically requiring between 6 and 14 steps.^3^ Notable examples include an 8-step synthesis of TPS B by Depew et al., which delivered a 35% overall yield,^4^ and a 6-step synthesis reported by the Cook group, achieving 56% and 54% overall yields for TPS A and B, respectively.^3^ ^k^ More recently, Yin and co-workers completed a 12-step linear synthesis of TPS A from alanine, yielding 25% overall using a Pd/norbornene-promoted C–H activation strategy.^3e^

In 2019, our research group reported an efficient asymmetric total synthesis of TPS B in just six steps, achieving an overall yield of 35%. This approach utilized readily available and cost-effective reagents, as outlined in Scheme 1.^5^ More recently, Hu and co-workers developed a late-stage prenylation strategy for dipeptides using *tert*-prenyl alcohol in the presence of AlCl_3_.^6^ They described both 4-step and 3-step syntheses of TPS B. In the 4-step route, Brevianamide F was first constructed from protected amino acids, followed by prenylation to afford TPS B in 22% overall yield. In the shorter 3-step sequence, a protected Pro-Trp dipeptide was prenylated and subsequently cyclized, resulting in a 38% overall yield.

**Scheme 1:**
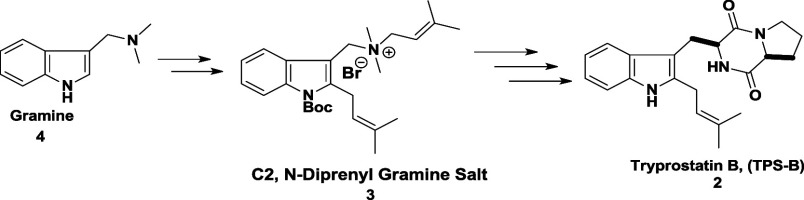
Previous Total Synthesis of Tryprostatin B by Hossain et al.

In pursuit of a synthetic route amenable to the rapid generation of Tryprostatins and their analogs for structure–activity relationship (SAR) studies, we sought to refine our previously reported 2019 strategy. Drawing inspiration from the work of Dubey and Olenyuk, we envisioned a concise approach to access both TPS A and TPS B via conjugate addition of a diketopiperazine to a *C*2,*N*′-diprenylated gramine salt (**3**). Two promising synthetic pathways were designed and are elaborated in this study. Upon successful development, these approaches would enable a modular “mix-and-match” strategy, combining *C*2-alkylated gramine derivatives with diketopiperazines constructed from diverse amino acid residues. This strategy would facilitate rapid access to a library of Tryprostatin analogs. The preparation of *C*2-alkylated gramine intermediates has been previously explored by our group.^7^ Thus, compared to other concise syntheses, our approach offers a more general platform for analog development. This paper outlines the investigation and optimization of these synthetic routes, aimed at enabling the efficient synthesis of novel TPS analogs for advanced biological evaluation.

## Results and Discussion

Based on our retrosynthetic analysis, we identified two potential synthetic routes for the construction of tryprostatins from gramines. A summary of these pathways is presented in Scheme 2. In Route 1, we envisioned a conjugate addition between the *C*2,*N*′-diprenylated gramine salt **3** and *N*-Boc-protected diketopiperazine **5**, mediated by a strong base. The base would generate an enolate from compound **5**, enabling nucleophilic addition to the electrophilic gramine salt. The *N*-Boc protecting group at the 5-position of the diketopiperazine was expected to suppress undesired side reactions.

**Scheme 2:**
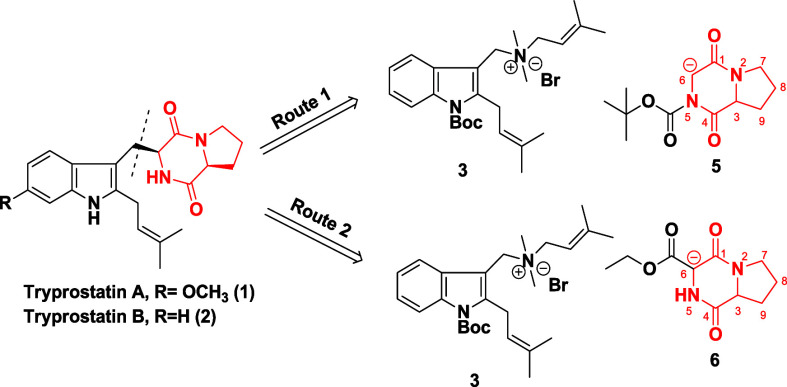
Retrosynthetic Plan

Although Route 1 requires the use of a strong base, our prior studies demonstrated that the Boc group is cleaved under the conditions of the phase-transfer-catalyzed conjugate addition. As such, this strategy enables a concise, three-step total synthesis of tryprostatins.^5^

In alternative Route 2, we planned to introduce the diketopiperazine moiety (highlighted in red) using a diketopiperazine derivative **6** bearing an electron-withdrawing ethyl ester at the *C*6 position. This substituent was expected to facilitate coupling with the *C*2,*N*′-diprenylated gramine salt **3** by enhancing enolate formation, thereby requiring a milder base than in Route 1. However, this route would also require an additional step to remove the ethyl ester via Krapcho decarboxylation.^3i^

Given the potential for a more streamlined three-step total synthesis, we initially pursued Route 1. This route began with the preparation of the diketopiperazine core **5**, accomplished via a modified version of a reported procedure^8^ (Scheme 3). Unprotected diketopiperazine **10** was synthesized by coupling Fmoc-Proline–OH with ethyl glycinate hydrochloride using PyBOP as the coupling agent. Subsequent Fmoc deprotection and cyclization were carried out in a 20% piperidine/DCM solution. Final Boc-protection was performed following the method of Ordóñez et al.,^8^ ^a^ with the key modification of using acetonitrile (ACN) in place of dichloromethane (DCM) as the solvent. The synthesis of *C*2,*N*′-diprenylated gramine salt **3**, the coupling partner for **5**, was carried out following our previously reported procedure.^5^

**Scheme 3:**
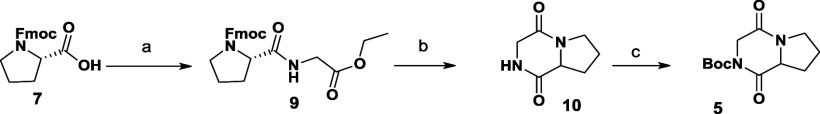
Synthesis of Compound **5** ^a^

a Conditions: a) Ethyl glycinate hydrochloride (**8**), PyBOP, DIPEA, DCM, 90% yield b) 20% piperidine/DCM, 80% yield c) (Boc)_2_O, DMAP, TEA, ACN, 85% yield.

Following the synthesis of coupling partners **3** and **5**, we explored direct alkylation using KOH as a strong base in the presence of a phase-transfer catalyst (PTC), aiming to minimize racemization under conditions previously developed by our group.^5^ The catalysts tested included *O*-allyl-*N*-(9-anthracenylmethyl)­cinchonidinium bromide (Cat A) and tetrabutylammonium iodide (TBAI, Cat B). Despite earlier success with similar conditions, neither catalyst promoted the desired coupling. Instead, diketopiperazine **5** decomposed under basic conditions, while compound **3** remained unreacted at room temperature. These findings are consistent with those of Farran et al., who reported that Boc-protected diketopiperazines undergo ring opening in the presence of strong nucleophiles, thus preventing direct alkylation.^9^

A literature search identified an alternative approach. Dubey and Olenyuk reported organocatalytic alkylation of gramine using a substituted diketopiperazine and quinine as a base.^10^ Adapting their method, we performed the reaction between **3** and **5** in refluxing acetonitrile (ACN) in the presence of quinine. However, compound **3** decomposed while **5** remained unreacted, indicating that quinine was too mild to generate an enolate from **5.**

We next tested NaH, as used by Kametani et al.,^11^ but no coupling was observed at 50 °C. Based on prior work,^10,12^ higher temperatures may be needed to form the reactive 3-methyleneindolenine intermediate. However, due to safety concerns associated with heating NaH in DMF, we replaced DMF with ACN, maintaining a polar aprotic environment while allowing a higher reaction temperature.^13^ This adjustment failed to yield the desired product. Variations in base and reactant ratios also had no effect. Lastly, we attempted the use of NaHMDS to enhance regioselectivity, but only decomposition was observed. These results are summarized in the Supporting Information (Table S1).

We concluded that the failure of Route 1 stemmed from two factors: (1) quinine was insufficiently basic to deprotonate compound **5**, and (2) formation of the indolenine intermediate from a Boc-protected indole was difficult. Thus, we turned to Route 2, which does not require a strong base.

Route 2 began with the successful construction of diketopiperazine **6** using established procedures with minor modifications (Scheme 4).^14^ Notably, we substituted an Fmoc group in place of a Cbz group used in the original protocol. Alkylation of **6** with **3** was attempted under the conditions of Dubey and Olenyuk (ACN, reflux, quinine).^10^ Gratifyingly, the reaction proceeded smoothly, yielding the desired product with concurrent Boc deprotection (Table 1, entry 1). This outcome supports the hypothesis that Boc deprotection is a prerequisite for successful coupling, as the Boc group may hinder indolenine formation by blocking lone pair delocalization from the nitrogen.

**Scheme 4:**
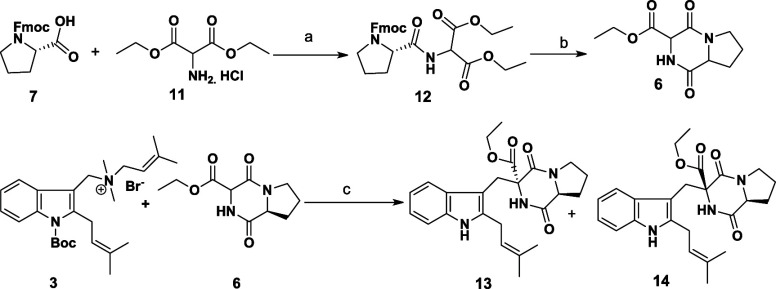
Preparation of Compound **6** and Coupling Reaction between Compounds **3** and **6** in the Presence of Quinine^a^

a Conditions: a) PyBOP, DIPEA, DCM, 78% yield b) 50% TEA, DCM, 80% yield c) **3** (2.0equiv), **6** (1.0 equiv), quinine (1.0 equiv), ACN, reflux, 12–48 h, 88% yield.

**Table 1:** Screening of Solvents and Bases for the Coupling of **3** and **6**

entry	solvent	base	equiv. base	time (h)	% conversion	diastereomeric ratio (**13**:**14**)
1	ACN	quinine	1.0	48	100	1:4
2	toluene	quinine	1.0	48	0	
3	1,4-dioxane	quinine	1.0	48	0	
4	DMF	quinine	1.0	48	100	1:4
5	DCM	quinine	1.0	48	0	
6	ACN	quinidine	1.0	48	100	1:4
7	ACN	quinine	0.2	48	0	
8	ACN	quinine	0.5	48	50	
9	ACN	quinine	1.0	24	100	1:2
10	ACN	quinine	1.0	120	100	1:4

We next evaluated alternative solvents (toluene, 1,4-dioxane, DMF, and DCM). Among these, only DMF supported product formation (Table 1, entry 4). Subsequently, various bases were screened to improve conversion or diastereoselectivity. The bases tested were quinidine, triethylamine (TEA), 1,8-diazabicyclo[5.4.0]­undec-7-ene (DBU), K_2_CO_3_, KOH, and NaH. Of these bases, only quinidine produced product, with nearly identical results to quinine (Table 1, entry 6; Table S2). Testing substoichiometric amounts of quinine (0.2 and 0.5 equiv) revealed that 1.0 equiv was required for full conversion (Table 1, entries 7–8). Finally, reaction time was optimized: shorter reaction time (24 h) favored a 1:2 diastereomeric ratio (**13**:**14**), while extended time (120 h) had no further impact (Table 1, entries 9–10).

The successful use of quinine and quinidine suggests their bifunctional role as bases generating enolates from compound **6** and as proton shuttles facilitating indolenine formation from gramine salt **3**. Such dual activity has been previously reported in asymmetric syntheses.^15^ A proposed transition state, shown in Scheme 5, depicts the tertiary amine of quinine abstracting a proton to form the enolate, while its protonated form aids Boc deprotection and promotes indolenine generation. Additionally, hydrogen bonding between the quinine hydroxyl group and the enolate likely helps preorganize the reactive species for coupling.

**Scheme 5:**
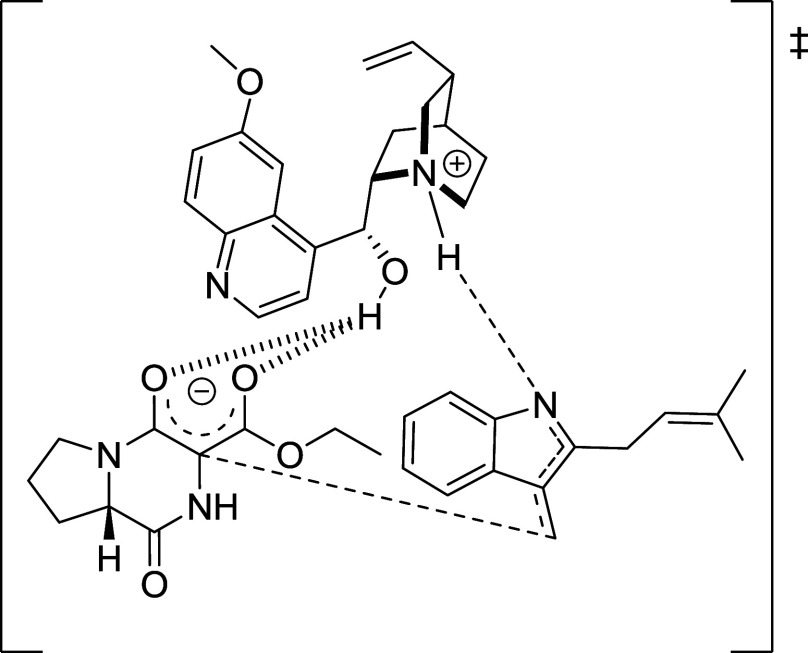
Plausible Transition State Showing the Enolate from **6** Attacking the Indolenine from **3** ^a^

a Dashed bonds represent partially formed or partially broken bonds. Hashed bonds represent hydrogen bonds.

This mechanism involves quinine’s tertiary amine acting as a base to generate the enolate from **6**, with the conjugate acid facilitating Boc removal from **3** and the concurrent formation of the reactive indolenine intermediate. The quinine hydroxyl group is proposed to form a hydrogen bond that positions the enolate in close proximity to the indolenine, thereby facilitating C–C bond formation with modest diastereoselectivity.

Notably, both quinine and its enantiomer quinidine yield the same diastereomeric ratio, indicating that stereoselectivity is likely not governed by the chiral base. Instead, it appears to originate from the inherent chirality of compound **6**. In particular, steric hindrance from the pyrrolidine ring in **6** may favor selective approach of gramine **3** to one face, promoting formation of diastereomer **14** over **13.**

During purification, we successfully separated the diastereomers by column chromatography; however, determination of the absolute stereochemistry of compounds **13** and **14** remained necessary. Attempts to grow X-ray quality crystals were unsuccessful. Consequently, we employed two-dimensional nuclear magnetic resonance (2D NMR) spectroscopy to assign the configurations.

Key structural insights were obtained from nuclear Overhauser effect spectroscopy (NOESY) of compound **14**. The NOESY spectrum showed through-space correlations between *H*15′ of the proline ring and both *H*4 and *H*5 of the indole moiety. In contrast, the diastereotopic proton *H*15, located on the opposite face of the ring, exhibited no such correlations (Figure 1). The numbering of compounds **13** and **14** can be seen in Figure 2. These results suggest that compound **14** adopts a folded conformation, bringing the indole and proline rings into spatial proximity. This folding places *H*4 and *H*5 near *H*15′, enabling the observed NOE cross-peaks.

**Figure 1:**
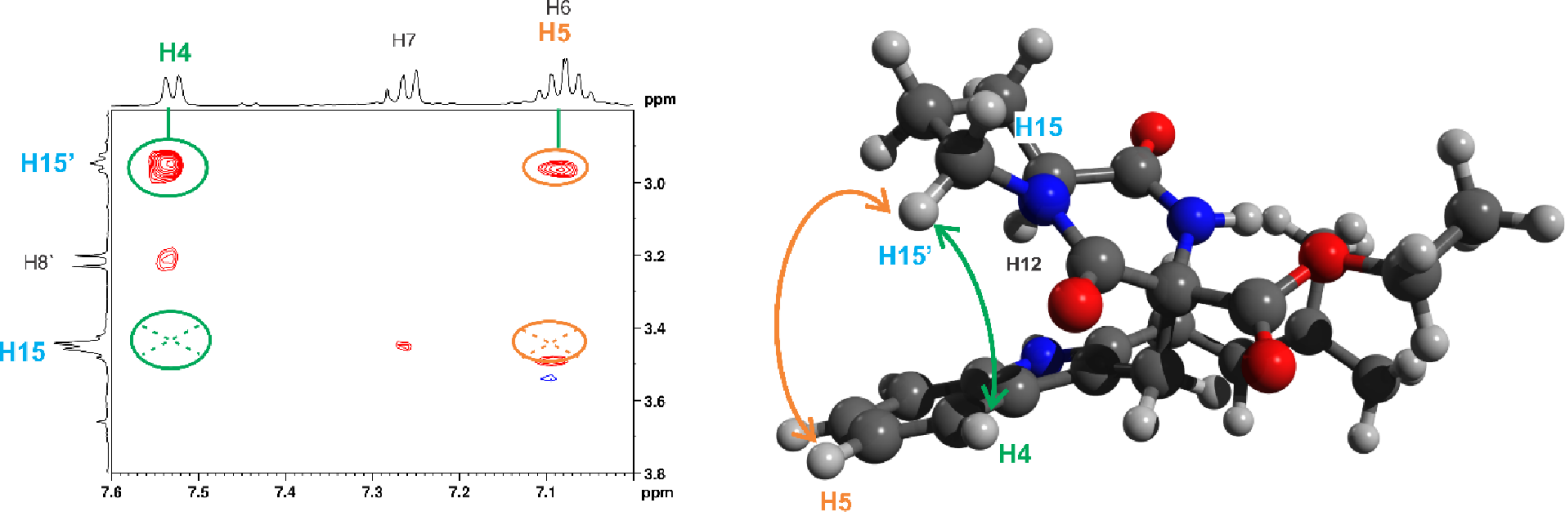
NOESY of **14** showing through space coupling for H4–H15’ and H5–H15’ (circles) and lack of through space coupling for H4–H15 and H5–H15 (crosses).

**Figure 2:**
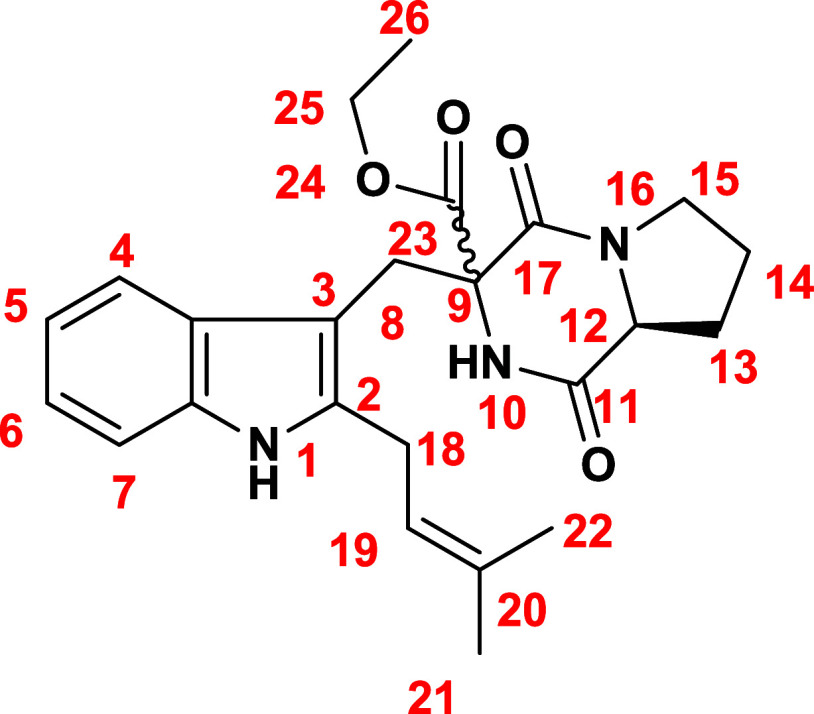
Numbered structure of compound **13**/**14**.

Further support for this assignment came from heteronuclear single quantum coherence (HSQC) spectroscopy, which enabled confident identification ∼ 4 ppm in compound **13** to ∼ 2 ppm in **14**—indicating of *H*12 as the α-proton of the proline residue. Notably, the chemical shift of *H*12 changed significantly—from a pronounced shielding effect in the folded structure.

This shift is consistent with the pyrrole ring being positioned close to the indole’s benzene moiety, inducing strong ring current effects.^16^ Similar upfield shifts were observed for all methylene protons in the proline ring.

Stereospecific assignments of these CH_2_ protons, based on local NOEs and scalar couplings, revealed that those oriented “down,” in the same direction as *H*12, experienced greater shielding due to proximity to the indole moiety. Taken together, these data support assignment of compound **13** as the (S,S) diastereomer and compound **14** as the (R,S) diastereomer.

Having synthesized compounds **13** and **14**, which contain the complete carbon framework of tryprostatin B (**2**), we proceeded to remove the carboethoxy group to complete the total synthesis of TPS B. Previously, Yamakawa et al. reported the conversion of compounds similar to **13** and **14** into TPS B using Krapcho decarboxylation, achieving a 1:1 ratio of TPS B and its 9-epimer when employing DMSO as the solvent and LiCl as a chloride source.^3i^

To improve the product ratio, we investigated the Krapcho decarboethoxylation of **13** and **14** independently using a range of solvents. This approach allowed us to determine whether separation of the isomers was necessary prior to decarboxylation or if a mixture could be used directly. We evaluated various solvents, ranging from polar aprotic to nonpolar, all in the presence of LiCl.

For compound **13**, complete conversion (100%) was achieved after 12 h of reflux in polar aprotic solvents such as DMSO, DMF, and DMA (Table 2, entries 1–3). Notably, in DMSO, the reaction proceeded to completion in just 30 min at 160 °C. Surprisingly, the nonpolar solvent o-xylene also afforded full conversion of **13** under 12-h reflux conditions (Table 2, entry 4).

**Table 2:** Optimization of Decarboethoxylation (Solvent Screening)

entry	substrate	solvent	% conversion^a^
1	**13**	DMSO	100^b^
2	**13**	DMF	100^c^
3	**13**	DMA	100^c^
4	**13**	o-xylene	100^c^
5	**14**	DMSO	100^b^
6	**14**	DMF	50^c^
7	**14**	DMA	100^c^
8	**14**	o-xylene	50^c^

a Confirmed by LC-MS.

b The reaction was performed at 160 °C for 0.5 h.

c The reaction was performed at reflux for 12 h.

In contrast, compound **14** displayed greater stability. It underwent complete conversion only in DMSO and DMA (Table 2, entries 5 and 6), while in DMF and o-xylene, only 50% conversion was observed (Table 2, entries 7 and 8). These results indicate that **14** is significantly more resistant to decarboxylation under these conditions than **13.**

It is noteworthy that during our solvent screening, both product isomers were obtained with minimal selectivity. However, this lack of selectivity is not considered a limitation in our current study. The formation of two separable diastereomers provides an advantage, as it increases the number of structurally related compounds available for future cytotoxicity assays (to be reported in a future publication). The observed differences in reactivity between compounds **13** and **14** are likely attributable to their distinct conformational folding, as discussed earlier. Moreover, solvents with higher boiling points, such as DMSO and DMA, facilitated complete conversion of both isomers, whereas solvents with lower boiling points, such as DMF and o-xylene, showed reduced conversion. Since DMSO and DMA promoted full conversion of both **13** and **14**, we proceeded with the reaction using a mixture of isomers without prior separation for subsequent experiments.

After completing the solvent screening, we next evaluated the effect of various salts on the Krapcho decarboxylation. These experiments were primarily conducted using DMSO as the solvent due to its shorter reaction time and consistent 100% conversion. Among the salts tested, NaCl produced a similar product distribution (1:2 ratio of TPS B to epi-TPS B) as LiCl, but the overall yield was significantly higher with LiCl (Table 3, entries 2 and 6). Other salts such as NaI, MgCl_2_, NaBr, and KBr either led to decomposition of the starting material or significantly reduced product yields.

**Table 3:** Optimization of Decarboethoxylation (Salt Screening)

entry	salt (5 equiv)	product ratio (**2**:**15**)	% conversion
1	NaI	NA	decomposed^a^
2	NaCl	1:2	10^a^
3	MgCl_2_	1:3	50^a^
4	NaBr	NR	decomposed^a^
5	KBr	ND	trace^a^
6	LiCl	1:2	76^a^
7	LiCl	1:1	89^b^

a The reaction was performed at 160 °C for 0.5 h in DMSO.

b The reaction was performed at reflux for 12 h in DMA.

Ultimately, we identified the optimal conditions using LiCl as the salt and DMA as the solvent (Table 3, entry 7). This combination gave the best product distribution (1:1 ratio of TPS B to epi-TPS B) along with a superior yield of 89%, compared to 76% yield with DMSO/LiCl under similar conditions (1:2 product ratio). These optimized conditions enabled a concise and efficient total synthesis of tryprostatin B (**2**) and its epimer **15** in just four steps, achieving a combined overall yield of 44% with a 1:1 diastereomeric ratio.

Using a similar synthetic route, tryprostatin A (**1**) was also synthesized, resulting in a 1:1 mixture of **1** and its 9-epimer **16**, with a combined overall yield of 22% (Scheme 6).

**Scheme 6:**
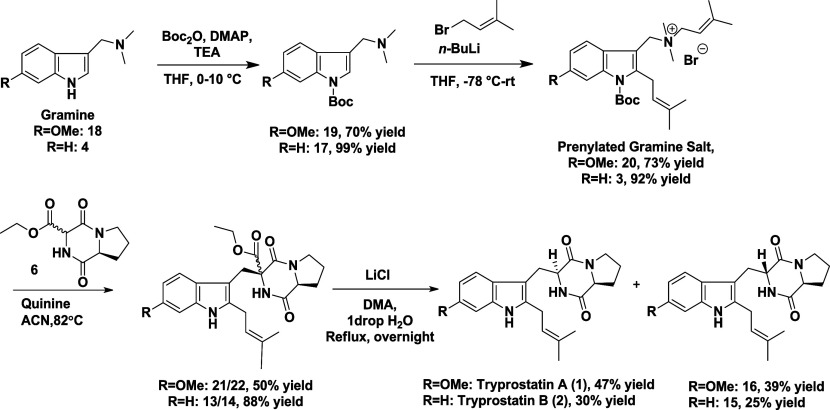
Total Synthesis of Tryprostatins A and B

## Conclusions

In summary, we have developed an exceptionally concise total synthesis of both tryprostatin A (**1**) and tryprostatin B (**2**). The key transformation in this approach was a quinine mediated coupling between a diketopiperazine (***6***) and a diprenylated gramine salt (**3**). Using this strategy, we synthesized TPS A and TPS B in only four steps, achieving an overall yield of 22% for TPS A and its epimer, and 44% for TPS B and its epimer. Both natural products were obtained as 1:1 mixtures with their respective epimers, which were successfully separated by column chromatography.

Looking ahead, we plan to leverage this streamlined synthetic route to prepare a diverse library of tryprostatin analogs for biological evaluation. These analogs, including compounds structurally related to **13** and **14**, will be explored for their potential anticancer properties.

## Experimental Section

All reactions were performed under a dry nitrogen atmosphere using standard Schlenk techniques unless otherwise noted. All reaction vessels were flame-dried under vacuum and filled with nitrogen prior to use. Reagents and solvents were purchased from Sigma-Aldrich, Milwaukee. All ^1^H and ^13^C NMR spectra were recorded in CDCl_3_ (internal standard: 7.26 ppm, ^1^H; 77.16 ppm, ^13^C­{^1^H}) at room temperature with a Bruker 300 MHz, 400 or 500 MHz spectrometers. The chemical shifts (δ) are given in parts per million (ppm) and the coupling constants in Hertz (Hz). The following abbreviations are used: s-singlet, d-doublet, t-triplet, q-quartet, m-multiplet. Previously reported compounds were identified by ^1^H NMR. All new compounds were additionally characterized by ^1^H NMR, ^13^C NMR and high-resolution mass spectrometry (HRMS). HRMS were obtained using Shimadzu LCMS-IT-TOF by electrospray ionization (ESI) technique. For the column chromatography, silica gel (35–70 μm) was used. The thin layer chromatography (TLC) was performed on aluminum backed plates precoated (0.25 mm) with Silica Gel 60 F254 with a suitable solvent system and was visualized using UV fluorescence and/or iodine chamber.

### (*S*)-(9*H*-Fluoren-9-yl)­methyl 2-((2-Ethoxy-2-oxoethyl)­carbamoyl)­pyrrolidine-1-carboxylate **(9)**

PyBOP (1.84 g, 3.55 mmol) and DIPEA (1.55 mL, 8.88 mmol) were added to a solution of Fmoc- *L*- proline **7** (1.0 g, 2.96 mmol) and glycine ethyl ester hydrochloride **8** (0.413 g, 2.96 mmol) in CH_2_Cl_2_ (25 mL). The reaction was stirred overnight, and progress was monitored by TLC. After consumption of the starting material, as judged by TLC analysis, the reaction was concentrated under reduced pressure. The residue was then dissolved in EtOAc (10 mL) and stirred via a stir bar until it made a homogeneous solution. The mixture was then acidified with 0.1 M HCl acid, extracted with water (3 × 30 mL) and then with NaHCO_3_ (3 × 30 mL). The combined organic layers were washed with brine solution (1 × 30 mL) and dried over anhydrous Na_2_SO_4_ and evaporated *in vacuo* to obtain crude product. The residue was purified with flash column chromatography on silica gel (Hexane/EtOAc = 1/1) to afford **9** as a white foam (1.12 g, 90%). Compound **9** was confirmed by comparing spectra to known NMR.^8b 1^H NMR (CDCl_3_, 300 MHz): ^1^H NMR (500 MHz, CDCl_3_) δ 7.69 (d, *J* = 7.5 Hz, 2H), 7.53–7.47 (m, 2H), 7.33 (t, *J* = 7.4 Hz, 2H), 7.24 (t, *J* = 7.5 Hz, 2H), 6.98 (s, 1H), 4.43–4.03 (m, 6H), 4.00–3.71 (m, 2H), 3.47–3.36 (m, 2H), 2.30–1.84 (m, 4H), 1.19 (t, *J* = 7.1 Hz, 3H). HRMS (ESI+): Calculated (*m*/*z*) for C_24_H_26_N_2_O_5_ (M+H)^+^: 423.1914, Found 423.1921.

### (*S*)-Hexahydropyrrolo­[1,2-*a*]­pyrazine-1,4-dione (**10**)

8.0 g of (S)-(9H-fluoren-9-yl)­methyl 2-((2-ethoxy-2-oxoethyl)­carbamoyl)­pyrrolidine-1-carboxylate **9** was dissolved in 100 mL of CH_2_Cl_2_ and 20% piperidine was added to it at room temperature. The reaction was stirred for 1.5 h, and progress was monitored by TLC. After consumption of the starting material, as judged by TLC analysis, the reaction was concentrated under reduced pressure. The residue was purified with flash column chromatography on silica gel (DCM/MeOH = 20/1) to afford **10** as a white solid (1.12 g, 80%). Compound **10** was confirmed by comparing spectra to known NMR.^5^ ^1^H NMR (CDCl_3_, 300 MHz): δ 7.10 (s, 1H), 4.10 (d, *J* = 15.0 Hz, 2H), 3.93 (dd, *J* = 15.0, 5.0 Hz, 1H), 3.65–3.56 (m, 2H), 2.40–2.34 (m, 1H), 2.09–1.90 (m, 3H), ^13^C NMR (CDCl_3_,75 MHz): δ 170.1, 163.5, 58.5, 46.6, 45.3, 28.5, 22.4. HRMS (ESI+): Calculated (*m*/*z*) for C_7_H_10_N_2_O_2_ (M+H)^+^:155.0815, Found 155.0819.

### (*S*)-*tert*-Butyl 1,4-Dioxohexahydropyrrolo­[1,2-*a*]­pyrazine-2­(1*H*)-carboxylate (**5**)

A solution of Boc anhydride (Boc_2_O) (21.3 g, 97.7 mmol), 4-dimethylaminopyridine (DMAP) (3.4 g, 27.9 mmol), trimethylamine (TEA) (2.8 mL, 27.9 mmol) in CH_3_CN (100 mL) was maintained at 0 °C for 30 min. A solution of (S)-hexahydropyrrolo­[1,2-*a*]­pyrazine-1,4-dione **10** (4.3 g, 27.9 mmol) in CH_3_CN (20 mL) was added dropwise through the dropping funnel over a period of 30 min at 0 °C. The reaction was stirred for 12 h, and progress was monitored by TLC. After consumption of the starting material, as judged by TLC analysis, the reaction was concentrated under reduced pressure and purified by column chromatography on silica gel (DCM/MeOH = 20/1) to give product **5** as a white solid (6.02 g, 85%). Compound **5** was confirmed by comparing spectra to known NMR.^9^ ^1^H NMR (CDCl_3_, 500 MHz): δ 4.67 (d, *J* = 15.6 Hz, 1H), 4.17 (t, *J* = 7.6 Hz, 1H), 4.11­(d, *J* = 14.8 Hz, 1H), 3.58 (t, *J* = 6.7, 2H), 2.38–2.26 (m, 2H), 2.04–1.93 (m, 2H), 1.54 (s, 9H); ^13^C NMR (CDCl_3_, 125 MHz): δ 167.5, 163.3, 150.1, 84.6, 60.3, 49.8, 45.2, 28.1, 23.1; HRMS (ESI+): Calculated (*m*/*z*) for C_12_H_18_N_2_O_4_ (M+Na)^+^: 277.1159, Found 277.1136.

### (*S*)-Diethyl 2-(1-(((9*H*-Fluoren-9-yl)­methoxy)­carbonyl)­pyrrolidine-2-carboxamido)­malonate (**12**)

PyBOP (18.43 g, 35.4 mmol) and DIPEA (11.5 mL, 88.9 mmol) were added to a solution of Fmoc- *L*- proline **7** (9.92 g, 29.4 mmol) and diethyl aminomalonate hydrochloride **11** (6.24 g, 29.4 mmol) in CH_2_Cl_2_ (100 mL). The reaction was stirred overnight, and progress was monitored by TLC. After consumption of the starting material, as judged by TLC analysis, the reaction was concentrated under reduced pressure. The residue was then dissolved in EtOAc (10 mL) and stirred via a stir bar until it made a homogeneous solution. The mixture was then acidified with 0.1 M HCl acid, extracted with water (3 × 30 mL) and then with NaHCO_3_ (3 × 30 mL). The combined organic layers were washed with brine solution (1 × 30 mL) and dried over anhydrous Na_2_SO_4_ and evaporated *in vacuo* to obtain crude product. The residue was purified with flash column chromatography on silica gel (Hexane/EtOAc = 2/5) to afford **12** as a white foam (10.9 g, 75%). ^1^H NMR (300 MHz, CDCl_3_) δ 7.75 (d, *J* = 7.5 Hz, 2H), 7.59 (s, 2H), 7.39 (t, *J* = 7.4 Hz, 2H), 7.30 (t, *J* = 7.2 Hz, 2H), 6.98 (s, 1H), 5.13 (d, *J* = 6.9 Hz, 1H), 4.46–4.08 (m, 8H), 3.64–3.48 (m, 2H), 2.32–2.15 (m, 2H), 2.01–1.87 (m, 2H), 1.26 (t, *J* = 7.1 Hz, 6H). ^13^C NMR (126 MHz, CDCl_3_) δ 172.3, 171.8, 166.1, 143.8, 141.2, 127.6, 127.0, 125.1, 119.9, 67.8, 62.3, 60.1, 56.2, 47.1, 28.8, 24.4, 23.4, 13.9. HRMS (ESI+): Calculated (*m*/*z*) for C_27_H_30_N_2_O_7_ (M+H)^+^: 495.2126, Found 495.2138.

### (8*aS*)-Ethyl 1,4-Dioxooctahydropyrrolo­[1,2-*a*]­pyrazine-3-carboxylate (**6**)

8.0 g of (S)-diethyl 2-(1-(((9H-fluoren-9-yl)­methoxy)­carbonyl)­pyrrolidine-2-carboxamido)­malonate **12** was dissolved in 100 mL of CH_2_Cl_2_ and 50% Triethyl amine­(TEA) was added to it at room temperature. The reaction was stirred for 12 h, and progress was monitored by TLC. After consumption of the starting material, as judged by TLC analysis, the reaction was concentrated under reduced pressure. The residue was purified with flash column chromatography on silica gel (DCM/MeOH = 20/1) to afford **6** as a white solid (2.19 g, 65%). Compound **6** was confirmed by comparing spectra to known NMR.^17^ ^1^H NMR (CDCl_3_, 500 MHz): δ 7.59 (d, *J* = 3.5 Hz, 1H), 4.65 (d, *J* = 4.4 Hz, 1H), 4.32–4.18 (m, 3H), 3.62–3.51 (m, 2H), 2.39–2.33 (m, 1H), 2.05–1.98 (m, 2H), 1.92–1.85 (m, 1H), 1.30 (t, *J* = 7.2 Hz, 3H). ^13^C NMR (CDCl_3_,125 MHz): δ 170.5, 166.9, 159.6, 63.0, 60.9, 58.5, 46.0, 28.6, 22.4, 14.1. HRMS (ESI+): Calculated (*m*/*z*) for C_7_H_10_N_2_O_2_ (M-H): 225.0881, Found 225.0876.

### *t*-Butyl 3-((Dimethylamino)­methyl)-1*H*-indole-1-carboxylate (**17**)

A solution of Boc anhydride (Boc_2_O) (12.0 g, 55.0 mmol), 4-dimethylaminopyridine (DMAP) (0.56 g, 4.58 mmol), trimethylamine (TEA) (0.56 mL, 4.02 mmol) in THF (100 mL) was maintained at 0 °C for 30 min. A solution of gramine **4** (8.0 g, 45.9 mmol) in THF (60 mL) was added dropwise through the dropping funnel over a period of 30 min at 0 °C. The reaction mixture was stirred at 0 °C for 1.5 h under nitrogen atmosphere. After consumption of starting material, as judged by TLC analysis, water (20 mL) was added to the reaction mixture. Next, THF was removed under reduced pressure and then the aqueous layer was extracted with ether (3 × 30 mL), washed with brine (1 × 30 mL). The combined organic layer was dried over anhydrous Na_2_SO_4_. The crude product was purified with column chromatography on silica gel (hexane/EtOAc = 7/3) to give product **17** as a light brown solid (12.5 g, 99%). Compound **17** was confirmed by comparing spectra to known NMR.^5^ ^1^H NMR (CDCl_3_, 500 MHz): δ 8.16 (s, 1H), 7.69 (d, *J* = 7.8 Hz, 1H), 7.54 (br s, 1H), 7.34 (t, *J* = 7.5 Hz, 1H), 7.25 (t, *J* = 7.5 Hz, 1H), 3.60 (s, 2H), 2.33 (s, 6H), 1.69 (s, 9H).

### *N*-((1-(*tert*-Butoxycarbonyl)-2-(3-methylbut-2-en-1-yl)-1*H*-indol-3-yl)­methyl)-*N*,*N*,3-trimethylbut-2-en-1-aminium Bromide (**3**)

A solution of *Tert*-butyl 3-((dimethylamino)­methyl) −1H-indole-1-carboxylate 17 (8.0 g, 29.2 mmol) in THF (100 mL) was taken in three necked round bottomed flask and nitrogen was bubbled through the solution for 20 min. This mixture was cooled to −78 °C and *n*-butyl lithium (23.3 mL, 2.5 M, 58.3 mmol) was added dropwise to the reaction mixture maintaining a temperature −78 °C over a period of 1 h under nitrogen atmosphere. Prenyl bromide (17.4 mL, 150.6 mmol) was added to the reaction dropwise through the dropping funnel over a period of 30 min. The reaction mixture was allowed to warm to room temperature and was stirred overnight. After consumption of the starting material, as judged by TLC analysis, water (30 mL) was added to the reaction mixture and THF was removed under reduced pressure. The mixture was then extracted with CH_2_Cl_2_ (3 × 30 mL), the combined organic layers were washed with brine solution (1 × 30 mL) and dried over anhydrous Na_2_SO_4_ and evaporated *in vacuo* to obtain crude product. The residue was purified with flash column chromatography on silica gel (DCM/MeOH = 20/1) to afford **3** as a brown solid (13.2 g, 92%). Compound **6** was confirmed by comparing spectra to known NMR.^7^ ^1^H NMR (500 MHz, CDCl_3_) δ 8.02–7.97 (m, 2H), 7.24–7.20 (m, 2H), 5.30 (t, *J* = 8.2 Hz, 1H), 5.20 (s, 2H), 4.95 (t, *J* = 5.6 Hz, 1H), 4.48 (d, *J* = 8.1 Hz, 2H), 3.82 (s, 2H), 3.08 (s, 6H), 1.84 (s, 3H), 1.79 (s, 3H), 1.71 (s, 3H), 1.58 (s, 12H).

### (3*S*,8*aS*)-Ethyl 3-((2-(3-Methylbut-2-en-1-yl)-1*H*-indol-3-yl)­methyl)-1,4-dioxooctahydropyrrolo­[1,2-*a*]­pyrazine-3-carboxylate (**13**) and (3R,8*aS*)-Ethyl 3-((2-(3-Methylbut-2-en-1-yl)-1*H*-indol-3-yl)­methyl)-1,4-dioxooctahydropyrrolo­[1,2-*a*]­pyrazine-3-carboxylate (**14**)

To a suspension of *N*-((1-(tert-butoxycarbonyl)-2-(3-methylbut-2-en-1-yl)-1H-indol-3-yl)­methyl)-N,N,3-trimeth-ylbut-2-en-1-aminium bromide **3** (1.30 g, 2.64 mmol) in acetonitrile (5.0 mL) compound (8aS)-ethyl 1,4-dioxooctahydropyrrolo­[1,2-*a*]­pyrazine-3-carboxylate **6** (300 mg, 1.30 mmol) was added, followed by the addition of quinine (430 mg, 1.30 mmol). The mixture was stirred under reflux for 24–48 h and monitored by TLC. The solvent was removed in vacuo. The crude product was purified with flash column chromatography on silica gel (DCM/MeOH = 100/1) to afford **13** as a white solid (98.9 mg, 17.6%). ^1^H NMR (CDCl_3_, 500 MHz): δ 8.07 (br s, 1H), 7.46 (d, *J* = 7.9 Hz, 1H), 7.30 (d, *J* = 8.0 Hz, 1H), 7.15 (t, *J* = 7.5 Hz, 1H), 7.09 (t, *J* = 7.5 Hz, 1H), 6.04 (br s, 1H), 5.30­(t, *J* = 7.5 Hz, 1H), 4.25 (q, *J* = 7.5 Hz, 2H), 4.05–4.01­(m, 2H), 3.73–3.59 (m, 2H), 3.53–3.40 (m, 3H), 2.41–2.36 (m, 1H), 2.07–1.99 (m, 2H), 1.94–1.84 (m, 1H), 1.81­(s, 3H), 1.76­(s, 3H), 1.26­(t, *J* = 7.5 Hz, 3H); ^13^C NMR (CDCl_3_, 75 MHz): δ170.2, 168.9, 162.6, 137.3, 135.7, 135.4, 128.6, 121.9, 120.1, 119.6, 118.2, 110.7, 102.6, 66.5, 63.1, 59.3, 46.2, 29.3, 28.8, 25.8, 25.2, 22.6, 17.9, 13.9; HRMS (ESI+): Calculated (*m*/*z*) for C_24_H_29_N_3_O_4_ (M+Na)^+^: 446.2050, Found 446.2057. Further elution with (DCM/MeOH = 50/1) gave **14** as a white solid (395.65 mg, 70.4%). The ratio between **13** and **14** was 1:4. ^1^H NMR (CDCl_3_, 500 MHz): δ 8.06 (br s, 1H), 7.54 (d, *J* = 10.0 Hz, 1H), 7.27 (d, *J* = 10.0 Hz, 1H), 7.12–7.07 (m, 2H), 6.52 (br s, 1H), 5.32 (t, *J* = 7.5 Hz, 1H), 4.38–4.34 (m, 2H), 3.99 (d, *J* = 15.0 Hz, 1H), 3.49–3.44 (m, 3H), 3.22 (d, *J* = 15.0 Hz, 1H), 2.96 (dd, *J =* 10.0 Hz, 1H), 2.06–2.03 (m, 1H), 1.91–1.86 (m, 1H), 1.81­(s, 3H), 1.76 (s, 3H), 1.73–1.69 (m, 1H), 1.65–1.57 (m, 1H), 1.38 (t, J = 7.5 Hz, 3H), 1.16–1.08 (m, 1H). ^13^C NMR (CDCl_3_, 75 MHz): δ 168.3, 168.2, 162.4, 137.5, 135.8, 134.8, 128.6, 121.6, 119.7, 119.4, 118.5, 110.4, 103.5, 69.3, 63.0, 57.8, 45.3, 31.59, 28.9, 25.8, 24.9, 21.3, 18.0, 14.1; HRMS (ESI+): Calculated (*m*/*z*) for C_24_H_29_N_3_O_4_ (M+H)^+^: 424.2231, Found 424.2220.

### Synthesis of Tryprostatin B (**2**) and 9-Epi-tryprostatin B (**15**)

A mixture of **13** and **14** (50 mg, 0.118 mmol), lithium chloride (32 mg, 0.37 mmol), and H_2_O (1 drop) in DMA (5.0 mL) was stirred for 12 h at reflux condition under nitrogen atmosphere. The resulting mixture was poured into brine and extracted with CHCl_3_. The extract was evaporated to afford a syrup, which was purified with flash column chromatography on silica gel (DCM/MeOH = 20/1) to afford tryprostatin B **2** (12.3 mg, 30%). Compound **2** was confirmed by comparing spectra to known NMR.^3k 1^H NMR (CDCl_3_, 500 MHz): δ 7.99 (brs, 1H), 7.50 (d, *J* = 10.0 Hz, 1H), 7.34 (d, *J* = 5.0 Hz, 1H), 7.19 (t, *J* = 7.5 Hz, 1H), 7.12 (t, *J* = 7.5 Hz, 1H), 5.64 (s, 1H), 5.33 (t, *J* = 7.5 Hz, 1H), 4.39 (dd, *J* = 10.0, 5.0 Hz, 1H), 4.08 (t, *J* = 7.5 Hz, 1H), 3.73–3.68 (m, 2H), 3.64–3.59 (m, 1H), 3.51 (t, *J* = 7.5 Hz, 2H), 3.01–2.95­(dd, *J* = 10.0, 5.0 Hz, 1H), 2.38–2.32 (m, 1H), 2.08–2.02 (m, 2H), 1.96–1.90 (m, 1H), 1.81 (s, 3H), 1.78 (s, 3H). Further elution with DCM/MeOH = 9/1 gave 9-epi-tryprostatin B **15** (10.2 mg, 25%). Compound **15** was confirmed by comparing spectra to known NMR.^3k 1^H NMR (CDCl_3_, 400 MHz) δ 7.96 (br s, 1H), 7.53 (d, *J =* 8.4 Hz, 1H), 7.15–7.07 (m, 2H), 6.04 (br s, 1H), 5.31 (t, *J* = 7.2 Hz, 1H), 4.26–4.23 (m, 1H), 3.62–3.56 (m, 1H), 3.54–3.49 (m, 1H), 3.44–3.38 (m, 3H), 3.18–3.11 (m, 2H), 2.70–2.67 (m, 1H), 2.04–2.00 (m, 1H), 1.90–1.65 (m, 2H), 1.81 (s, 3H), 1.77 (s, 3H), 1.38–1.34 (1H, m).

### *t*-Butyl 3-((Dimethylamino)­methyl)-6-methoxy-1*H*-indole-1-carboxylate **(19)**

A solution of Boc anhydride (Boc_2_O) (2.0 g, 8.81 mmol), 4-dimethylaminopyridine (DMAP) (0.09 g, 0.73 mmol), trimethylamine (TEA) (0.123 mL, 1.21 mmol) in THF (30 mL) was maintained at 0 °C for 30 min. A solution of 6-methoxy gramine **18** (1.5 g, 7.34 mmol) in THF (15 mL) was added dropwise through the dropping funnel over a period of 30 min at 0 °C. The reaction mixture was stirred at 0 °C for 1.5 h under nitrogen atmosphere. After consumption of starting material, as judged by TLC analysis, water (20 mL) was added to the reaction mixture. Next, THF was removed under reduced pressure and then the aqueous layer was extracted with ether (3 × 15 mL), washed with brine (1 × 15 mL). The combined organic layer was dried over anhydrous Na_2_SO_4_. The crude product was purified with column chromatography on silica gel (hexane/EtOAc = 1/1) to give product **19** as a light brown solid (1.56 g, 70%). ^1^H NMR (500 MHz, CDCl_3_) δ 7.77 (s, 1H), 7.56 (d, J = 8.6 Hz, 1H), 7.40 (s, 1H), 6.90 (dd, J = 8.6, 2.4 Hz, 1H), 3.89 (s, 3H), 3.52 (s, 2H), 2.30 (s, 6H), 1.69 (s, 9H); ^13^C NMR (CDCl_3_, 75 MHz): δ 157.9, 149.8, 136.6, 124.4, 123.0, 120.2, 118.2, 111.9, 99.3, 83.3, 55.6, 54.7, 45.6, 28.2; HRMS (ESI+): Calculated (*m*/*z*) for C_17_H_25_N_2_O_3_ (M+H)^+^: 305.1860, Found 305.1860

### *N*-((1-(*tert*-Butoxycarbonyl)-6-methoxy-2-(3-methylbut-2-en-1-yl)-1*H*-indol-3-yl)­methyl)-*N*,*N*,3-trimethylbut-2-en-1-aminium Bromide (**20**)

A solution of *Tert*-butyl 3-((dimethylamino)­methyl)-6-methoxy-1H-indole-1-carboxylate **19** (1.9 g, 6.3 mmol) in THF (60 mL) was taken in three necked round bottomed flask and nitrogen was bubbled through the solution for 20 min. This mixture was cooled to −78 °C and *n*-butyl lithium (5.1 mL, 2.5 M, 12.3 mmol) was added dropwise to the reaction mixture maintaining a temperature −78 °C over a period of 1 h under nitrogen atmosphere. Prenyl bromide (3.3 mL, 28.6 mmol) was added to the reaction dropwise through the dropping funnel over a period of 30 min. The reaction mixture was allowed to warm to room temperature and was stirred overnight. After consumption of the starting material, as judged by TLC analysis, water (20 mL) was added to the reaction mixture and THF was removed under reduced pressure. The mixture was then extracted with CH_2_Cl_2_ (3 × 15 mL), the combined organic layers were washed with brine solution (1 × 15 mL) and dried over anhydrous Na_2_SO_4_ and evaporated *in vacuo* to obtain crude product. The residue was purified with flash column chromatography on silica gel (DCM/MeOH = 20/1) to afford **20** as a brown solid (2.4 g, 73%). ^1^H NMR (500 MHz, CDCl_3_) δ 7.99 (d, *J* = 8.7 Hz, 1H), 7.63 (d, *J* = 2.4 Hz, 1H), 6.91 (dd, *J* = 8.7, 2.4 Hz, 1H), 5.36 (t, *J* = 8.3 Hz, 1H), 5.18 (s, 2H), 5.01 (t, *J* = 5.6 Hz, 1H), 4.50 (d, *J* = 8.2 Hz, 2H), 3.83 (s, 3H), 3.13 (s, 6H), 1.87 (s, 3H), 1.83 (s, 3H), 1.75 (s, 3H), 1.63 (s, 12H); ^13^C NMR (CDCl_3_, 75 MHz): δ 157.8, 149.8, 149.0, 142.7, 136.9, 134.0, 122.9, 120.6, 120.5, 112.5, 111.1, 106.0, 99.7, 84.9, 61.8, 58.4, 55.6, 48.4, 27.9, 26.9, 26.5, 25.5, 19.5, 18.7; HRMS (ESI+): Calculated (*m*/*z*) for C_27_H_41_N_2_O_3_ [M]^+^: 441.3112, Found: 441.3110.

### (3*S*,8*aS*)-Ethyl 3-((6-Methoxy-2-(3-methylbut-2-en-1-yl)-1*H*-indol-3-yl)­methyl)-1,4-dioxooctahydropyrrolo­[1,2-*a*]­pyrazine-3-carboxylate (**21**) and (3*R*,8*aS*)-Ethyl 3-((6-Methoxy-2-(3-methylbut-2-en-1-yl)-1*H*-indol-3-yl)­methyl)-1,4-dioxooctahydropyrrolo­[1,2-*a*]­pyrazine-3-carboxylate (**22**)

To a suspension of N-((1-(tert-butoxycarbonyl)-6-methoxy-2-(3-methylbut-2-en-1-yl)-1H-indol-3-yl)­methyl)-N,N,3-trimethylbut-2-en-1-aminium bromide **20** (230 mg, 0.442 mmol) in acetonitrile (10.0 mL) compound (8aS)-ethyl 1,4-dioxooctahydropyrrolo­[1,2-*a*]­pyrazine-3-carboxylate 6 (50 mg, 0.221 mmol) was added, followed by the addition of 86 mg (0.265 mmol) of quinine. The mixture was stirred under reflux for 24–48 h and monitored by TLC. The solvent was removed in vacuo. The crude product was purified with flash column chromatography on silica gel (DCM/MeOH = 100/1) to afford **21** as a white solid (9.9 mg, 10%). ^1^H NMR (500 MHz, CDCl_3_) δ 7.90 (s, 1H), 7.32 (d, *J* = 8.7 Hz, 1H), 6.82 (d, *J* = 1.9 Hz, 1H), 6.76 (dd, *J* = 8.7, 2.2 Hz, 1H), 6.04 (s, 1H), 5.29 (t, *J* = 7.2 Hz, 1H), 4.24 (q, *J* = 6.5 Hz, 2H), 4.05–4.01 (m, 1H), 3.98 (d, *J* = 15.3 Hz, 1H), 3.84 (s, 3H), 3.73–3.67 (m, 1H), 3.63–3.58 (m, 1H), 3.48–3.36 (m, 3H), 2.41–2.36 (m, 1H), 2.07–1.99 (m, 2H), 1.94–1.87 (m, 1H), 1.80 (s, 3H), 1.76 (s, 3H), 1.26 (m, 3H); ^13^C NMR (CDCl_3_, 126 MHz): δ168.30, 168.25, 162.4, 156.0, 136.2, 135.6, 135.5, 122.9, 119.6, 119.2, 109.2, 103.3, 94.3, 69.2, 63.0, 57.8, 55.7, 45.3, 33.0, 28.9, 25.8, 24.9, 21.3, 17.9, 14.1; HRMS (ESI+): Calculated (*m*/*z*) for C_25_H_31_N_3_O_5_ (M+Na)^+^: 476.2156, Found 476.2163. Further elution with (DCM/MeOH = 50/1) gave **22** as a white solid (39.6 mg, 39%). The ratio between **21** and **22** was 1:4. ^1^H NMR (500 MHz, CDCl_3_) δ 7.93 (s, 1H), 7.40 (d, *J* = 8.7 Hz, 1H), 6.78 (d, *J* = 2.1 Hz, 1H), 6.75 (dd, *J* = 8.7, 2.2 Hz, 1H), 6.53 (s, 1H), 5.29 (t, *J* = 7.3 Hz, 1H), 4.40–4.31 (m, 2H), 3.92 (d, *J* = 14.9 Hz, 1H), 3.83 (s, 3H), 3.51–3.45 (m, 1H), 3.42–3.39 (m, 2H), 3.16 (d, *J* = 14.9 Hz, 1H), 3.03–2.96 (m, 1H), 2.14 (dd, *J* = 11.1, 6.2 Hz, 1H), 1.94–1.89 (m, 1H), 1.79 (s, 3H), 1.75 (s, 3H), 1.68–1.59 (m, 2H), 1.37 (t, *J* = 7.1 Hz, 3H), 1.24–1.14 (m, 1H); ^13^C NMR (CDCl_3_, 126 MHz): δ 168.30, 168.25, 162.4, 156.0, 136.2, 135.6, 135.5, 122.9,, 119.6, 119.2, 109.2 103.3, 94.3, 69.2, 63.0, 57.8, 55.7, 45.3, 33.0, 28.9, 25.8, 24.9, 21.3,17.9, 14.1; HRMS (ESI+): Calculated (*m*/*z*) for C_25_H_31_N_3_O_5_ (M+H)^+^: 454.2337, Found 454.2325.

Synthesis of Tryprostatin A (**1**) and 9-epi-tryprostatin A (**16**). A mixture of **21** and **22** (50 mg, 0.118 mmol), lithium chloride (32 mg, 0.37 mmol), and H_2_O (1 drop) in DMA (5.0 mL) was stirred for 12 h at reflux condition under nitrogen atmosphere. The resulting mixture was poured into brine and extracted with CHCl_3_. The extract was evaporated to afford a syrup, which was purified by with flash column chromatography on silica gel (DCM/MeOH = 20/1) to afford tryprostatin A **1** (12.3 mg, 47%). Compound **1** was confirmed by comparing spectra to known NMR.^3k 1^H NMR (500 MHz, CDCl_3_) δ 7.85 (s, 1H), 7.36 (d, *J* = 8.6 Hz, 1H), 6.85 (d, *J* = 2.2 Hz, 1H), 6.78 (dd, *J* = 8.7, 2.3 Hz, 1H), 5.67 (s, 1H), 5.32 (t, *J* = 7.3 Hz, 1H), 4.36 (dd, *J* = 11.4, 2.5 Hz, 1H), 4.08 (t, *J* = 7.3 Hz, 1H), 3.85 (s, 3H), 3.73–3.58 (m, 3H), 3.45 (t, *J* = 8.8 Hz, 2H), 2.94 (dd, *J* = 15.1, 11.4 Hz, 1H), 2.38–2.32 (m, 1H), 2.10–1.89 (m, 3H), 1.80 (s, 3H), 1.77 (s, 3H); 13C NMR (CDCl_3_, 125 MHz): δ 169.4, 165.8, 156.4, 136.3, 135.3, 135.1, 122.3, 120.0, 118.4, 109.4, 104.5, 94.9, 59.3, 55.8, 54.6, 45.4, 29.7, 28.4, 25.8, 25.1, 22.7, 18.0. Further elution with DCM/MeOH = 9/1 gave 9-epi-tryprostatin A **16** (10.2 mg, 39%). Compound **16** was confirmed by comparing spectra to known NMR.^3k 1^H NMR (500 MHz, CDCl_3_) δ 7.86 (s, 1H), 7.39 (d, *J* = 8.7 Hz, 1H), 6.79 (d, *J* = 2.3 Hz, 1H), 6.76 (dd, *J* = 8.7, 2.3 Hz, 1H), 6.46 (d, *J* = 40.0 Hz, 1H), 5.29 (t, *J* = 7.3 Hz, 1H), 4.26 (q, *J* = 4.4 Hz, 1H), 3.83 (s, 3H), 3.58–3.52 (m, 1H), 3.42–3.36 (m, 3H), 3.16 (t, *J* = 10.8 Hz, 1H), 3.10 (dd, *J* = 14.8, 4.5 Hz, 1H), 2.68–2.64 (m, 1H), 2.07–2.02 (m, 1H), 1.80 (s, 3H), 1.76 (s, 3H), 1.72–1.63 (m, 1H), 1.45–1.35 (m, 2H); ^13^C NMR (126 MHz, CDCl_3_) δ 169.6, 165.7, 156.1, 135.8, 135.4, 135.3, 122.7, 119.8, 119.0, 109.2, 104.2, 94.3, 58.7, 57.7, 55.7, 45.1, 29.4, 29.0, 25.8, 24.9, 21.5, 17.9.

## Supplementary Material



## Notes

NMR spectra, MS spectra, and additional condition screening (PDF)
