# Supplementary material for: A Concise Total Synthesis of Tryprostatins A and B and Epimers
Source: ACS Omega. 2025 Oct 8;10(41):49109–17. doi: 10.1021/acsomega.5c07992 (PMC12547567; doi:10.1021/acsomega.5c07992)

Supporting Information

A concise Total Synthesis of Tryprostatins A, B and Epimers

Sophia Seideman [a], Khorshada Jahan [a], Frank Holger Foersterling [a], and M. Mahmun Hossain[a]*

[a]*Department of Chemistry and Biochemistry, University of Wisconsin-Milwaukee, 2000 E Kenwood Blvd, Milwaukee, Wisconsin 53211-3029, United States*

*E-mail: Mahmun@uwm.edu

Table of Contents:

1. Tables of Reaction Conditions..S2
2. NMR and HRMS spectra of synthesized compounds...S4

Table S1. Coupling reactions between 3 and 5.

Entry	Solvent	Base	Catalyst	Equiv. **3**	Equiv. **5**	Equiv. base	Time (hr)	Temp (ºC)	Yield of **2**/**16** (%)
1	DCM	KOH	Cat A	2.0	1.0	15	72	rt	0
2	DCM	KOH	Cat B	2.0	1.0	15	24	rt	0
3	1,4-dioxane	KOH	Cat A	2.0	1.0	15	72	rt	0
4	1,4-dioxane	KOH	Cat B	2.0	1.0	15	24	rt	0
5	THF	KOH	Cat A	2.0	1.0	15	72	rt	0
6	ACN	Quinine	-	2.0	1.0	1.0	12	80	0
7	DMF	NaH	-	2.0	1.0	2.0	12	50	0
8	ACN	NaH	-	2.0	1.0	1.5	12	80	0
9	ACN	NaH	-	1.5	1.0	1.5	12	80	0
10	ACN	NaH	-	1.0	1.0	1.5	12	80	0
11	ACN	NaH	-	2.0	1.0	4.5	12	80	0
12	ACN	NaHMDS	-	2.0	1.0	1.5	12	rt	0

Table S2: **Screening of bases for the coupling of 3 and 6.**

Entry	Solvent	Base	Equiv. Base	Time(hr)	Temp °C	% Conversion
1	ACN	TEA	1.0	48	Reflux	0
2	ACN	DBU	1.0	48	Reflux	0
3	ACN	K_2CO_3	1.0	48	Reflux	0
4	DCM/H_2O	K_2CO_3/Cat B	1.0	72	Rt	0
5	DCM/ H_2O	KOH/Cat B	1.0	72	Rt	0
6	DMF	NaH	1.2	24	Rt	0
7	DMF	NaH	1.2	72	Rt	0
8	DMF	NaH	1.2	6	55	0
9	DMF	NaH	1.2	24	55	0
10	DMF	NaH	1.2	48	55	0

^{1}H NMR of **17** (500 MHz, $CDCl_3$)

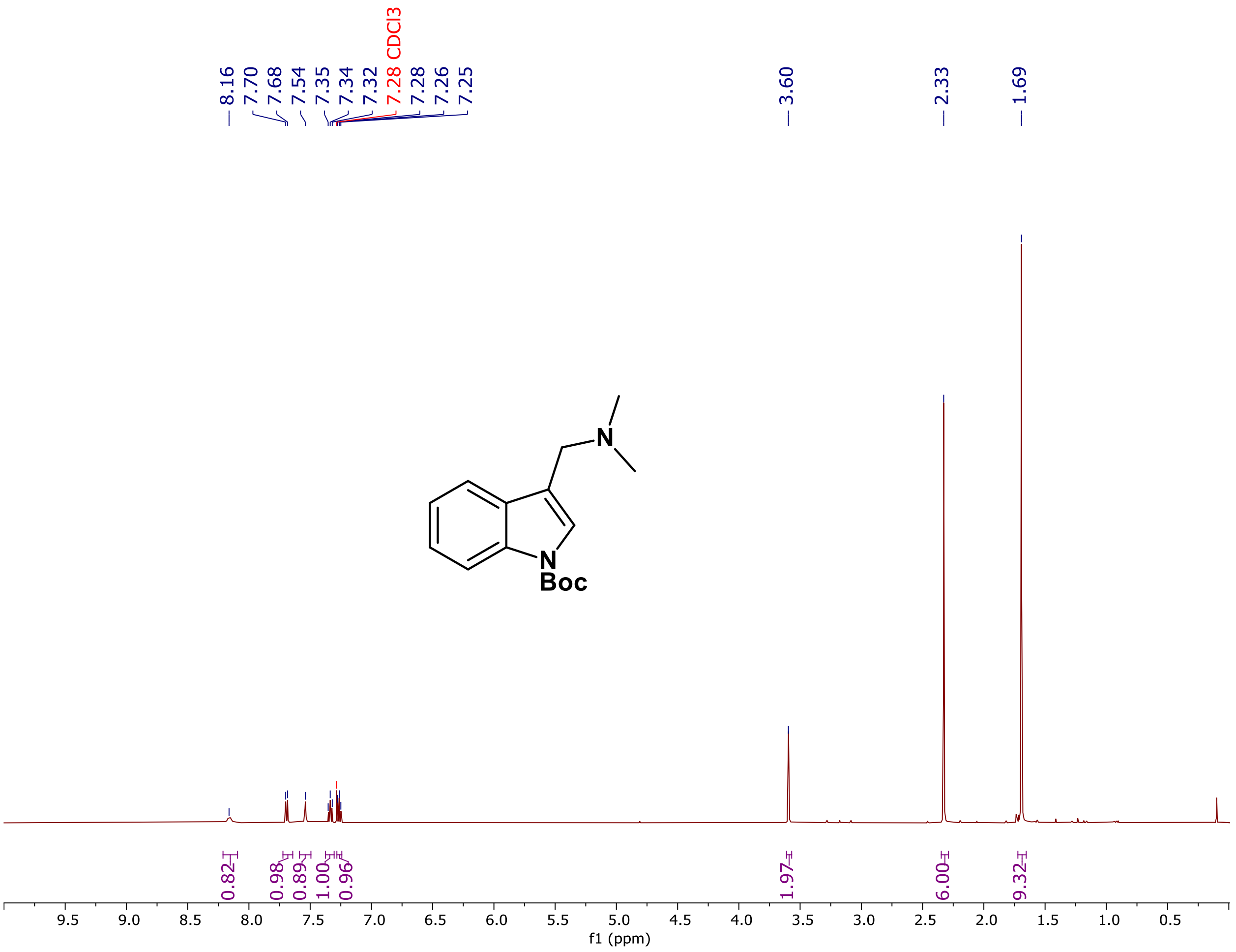

^{1}H NMR of **3** (500 MHz, $CDCl_3$)

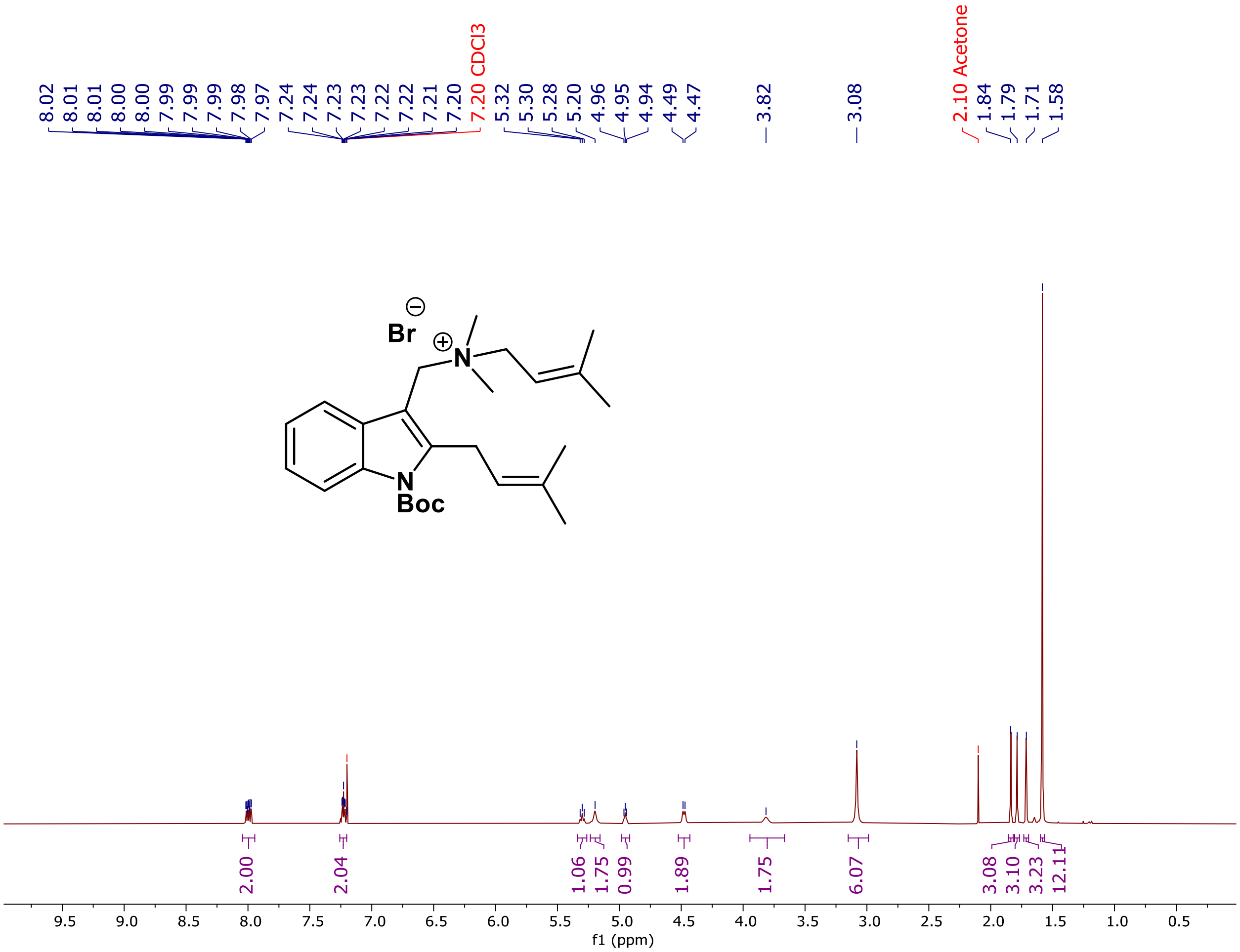

^{1}H NMR of **9** (500 MHz, $CDCl_3$)

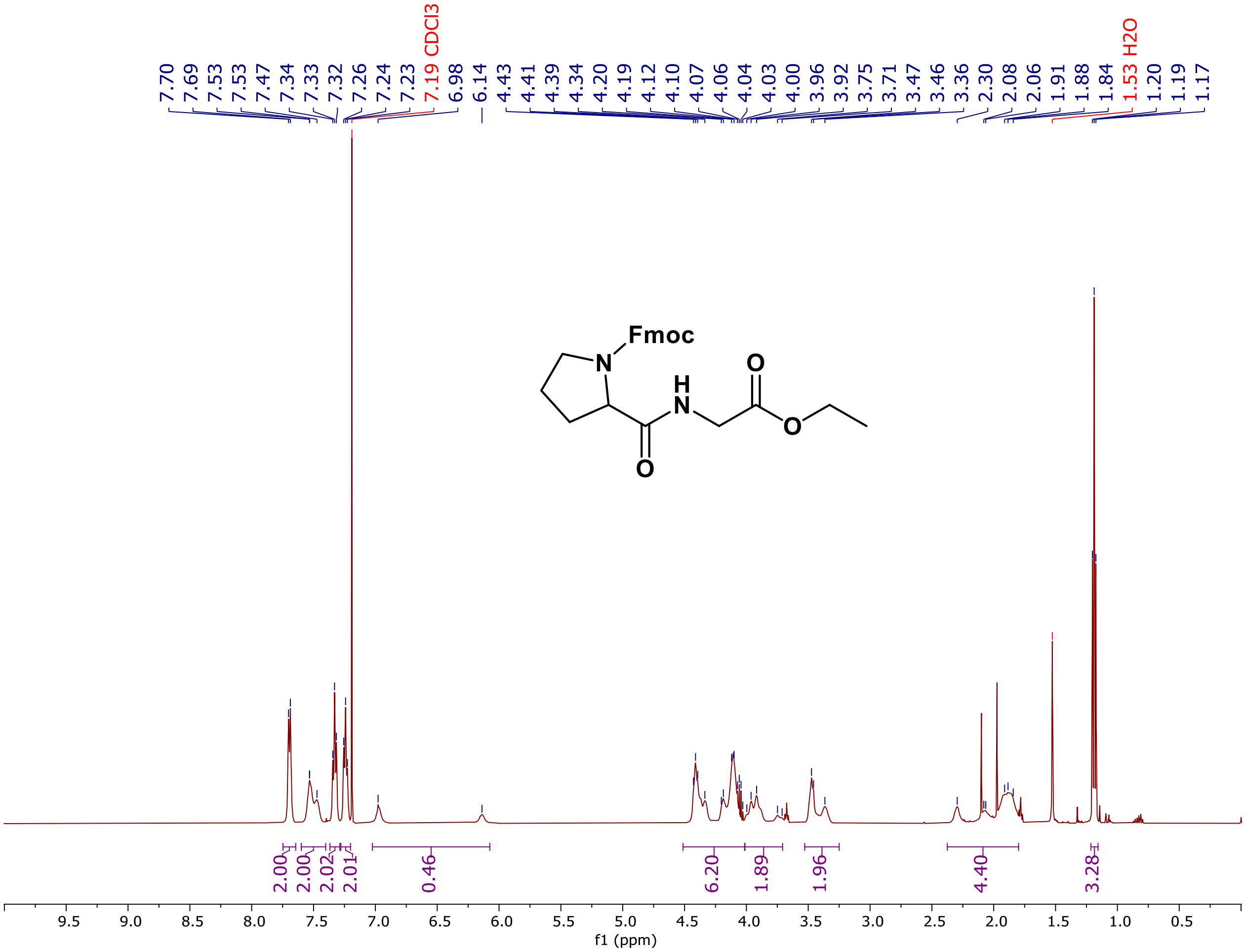

HRMS of **9**

Formula Predictor Report - CPD 1 422_06042021 Analysis HRMS_42.lcd

Data File: C:\LabSolutions\Data\Vilashini Rajaratnam\06042021 Analysis\CPD 1 422_06042021 Analysis HRMS_42.lcd

Elmt	Val.	Min	Max	Elmt	Val.	Min	Max	Elmt	Val.	Min	Max	Elmt	Val.	Min	Max	Use Adduct
H	1	25	35	O	2	5	9	P	3	0	0	I	3	0	0	H
2H	1	0	0	F	1	0	0	S	2	0	0					Na
C	4	20	35	Na	1	0	0	Cl	1	0	0					K
N	3	0	3	Si	4	0	0	Br	1	0	0					NH4

Error Margin (ppm): 500
HC Ratio: unlimited
Max Isotopes: all
MSn Iso RI (%): 75.00
DBE Range: -100.0 - 2000.0
Apply N Rule: no
Isotope RI (%): 1.00
MSn Logic Mode: AND
Electron Ions: both
Use MSn Info: yes
Isotope Res: 10000
Max Results: 10

Event#: 1 MS(E+) Ret. Time : 0.427 -> 0.840 - 0.133 -> 0.217 Scan# : 65 -> 127 - 21 -> 33

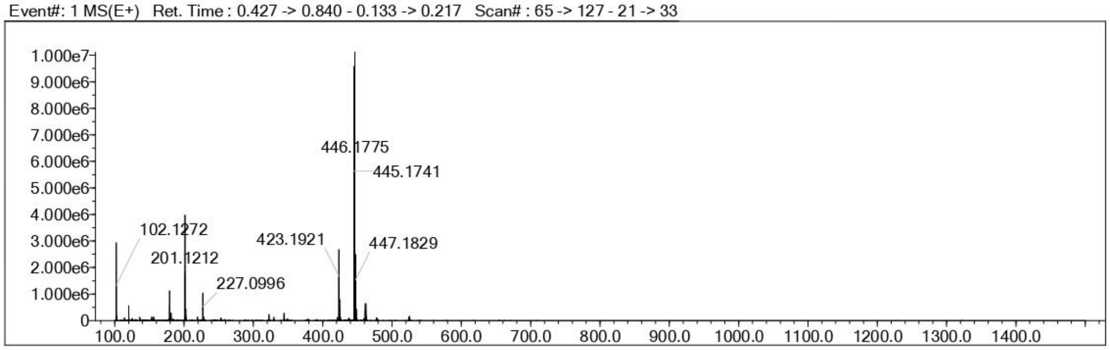

Measured region for 423.1921 m/z

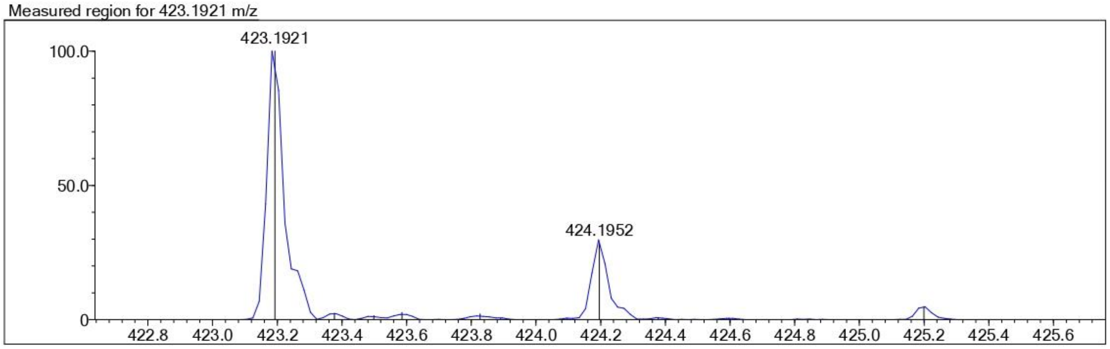

C24 H26 N2 O5 [M+H]+ : Predicted region for 423.1914 m/z

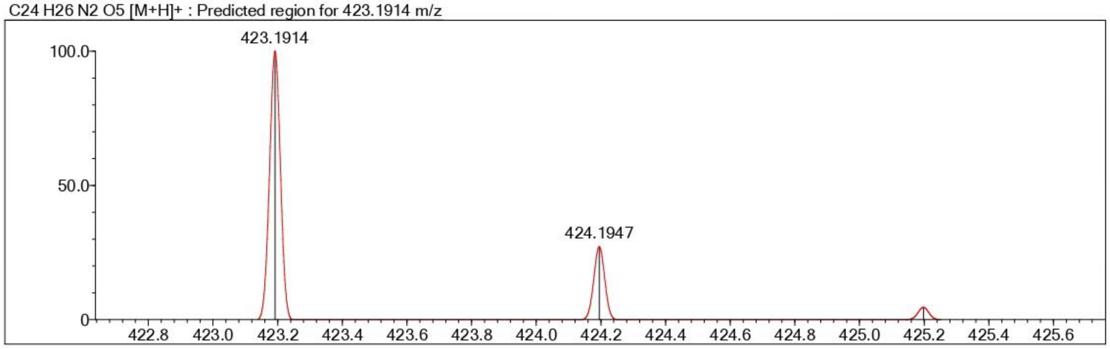

Rank	Score	Formula (M)	Ion	Meas. m/z	Pred. m/z	Df. (mDa)	Df. (ppm)	Iso	DBE
1	69.29	C24 H26 N2 O5	[M+H]+	423.1921	423.1914	0.7	1.65	70.44	13.0

^{1}H NMR of **10** (500 MHz, $CDCl_3$)

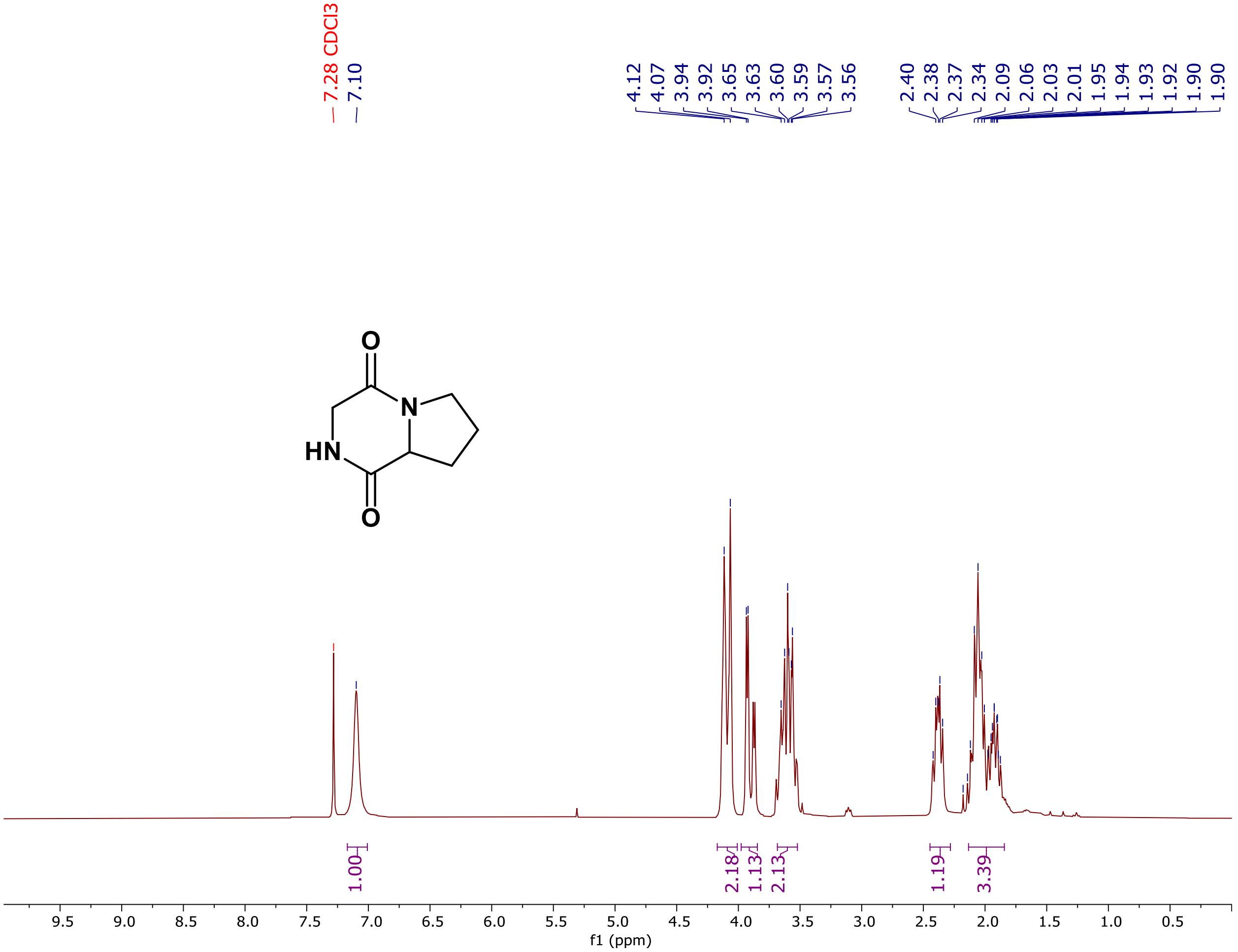

^{13}C NMR of **10** (500 MHz, $CDCl_3$)

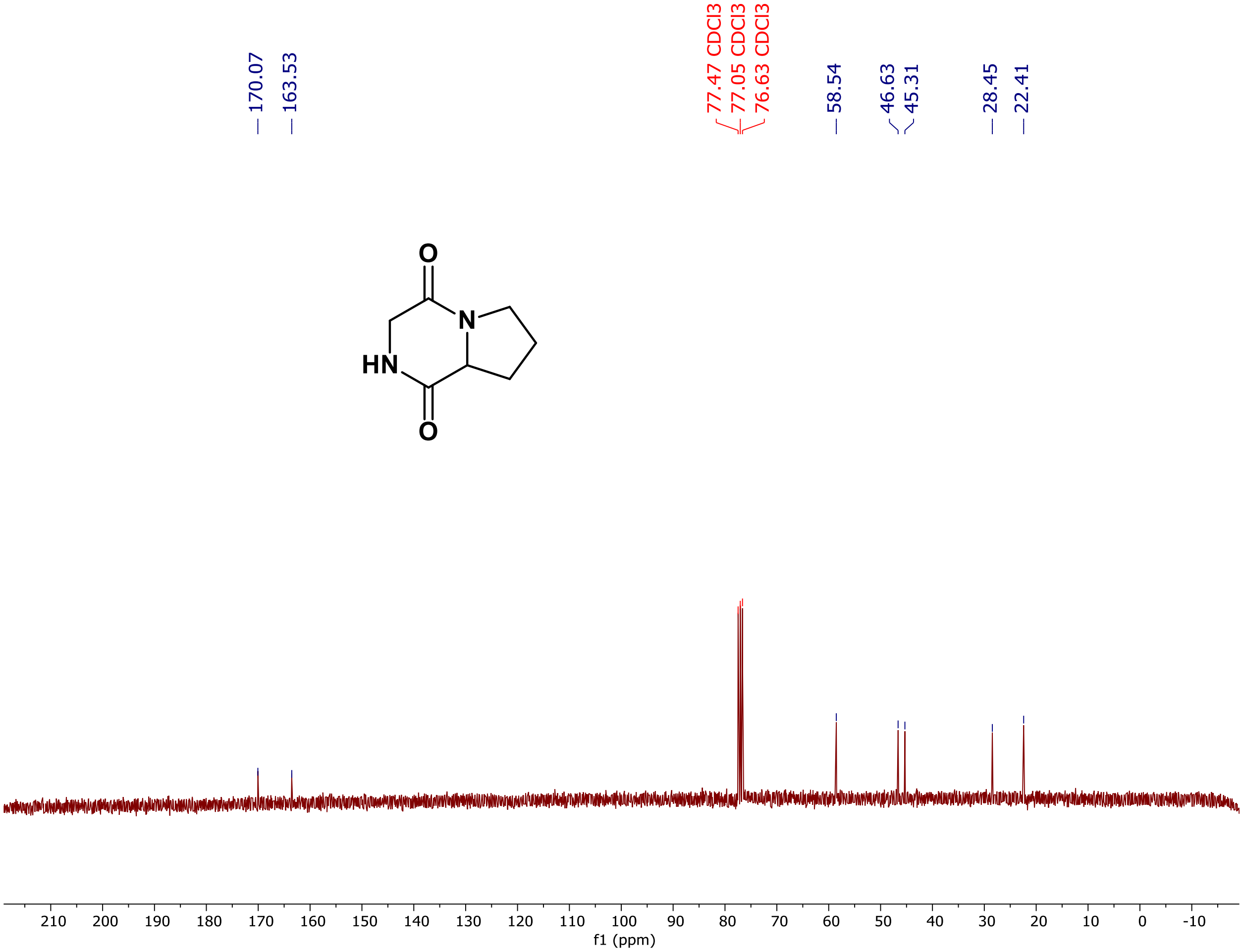

HRMS of **10**

Formula Predictor Report - CPD 2 diketo 154_06042021 Analysis HRMS_48.lcd Page 1 of 1

Data File: C:\LabSolutions\Data\Vilashini Rajaratnam\06042021 Analysis\CPD 2 diketo 154_06042021 Analysis HRMS_48.lcd

Elmt	Val.	Min	Max	Elmt	Val.	Min	Max	Elmt	Val.	Min	Max	Elmt	Val.	Min	Max	Use Adduct
H	1	5	20	O	2	0	3	P	3	0	0	I	3	0	0	H
2H	1	0	0	F	1	0	0	S	2	0	0					Na
C	4	5	15	Na	1	0	0	Cl	1	0	0					K
N	3	0	3	Si	4	0	0	Br	1	0	0					NH4

Error Margin (ppm): 500
HC Ratio: unlimited
Max Isotopes: all
MSn Iso RI (%): 75.00
DBE Range: -100.0 - 2000.0
Apply N Rule: no
Isotope RI (%): 1.00
MSn Logic Mode: AND
Electron Ions: both
Use MSn Info: yes
Isotope Res: 10000
Max Results: 10

Event#: 1 MS(E+) Ret. Time : 0.480 -> 0.693 - 0.053 -> 0.133 Scan# : 73 -> 105 - 9 -> 21

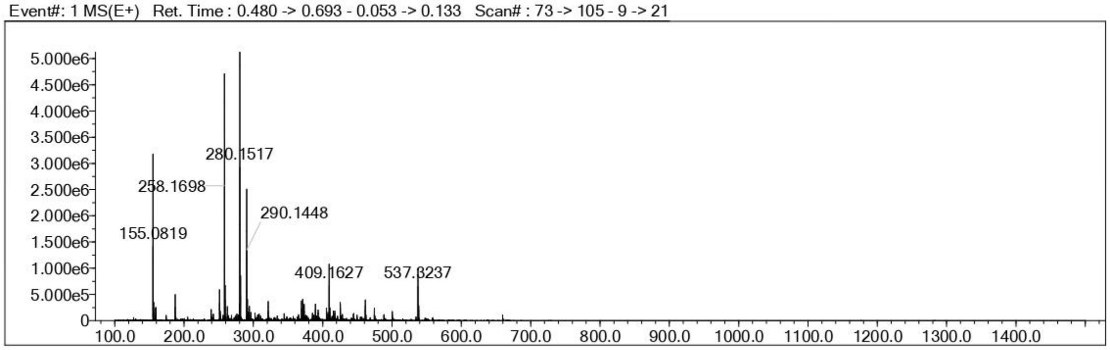

Measured region for 155.0819 m/z

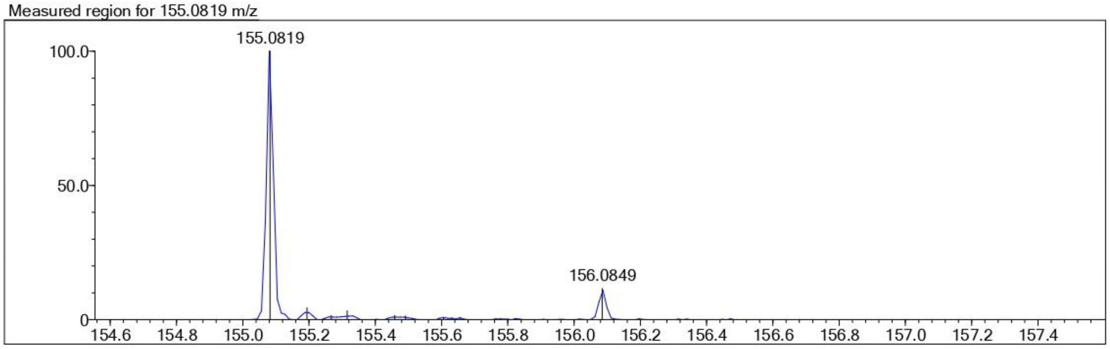

C7 H10 N2 O2 [M+H]+ : Predicted region for 155.0815 m/z

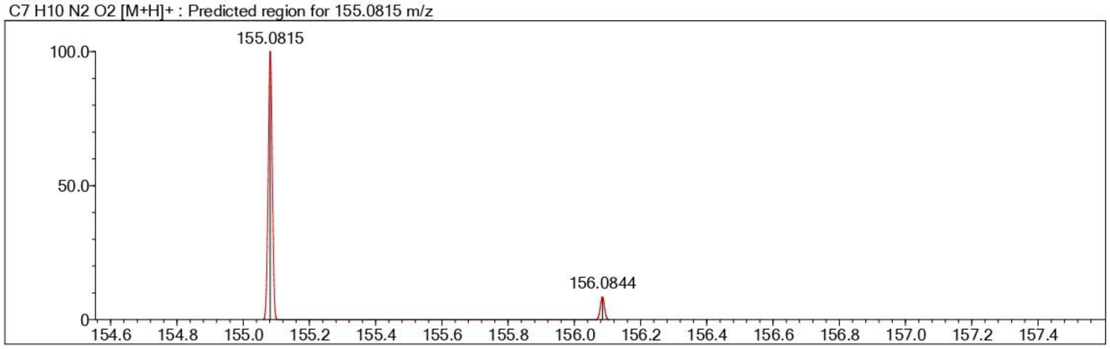

Rank	Score	Formula (M)	Ion	Meas. m/z	Pred. m/z	Df. (mDa)	Df. (ppm)	Iso	DBE
1	71.74	C7 H10 N2 O2	[M+H]+	155.0819	155.0815	0.4	2.58	74.69	4.0

^{1}H NMR of **5** (500 MHz, $CDCl_3$)

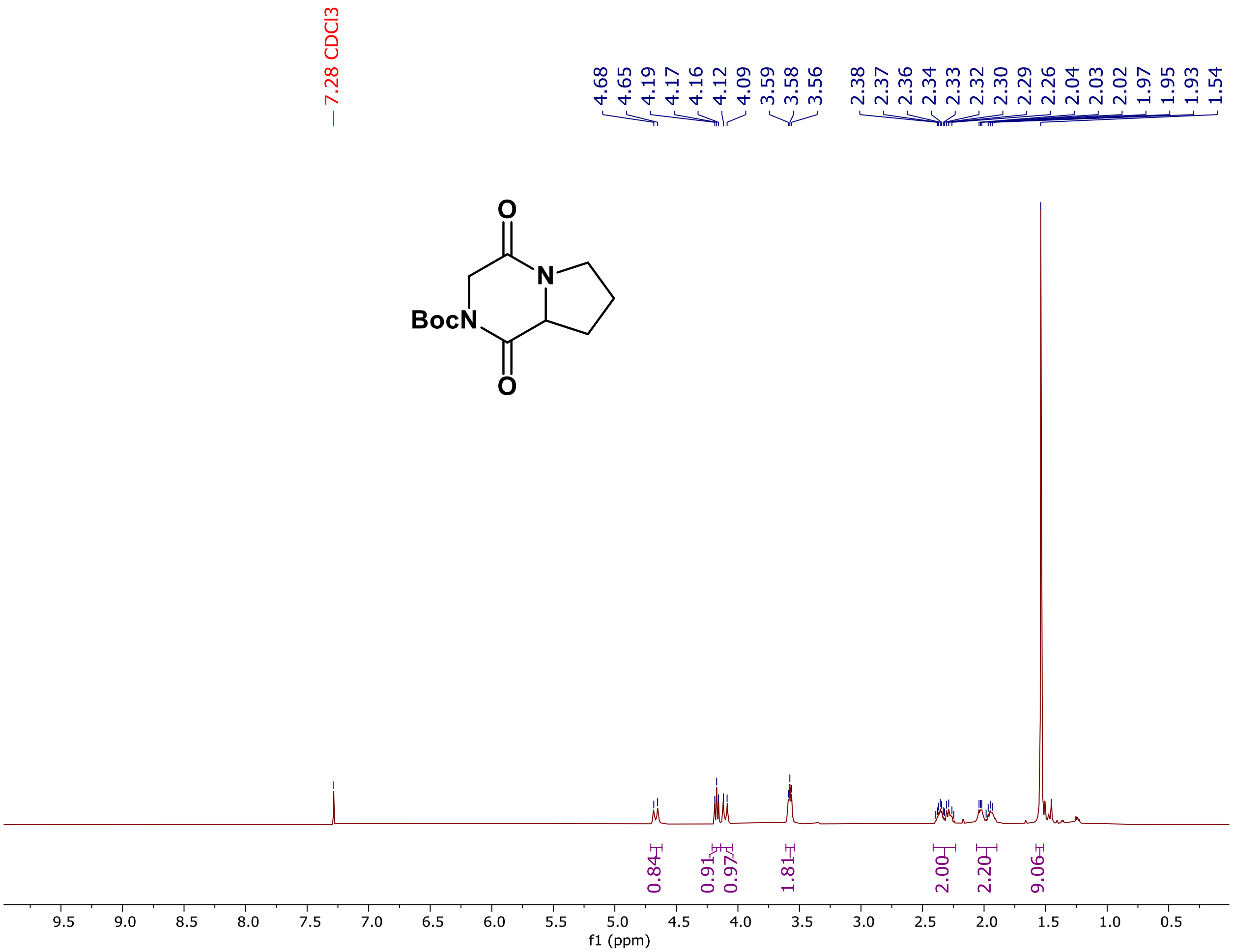

^{13}C NMR of **5** (500 MHz, $CDCl_3$)

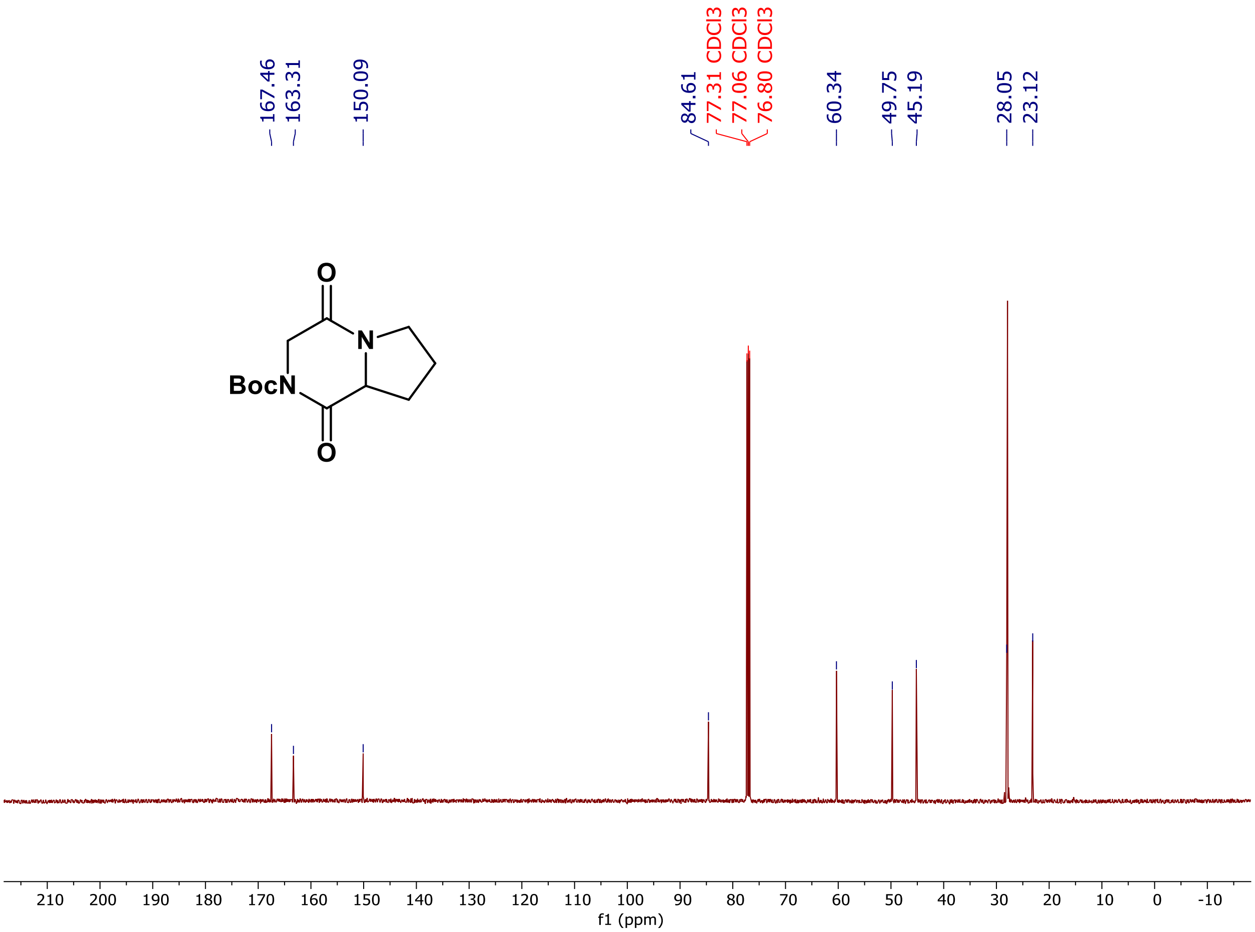

HRMS of **5**

Formula Predictor Report - CPD 3 Bocdiketo_06042021 Analysis HRMS_38.lcd Page 1 of 1

Data File: C:\LabSolutions\Data\Vilashini Rajaratnam\06042021 Analysis\CPD 3 Bocdiketo_06042021 Analysis HRMS_38.lcd

Elmt	Val.	Min	Max	Elmt	Val.	Min	Max	Elmt	Val.	Min	Max	Elmt	Val.	Min	Max	Use Adduct
H	1	15	35	O	2	2	6	P	3	0	0	I	3	0	0	H
2H	1	0	0	F	1	0	0	S	2	0	0					Na
C	4	10	30	Na	1	0	0	Cl	1	0	0					K
N	3	2	5	Si	4	0	0	Br	1	0	0					NH4

Error Margin (ppm): 500
HC Ratio: unlimited
Max Isotopes: all
MSn Iso RI (%): 75.00
DBE Range: -100.0 - 2000.0
Apply N Rule: no
Isotope RI (%): 1.00
MSn Logic Mode: AND
Electron Ions: both
Use MSn Info: yes
Isotope Res: 10000
Max Results: 10

Event#: 1 MS(E+) Ret. Time : 0.427 -> 0.507 - 0.107 -> 0.206 Scan# : 65 -> 77 - 17 -> 31

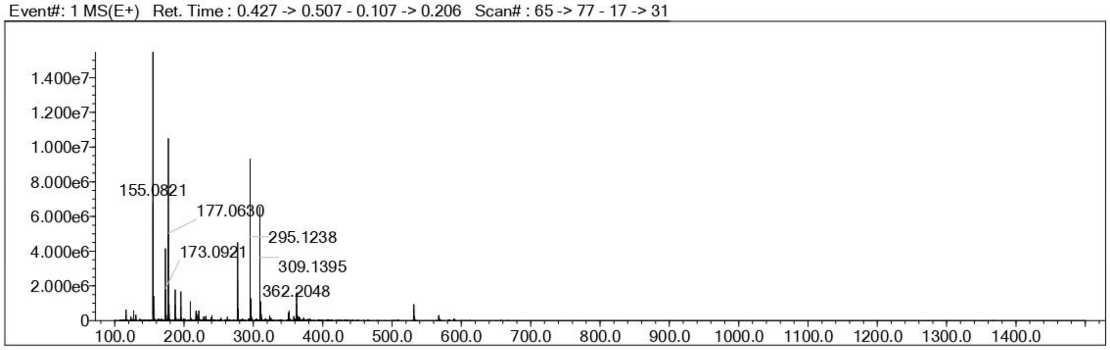

Measured region for 277.1136 m/z

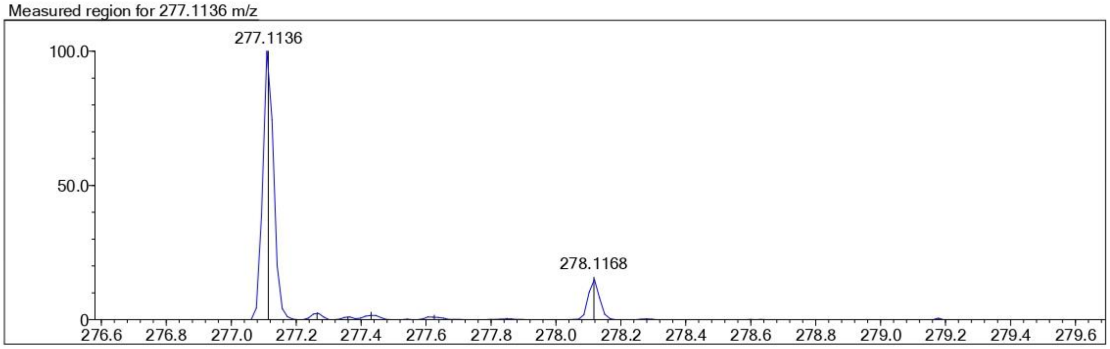

C12 H18 N2 O4 [M+Na]+ : Predicted region for 277.1159 m/z

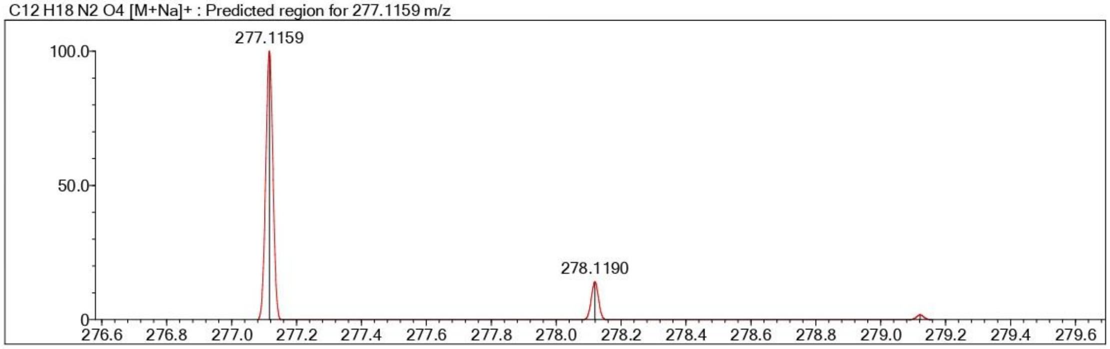

Rank	Score	Formula (M)	Ion	Meas. m/z	Pred. m/z	Df. (mDa)	Df. (ppm)	Iso	DBE
2	33.92	C12 H18 N2 O4	[M+Na]+	277.1136	277.1159	-2.3	-8.30	59.50	5.0

^{1}H NMR of **12** (500 MHz, $CDCl_3$)

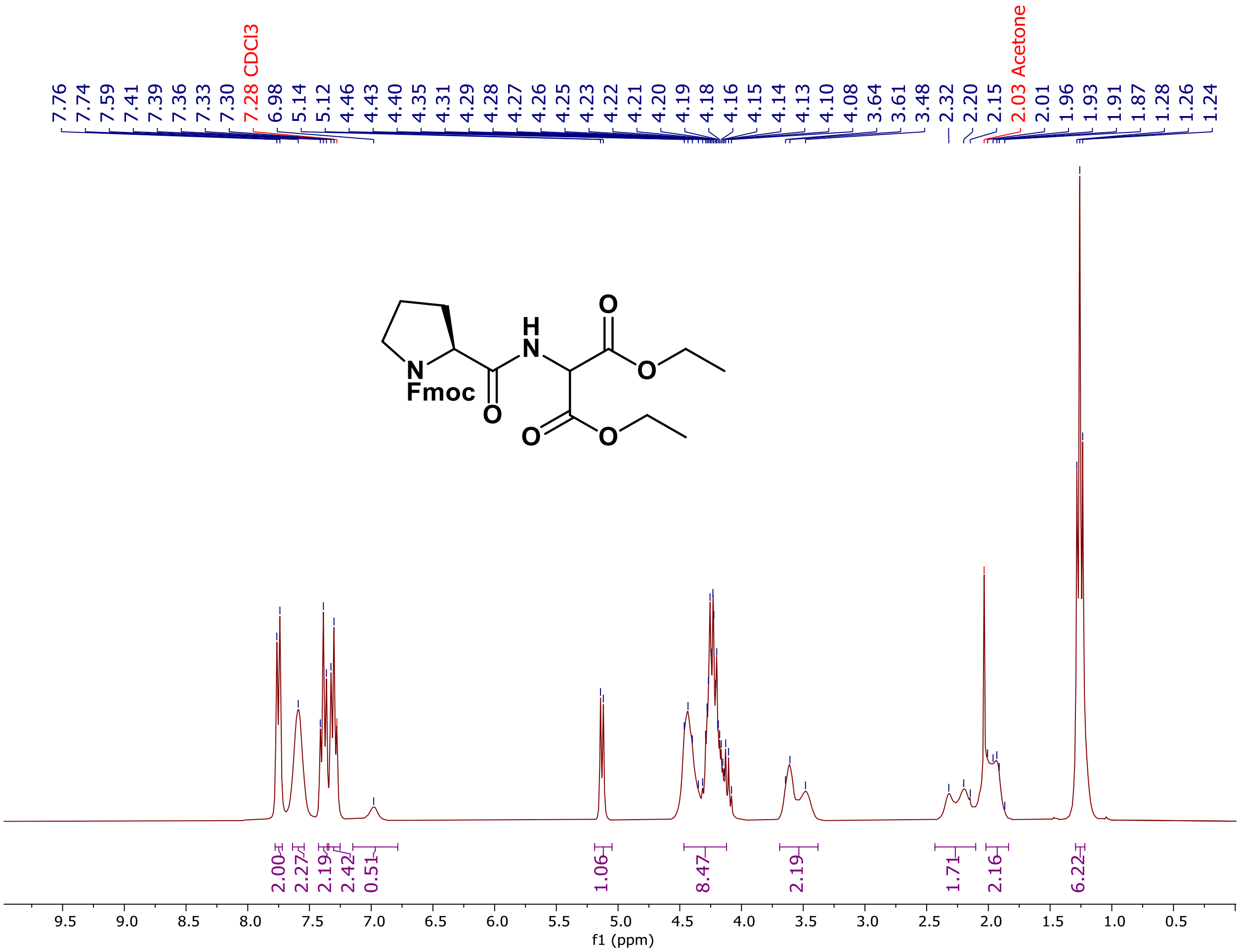

^{13}C NMR of **12** (500 MHz, $CDCl_3$)

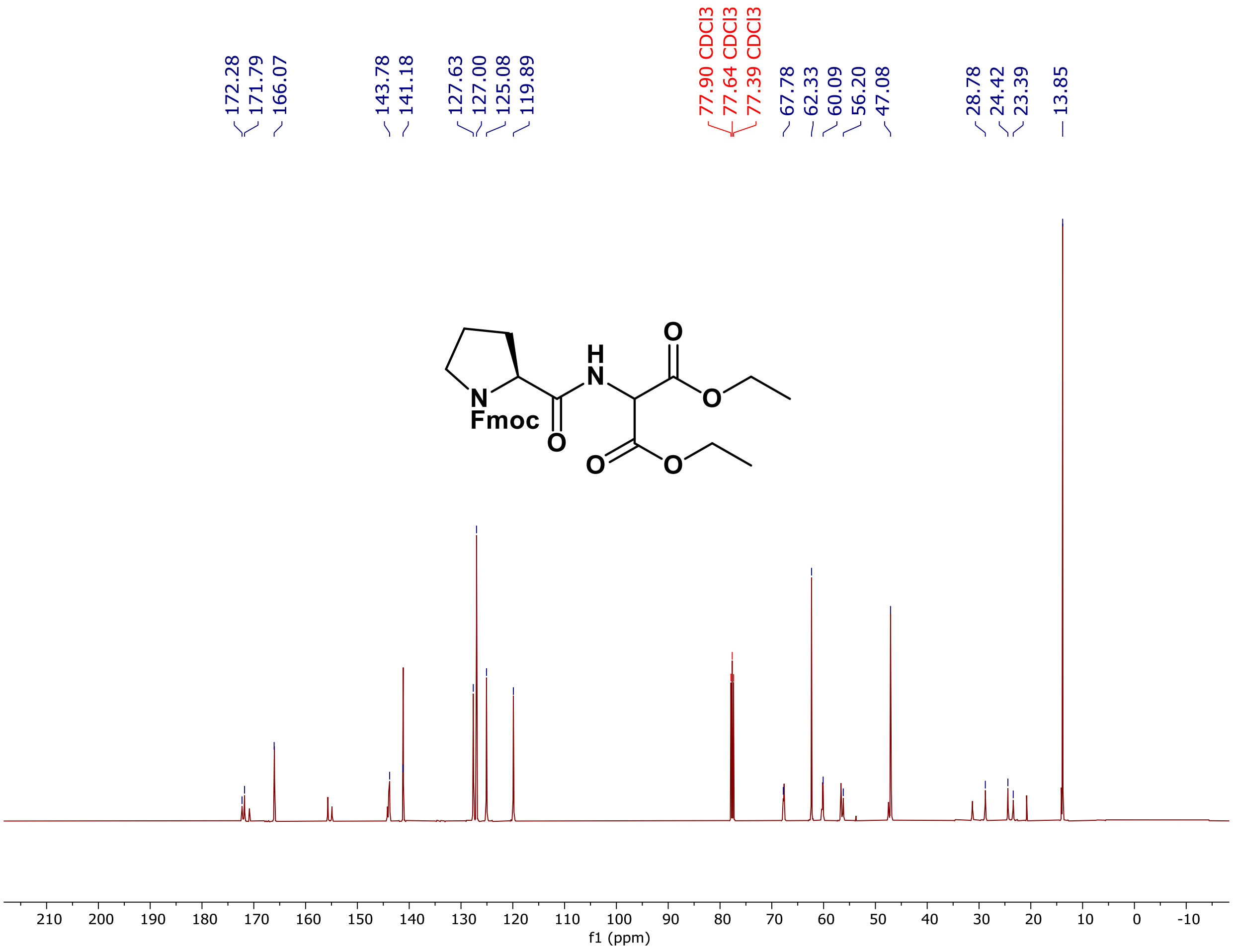

HRMS of **12**

Formula Predictor Report - CPD 1-_06042021 Analysis HRMS_40.lcd | Page 1 of 1

Data File: C:\LabSolutions\Data\Vilashini Rajaratnam\06042021 Analysis\CPD 1-_06042021 Analysis HRMS_40.lcd

Elmt	Val.	Min	Max	Elmt	Val.	Min	Max	Elmt	Val.	Min	Max	Elmt	Val.	Min	Max	Use Adduct
H	1	25	35	O	2	5	9	P	3	0	0	I	3	0	0	H
2H	1	0	0	F	1	0	0	S	2	0	0					Na
C	4	20	35	Na	1	0	0	Cl	1	0	0					K
N	3	0	3	Si	4	0	0	Br	1	0	0					NH4

Error Margin (ppm):	500	DBE Range:	-100.0 - 2000.0	Electron Ions:	both
HC Ratio:	unlimited	Apply N Rule:	no	Use MSn Info:	yes
Max Isotopes:	all	Isotope RI (%):	1.00	Isotope Res:	10000
MSn Iso RI (%):	75.00	MSn Logic Mode:	AND	Max Results:	10

Event#: 1 MS(E+) Ret. Time : 0.427 -> 0.893 Scan# : 65 -> 135

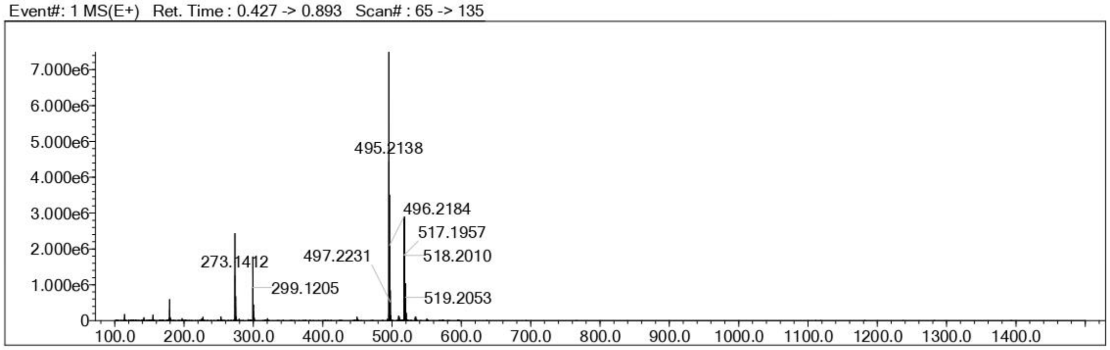

Measured region for 495.2138 m/z

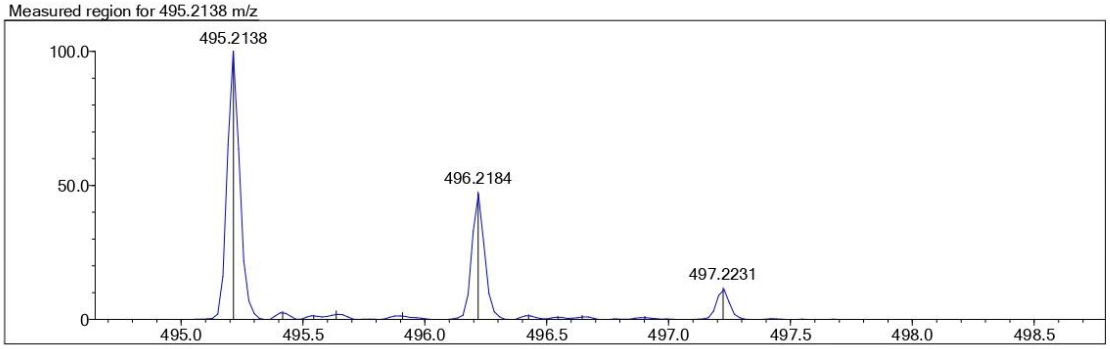

C27 H30 N2 O7 [M+H]+ : Predicted region for 495.2126 m/z

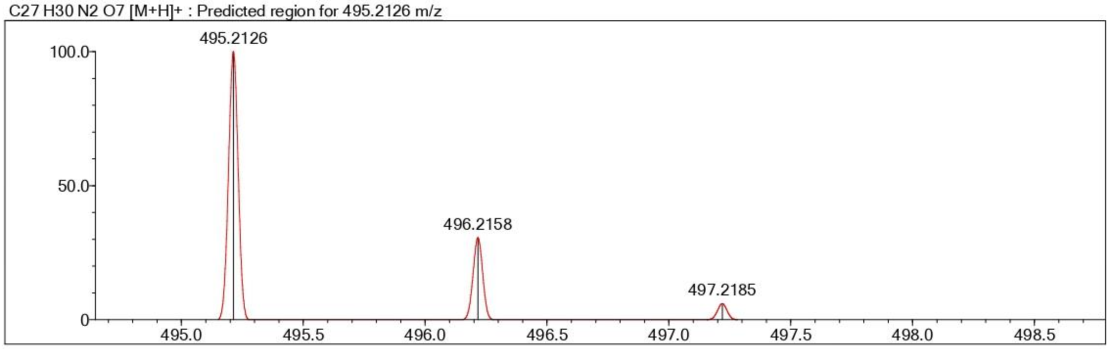

Rank	Score	Formula (M)	Ion	Meas. m/z	Pred. m/z	Df. (mDa)	Df. (ppm)	Iso	DBE
2	66.19	C27 H30 N2 O7	[M+H]+	495.2138	495.2126	1.2	2.42	68.63	14.0

^{1}H NMR of **6** (500 MHz, $CDCl_3$) ester DKP

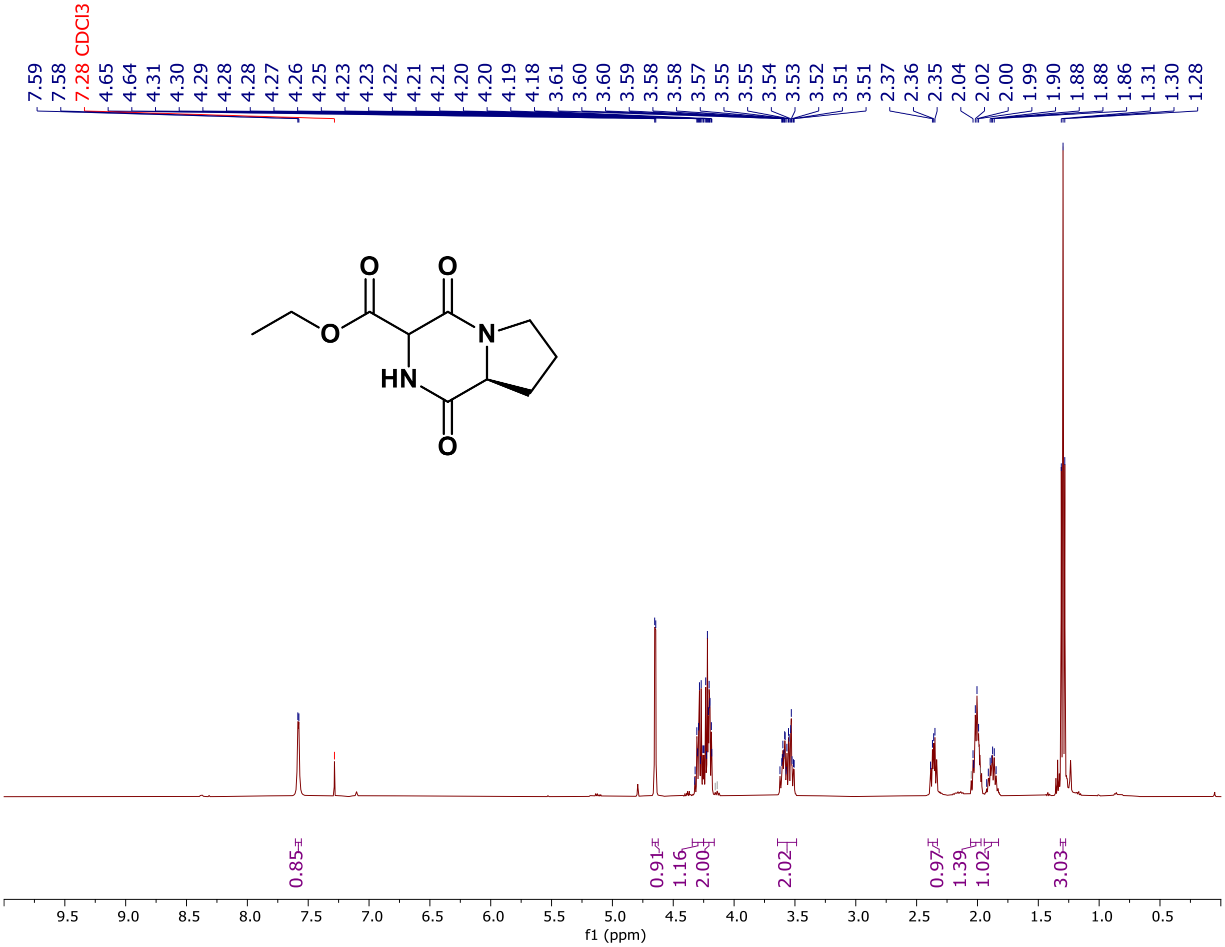

^{13}C NMR of **6** (500 MHz, $CDCl_3$)

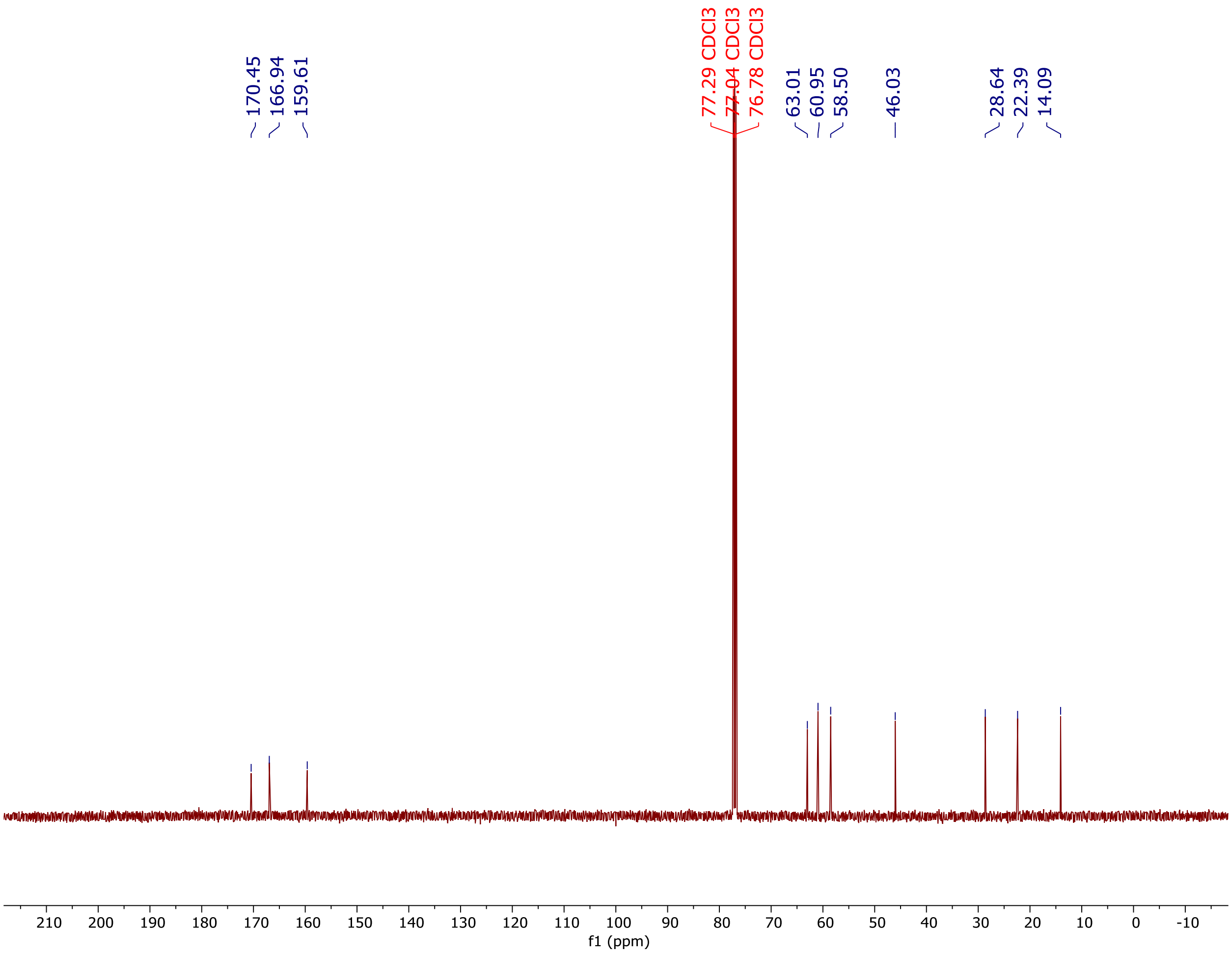

HRMS of **6**

Formula Predictor Report - CPD 2' ester diketo_06042021 Analysis HRMS_52.lcd

Data File: C:\LabSolutions\Data\Vilashini Rajaratnam\06042021 Analysis\CPD 2' ester diketo_06042021 Analysis HRMS_52.lcd

Elmt	Val.	Min	Max	Elmt	Val.	Min	Max	Elmt	Val.	Min	Max	Elmt	Val.	Min	Max	Use Adduct
H	1	5	20	O	2	0	4	P	3	0	0	I	3	0	0	H
2H	1	0	0	F	1	0	0	S	2	0	0					HCOO
C	4	5	15	Na	1	0	0	Cl	1	0	0					CH3COO
N	3	0	3	Si	4	0	0	Br	1	0	0					Cl

Error Margin (ppm):	500	DBE Range:	-100.0 - 2000.0	Electron Ions:	both
HC Ratio:	unlimited	Apply N Rule:	no	Use MSn Info:	yes
Max Isotopes:	all	Isotope RI (%):	1.00	Isotope Res:	10000
MSn Iso RI (%):	75.00	MSn Logic Mode:	AND	Max Results:	10

Event#: 2 MS(E-) Ret. Time : 0.440 -> 0.733 - 0.133 -> 0.252 Scan# : 68 -> 112 - 22 -> 40

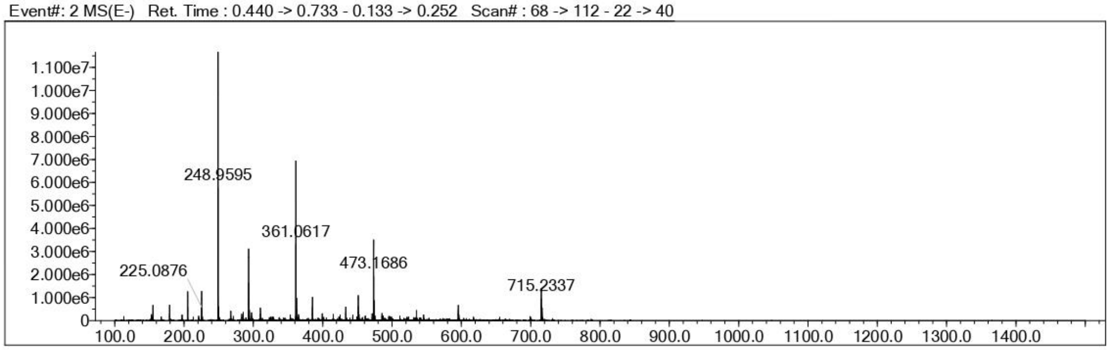

Measured region for 225.0876 m/z

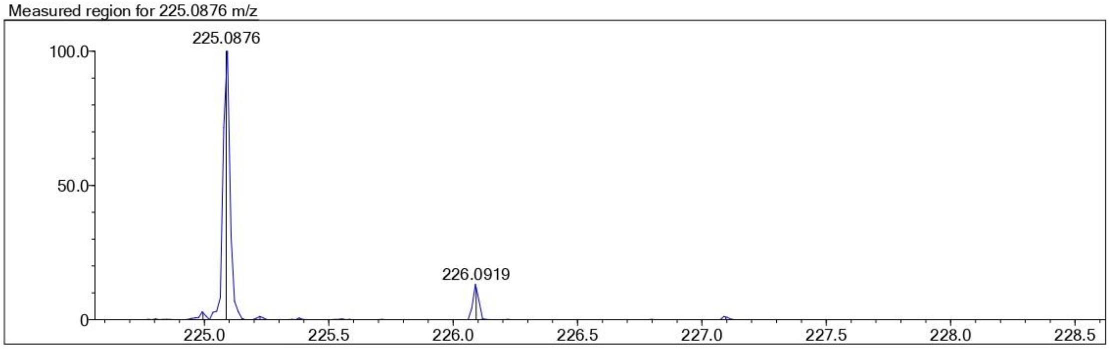

C10 H14 N2 O4 [M-H]- : Predicted region for 225.0881 m/z

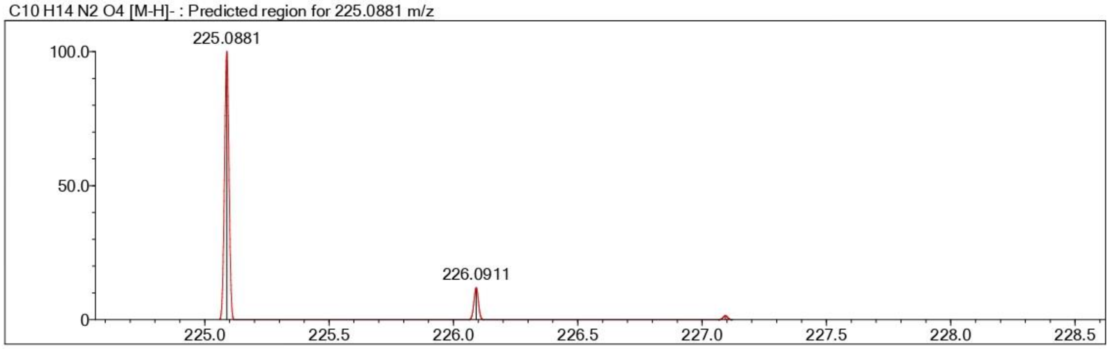

Rank	Score	Formula (M)	Ion	Meas. m/z	Pred. m/z	Df. (mDa)	Df. (ppm)	Iso	DBE
1	70.77	C10 H14 N2 O4	[M-H]-	225.0876	225.0881	-0.5	-2.22	73.00	5.0

^{1}H NMR of **13** (500 MHz, $CDCl_3$)

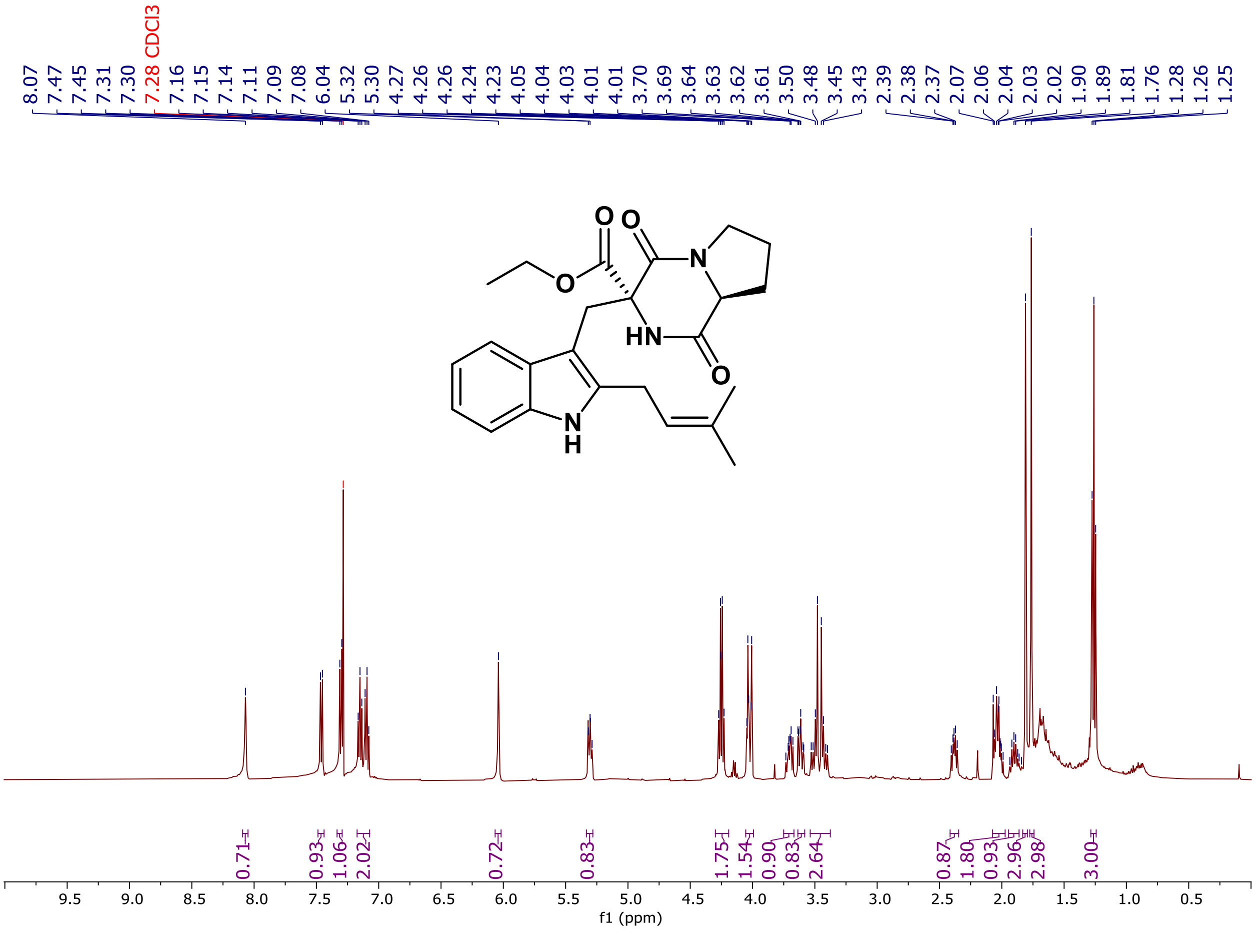

^{13}C NMR of **13** (500 MHz, $CDCl_3$)

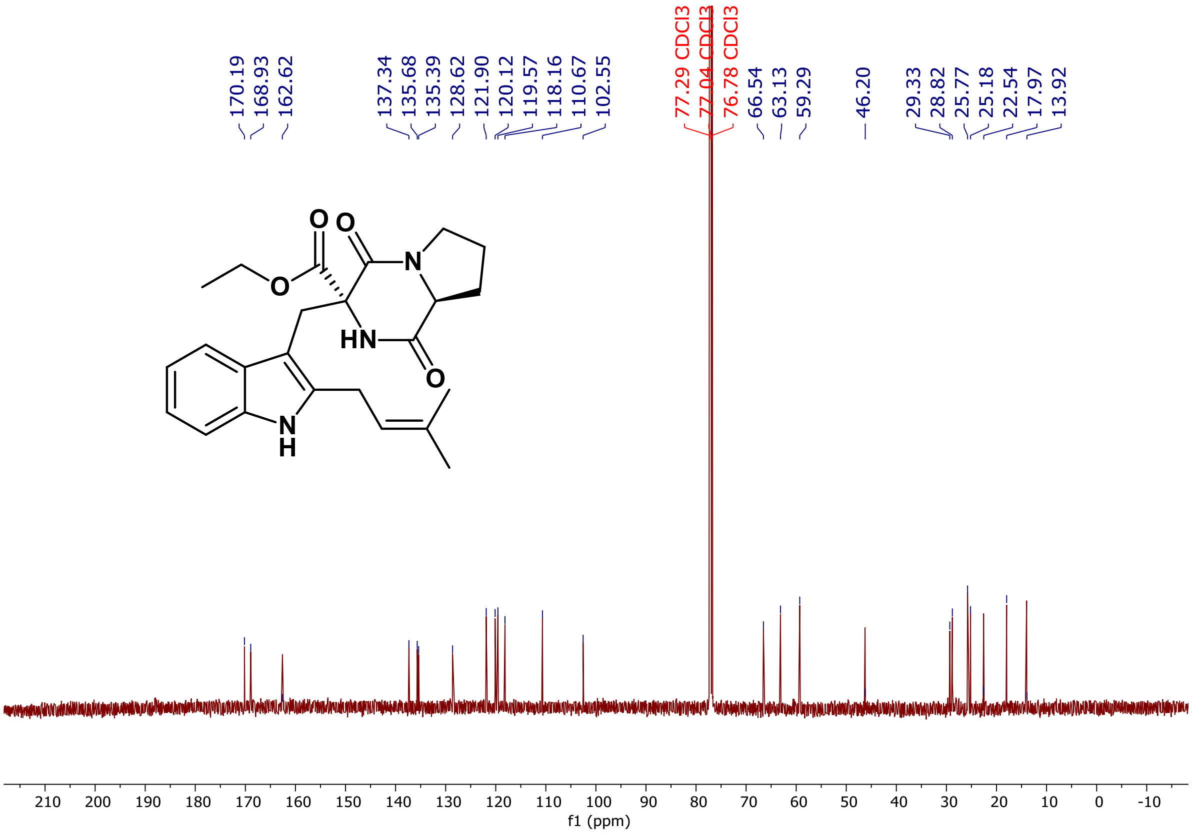

HRMS of **13**

Formula Predictor Report - Polar TPS-B more_06042021 Analysis HRMS_30.lcd

Page 1 of 1

Data File: C:\LabSolutions\Data\Vilashini Rajaratnam\06042021 Analysis\Polar TPS-B more_06042021 Analysis HRMS_30.lcd

Elmt	Val.	Min	Max	Elmt	Val.	Min	Max	Elmt	Val.	Min	Max	Elmt	Val.	Min	Max	Use Adduct
H	1	25	35	O	2	2	6	P	3	0	0	I	3	0	0	H
2H	1	0	0	F	1	0	0	S	2	0	0					Na
C	4	20	30	Na	1	0	0	Cl	1	0	0					K
N	3	2	5	Si	4	0	0	Br	1	0	0					NH4

Error Margin (ppm):	500	DBE Range:	-100.0 - 2000.0	Electron Ions:	both
HC Ratio:	unlimited	Apply N Rule:	no	Use MSn Info:	yes
Max Isotopes:	all	Isotope RI (%):	1.00	Isotope Res:	10000
MSn Iso RI (%):	75.00	MSn Logic Mode:	AND	Max Results:	10

Event#: 1 MS(E+) Ret. Time : 0.427 -> 0.907 - 0.080 -> 0.119 Scan# : 65 -> 137 - 13 -> 19

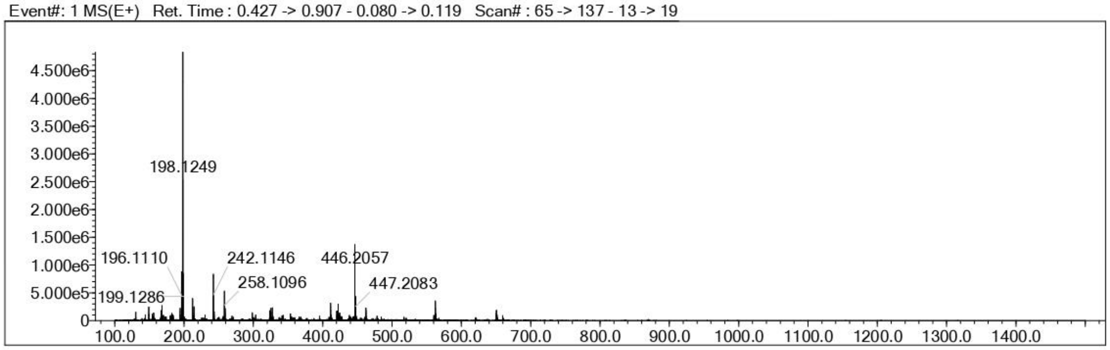

Measured region for 446.2057 m/z

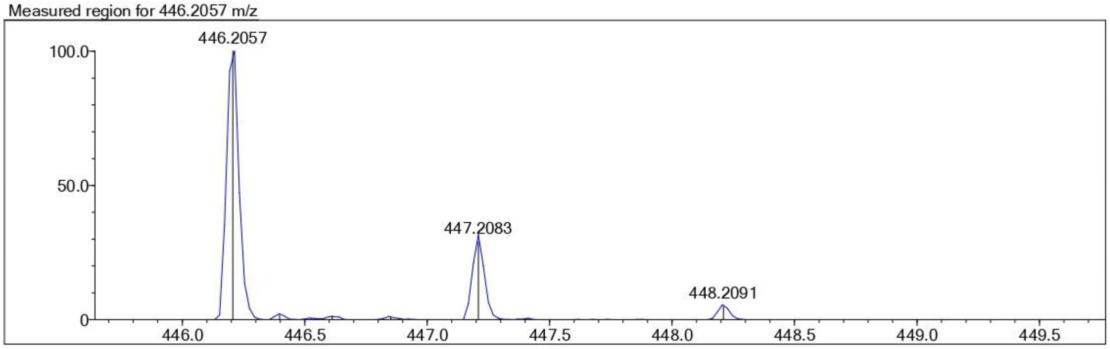

C24 H29 N3 O4 [M+Na]+ : Predicted region for 446.2050 m/z

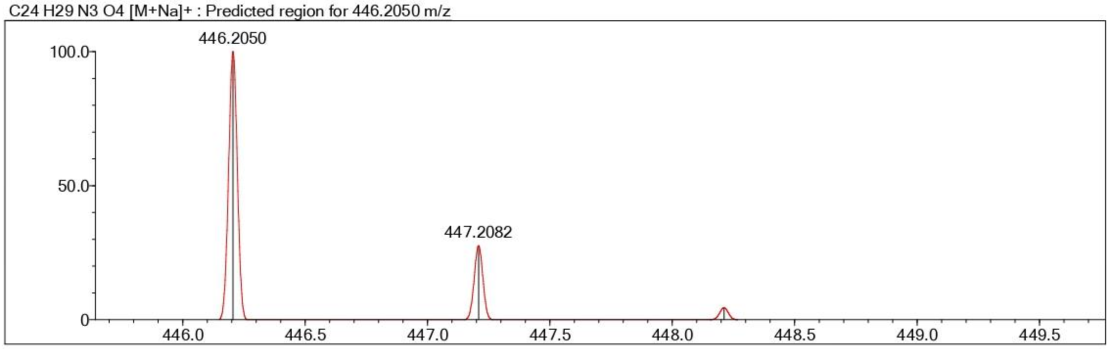

Rank	Score	Formula (M)	Ion	Meas. m/z	Pred. m/z	Df. (mDa)	Df. (ppm)	Iso	DBE
3	92.87	C24 H29 N3 O4	[M+Na]+	446.2057	446.2050	0.7	1.57	94.21	12.0

^{1}H NMR of **14** (500 MHz, $CDCl_3$)

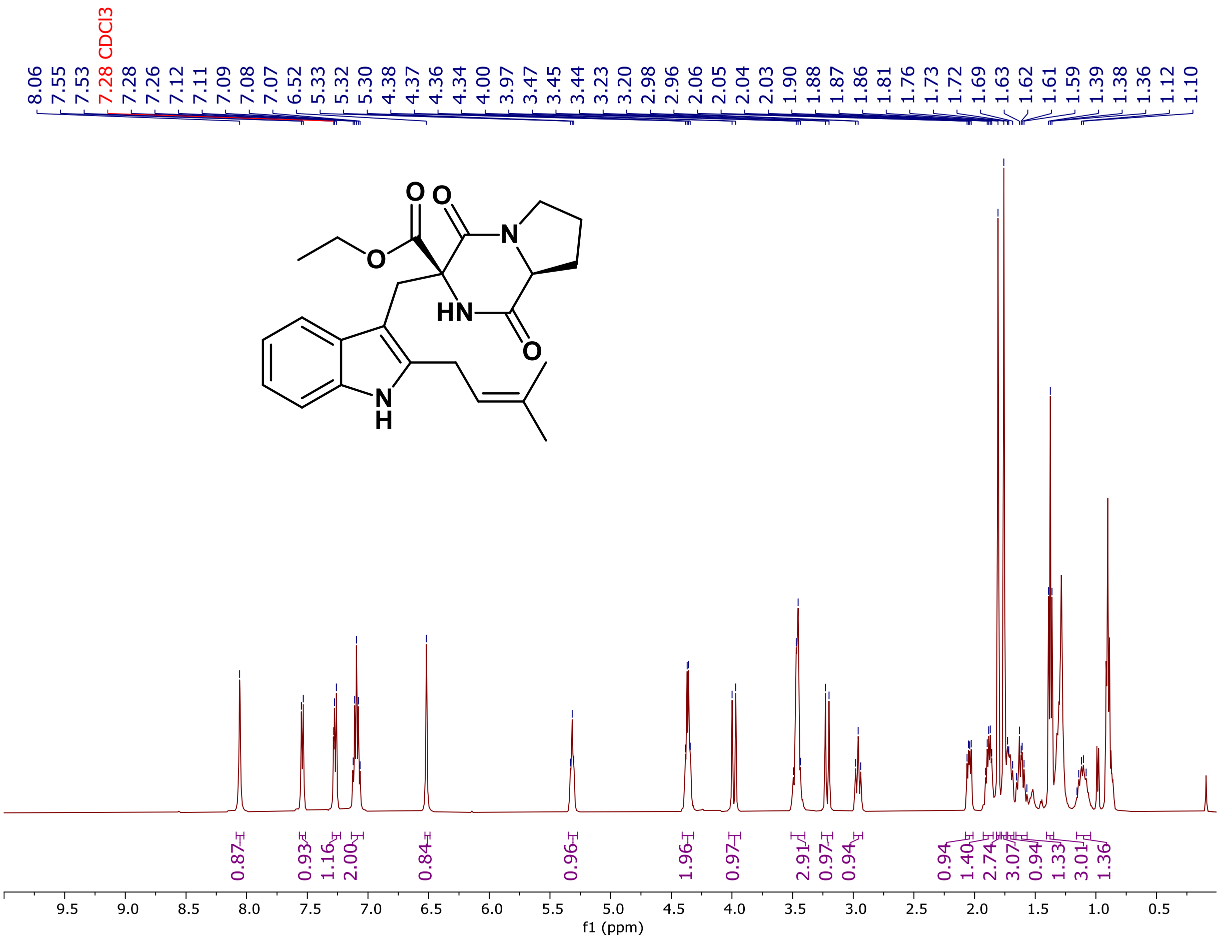

^{13}C NMR of **14** (500 MHz, $CDCl_3$)

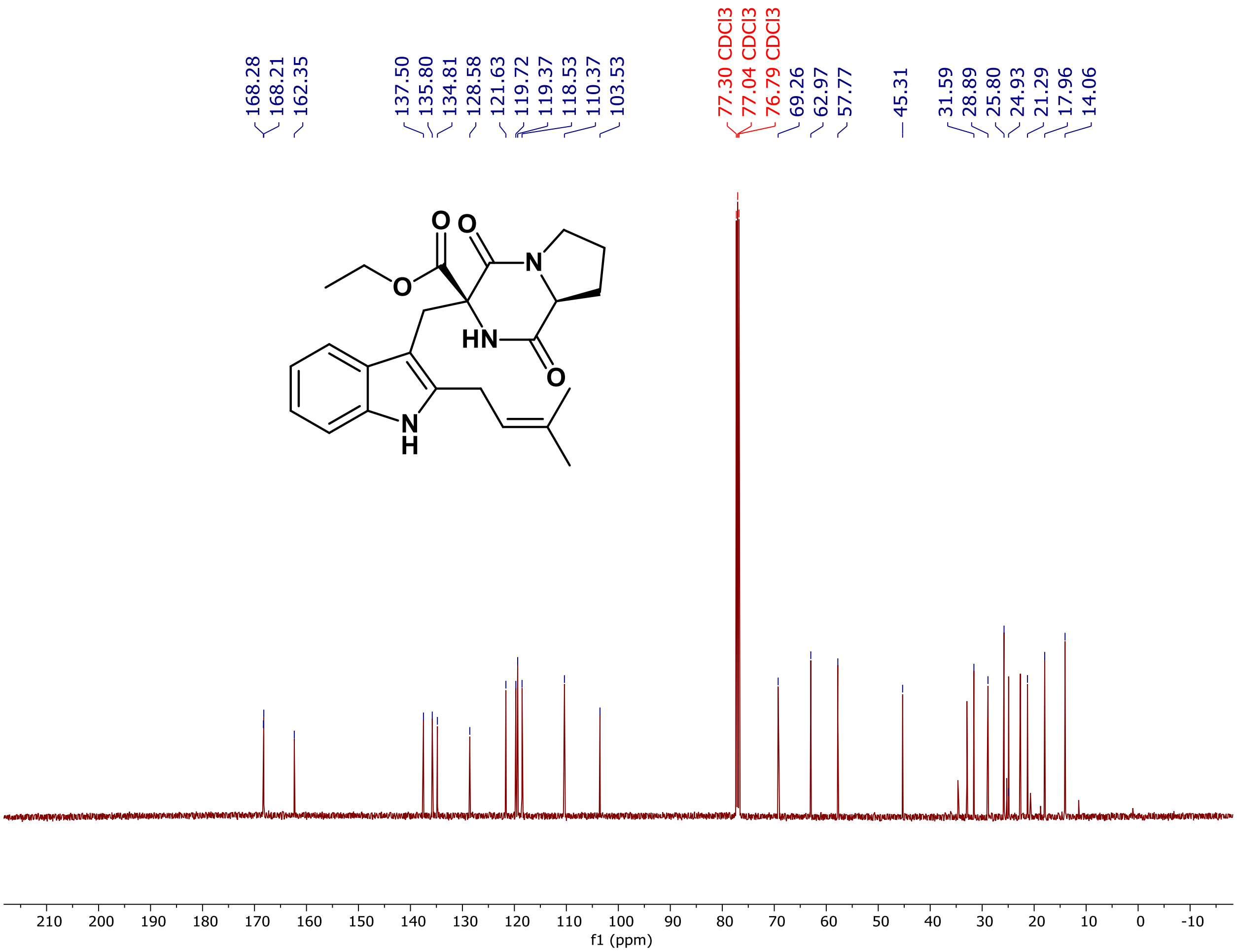

HRMS of **14**

Formula Predictor Result	**C24 H29 N3 O4**
Mass	424.22201
Error Margin	100 ppm
DBE Range	Not Used
Electron Ions	Both configurations
HC Ratio	Not Used
Nitrogen Rule	Not Used

Score	Pred. (M)	Pred. m/z	Meas. m/z	Diff. (mDa)	Formulae (M)	Ion	Diff. (ppm)	Iso Score	DBE
72.29	423.21581	424.22308	424.22201	-1.07	C24 H29 N3 O4	[M+H]+	-2.522	70.86	12.0

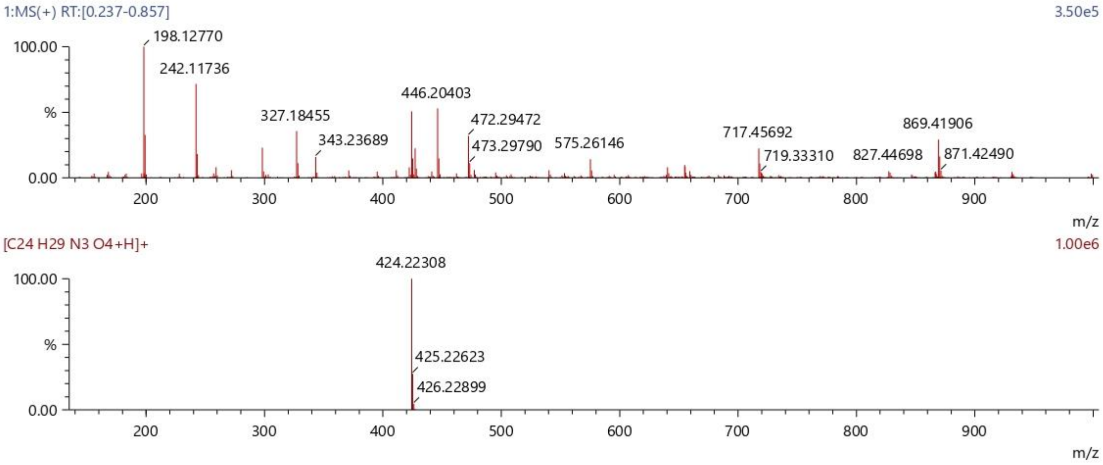

HSQC overlay of **13** and **14**

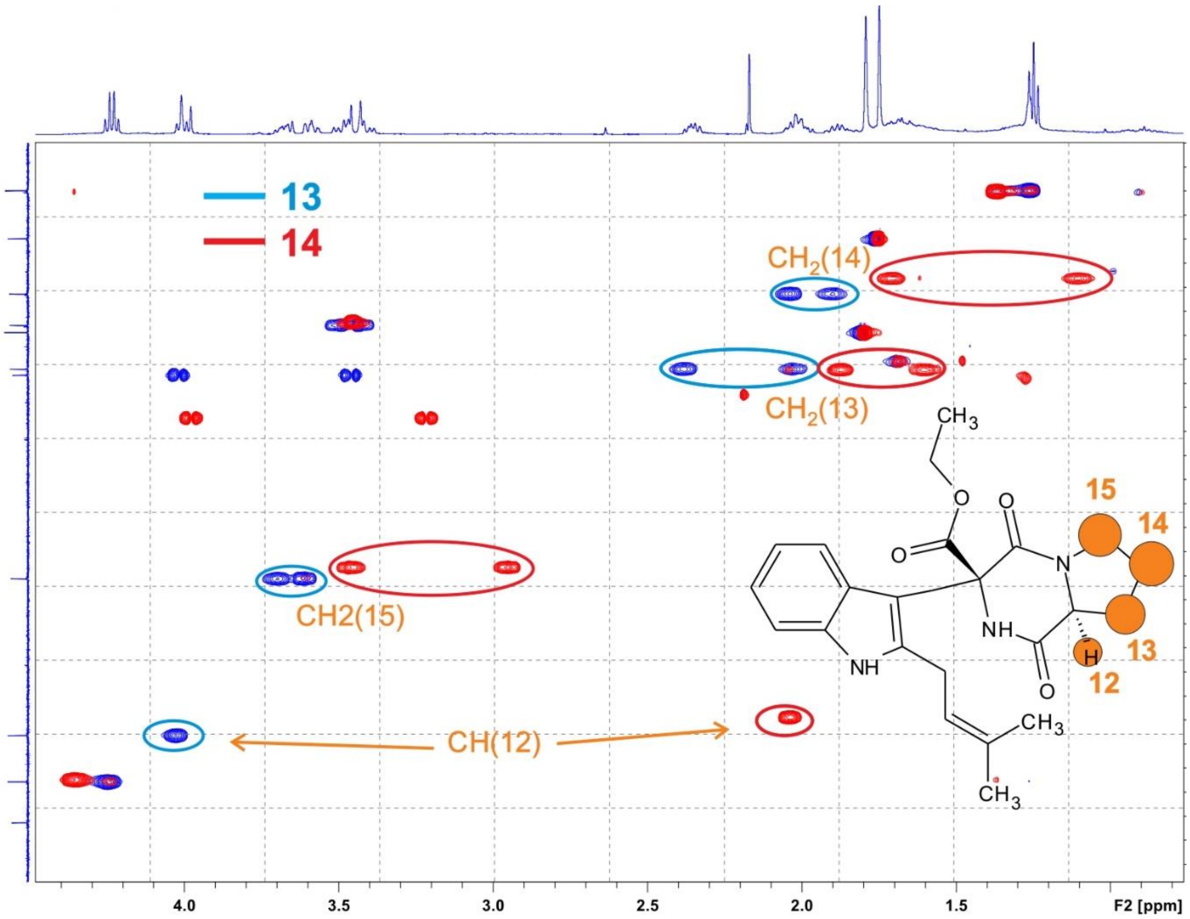

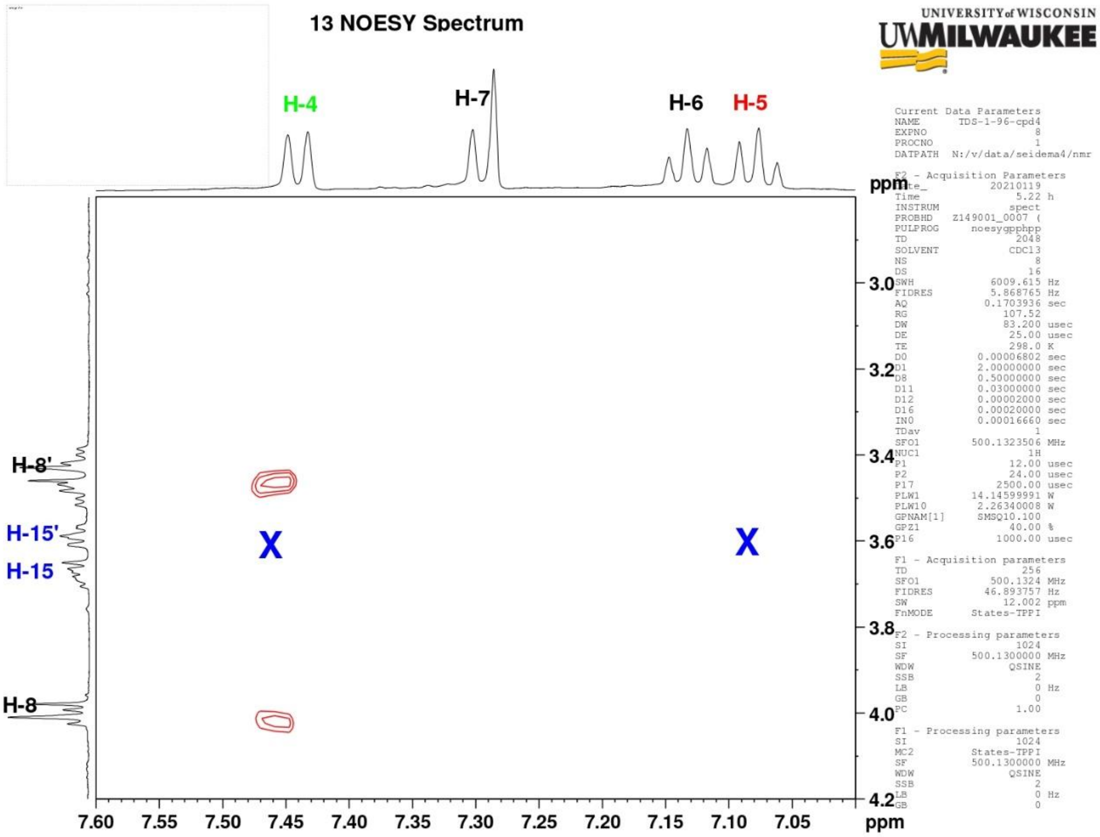
13 NOESY Spectrum
UNIVERSITY of WISCONSIN
UWMILWAUKEE
H-4
H-7
H-6
H-5
H-8'
H-15'
H-15
H-8
X
X
ppm
Current Data Parameters
NAME TDS-1-96-cpd4
EXPNO 8
PROCNO 1
DATPATH N:/v/data/seidema4/nmr
F2 - Acquisition Parameters
Time 5.22 h
INSTRUM spect
PROBHD Z149001_0007 (
PULPROG noesygpphpp
TD 2048
SOLVENT CDCl3
NS 8
DS 16
SWH 6009.615 Hz
FIDRES 5.868765 Hz
AQ 0.1703936 sec
RG 107.52
DW 83.200 usec
DE 25.00 usec
TE 298.0 K
D0 0.00006802 sec
D1 2.00000000 sec
D8 0.50000000 sec
D11 0.03000000 sec
D12 0.00002000 sec
D16 0.00020000 sec
IN0 0.00016660 sec
TDav 1
SFO1 500.1323506 MHz
NUC1 1H
P1 12.00 usec
P2 24.00 usec
P17 2500.00 usec
PLW1 14.14599991 W
PLW10 2.26340008 W
GPNAM[1] SMSQ10.100
GPZ1 40.00 %
P16 1000.00 usec
F1 - Acquisition parameters
TD 256
SFO1 500.1324 MHz
FIDRES 46.893757 Hz
SW 12.002 ppm
FnMODE States-TPPI
F2 - Processing parameters
SI 1024
SF 500.1300000 MHz
WDW QSINE
SSB 2
LB 0 Hz
GB 0
PC 1.00
F1 - Processing parameters
SI 1024
MC2 States-TPPI
SF 500.1300000 MHz
WDW QSINE
SSB 2
LB 0 Hz
GB 0
3.0
3.2
3.4
3.6
3.8
4.0
4.2
7.60 7.55 7.50 7.45 7.40 7.35 7.30 7.25 7.20 7.15 7.10 7.05

^{1}H NMR of **19** (500 MHz, $CDCl_3$)

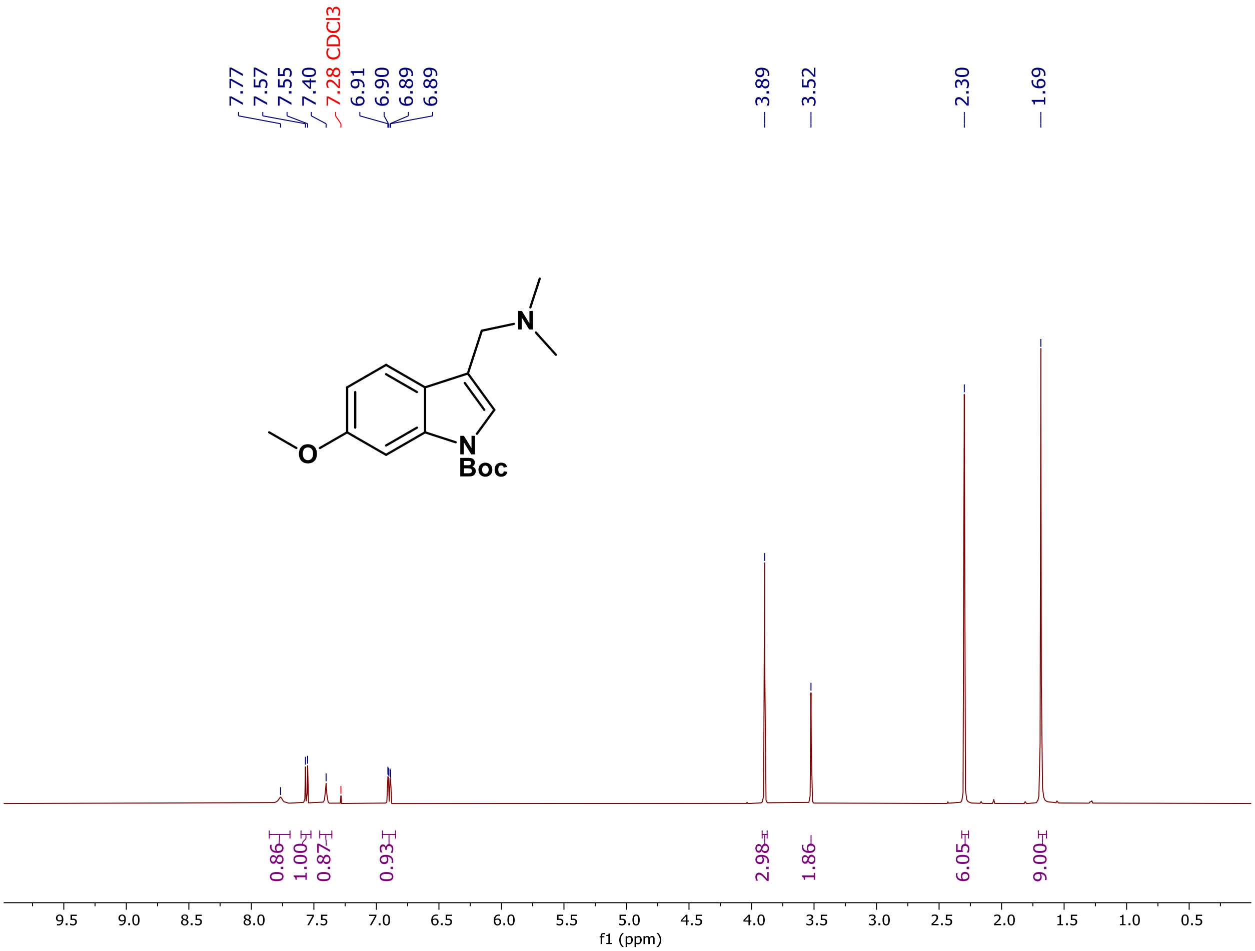

^{13}C NMR of **19** (500 MHz, $CDCl_3$)

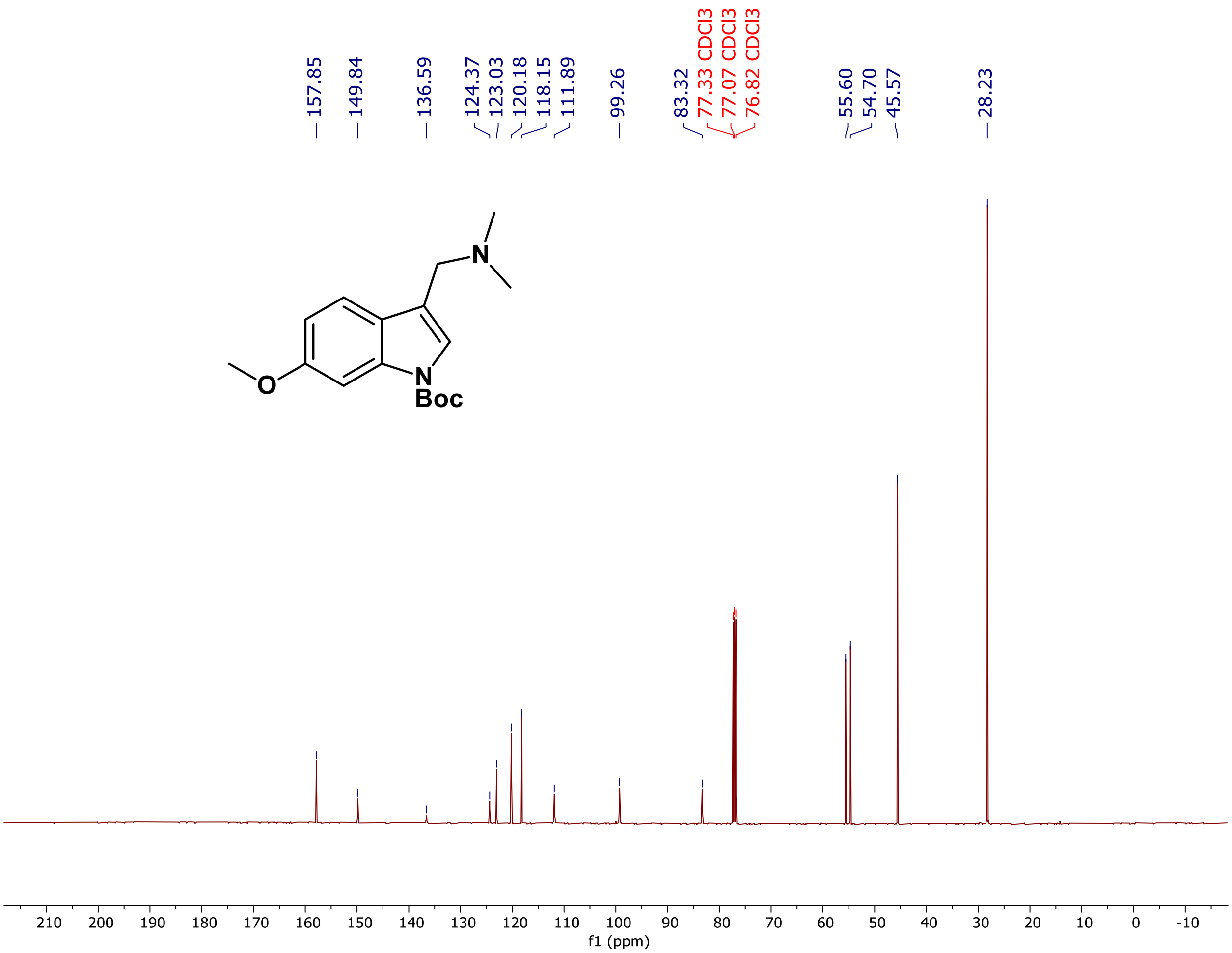

HRMS of **19**

5-23-2025.lcb

Formula Predictor Result	**C17 H24 N2 O3**
Mass	305.18603
Error Margin	100 ppm
DBE Range	Not Used
Electron Ions	Both configurations
HC Ratio	Not Used
Nitrogen Rule	Not Used

Score	Pred. (M)	Pred. m/z	Meas. m/z	Diff. (mDa)	Formulae (M)	Ion	Diff. (ppm)	Iso Score	DBE
69.20	304.17869	305.18597	305.18603	0.06	C17 H24 N2 O3	[M+H]+	0.197	65.85	7.0

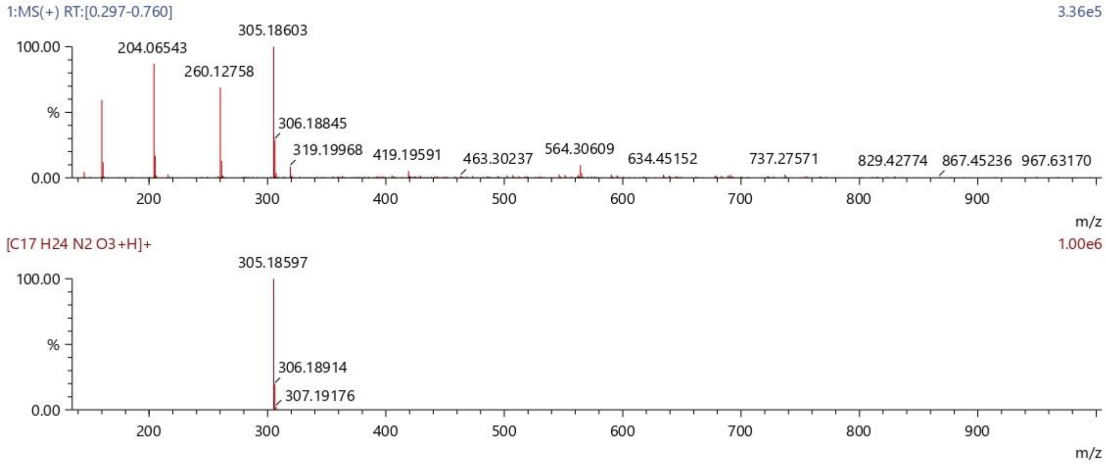

^{1}H NMR of **20** (500 MHz, $CDCl_3$)

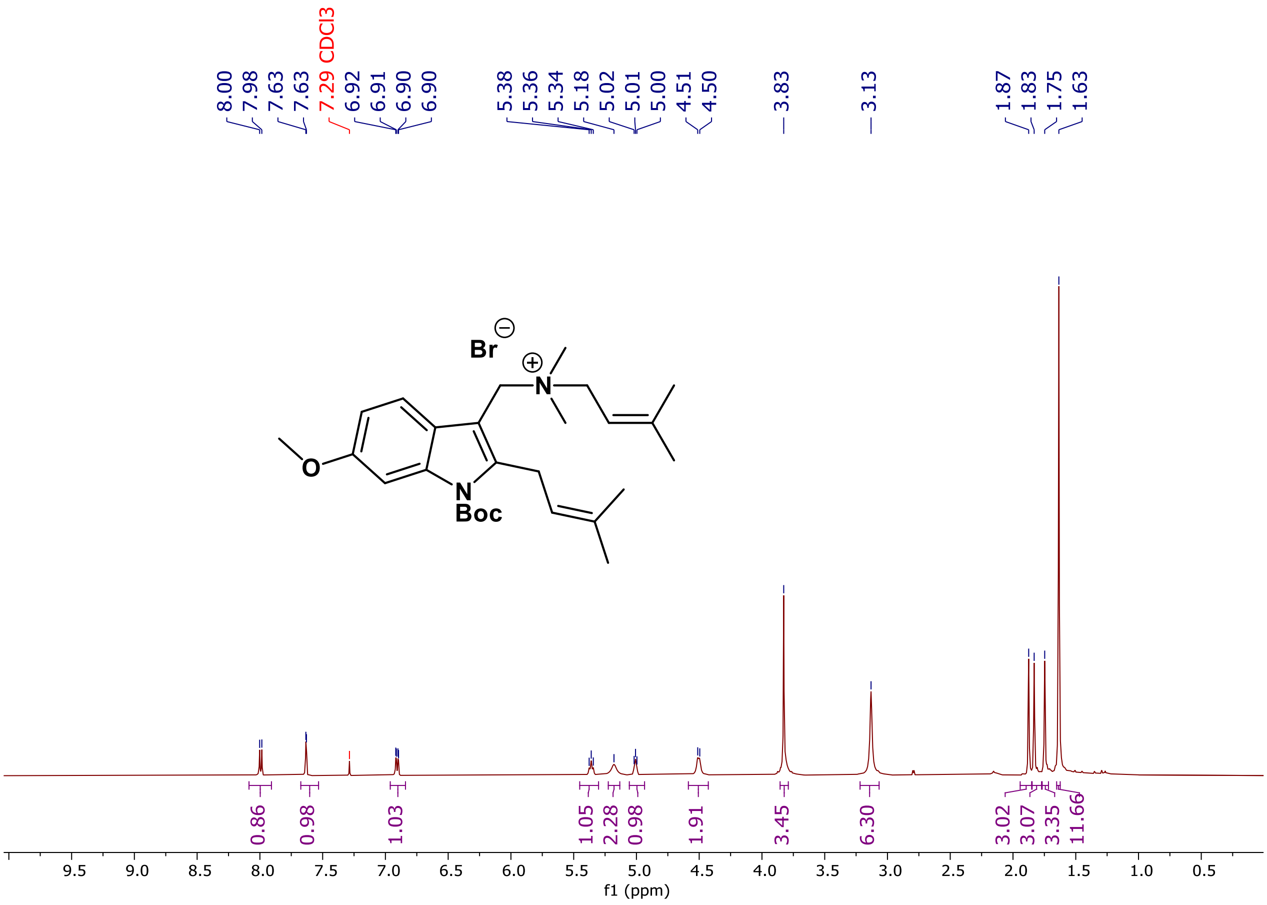

^{13}C NMR of **20** (500 MHz, $CDCl_3$)

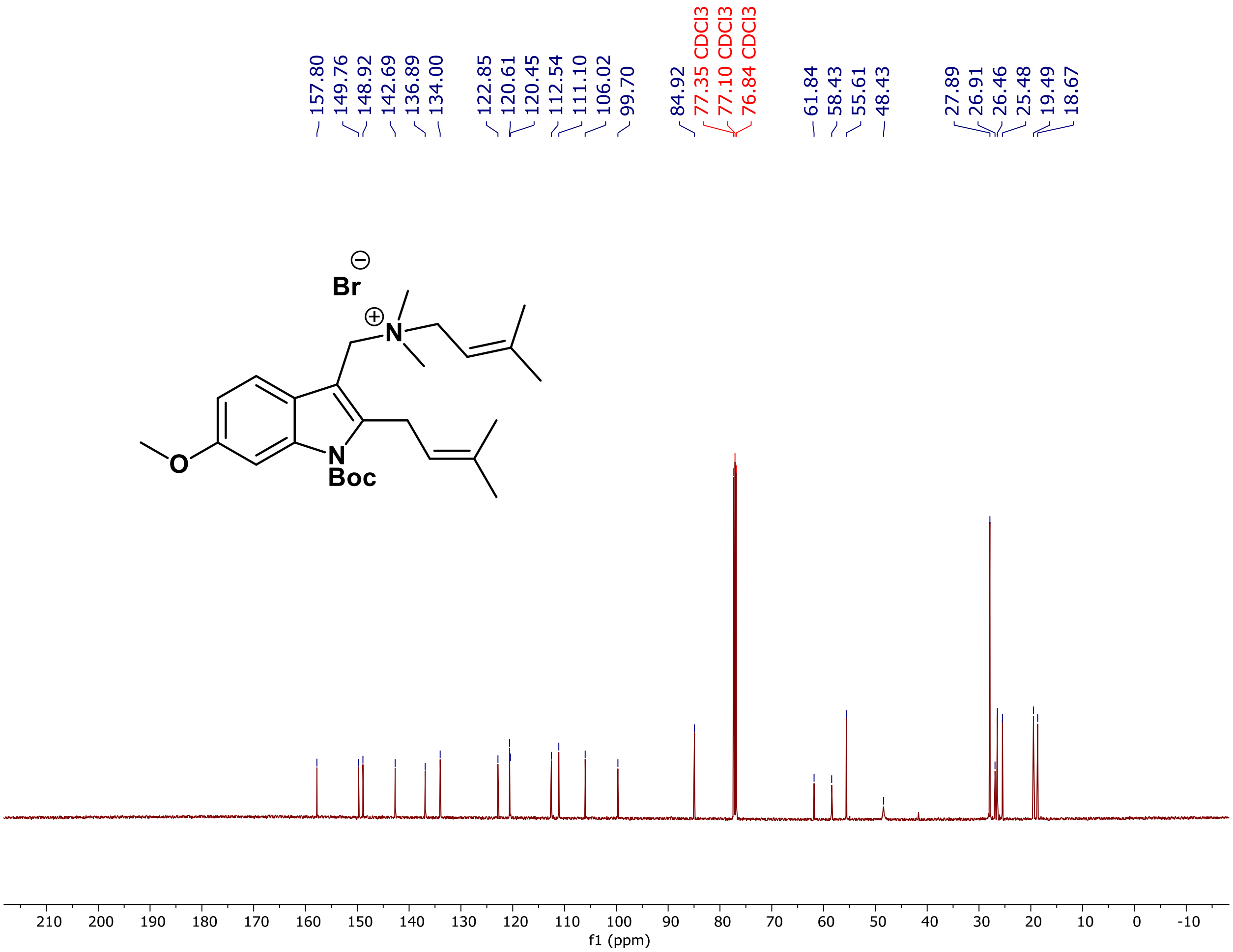

HRMS of **20**

5-23-2025.lcb

Formula Predictor Result	**C27 H41 N2 O3**
Mass	441.31098
Error Margin	100 ppm
DBE Range	Not Used
Electron Ions	Both configurations
HC Ratio	Not Used
Nitrogen Rule	Not Used

Score	Pred. (M)	Pred. m/z	Meas. m/z	Diff. (mDa)	Formulae (M)	Ion	Diff. (ppm)	Iso Score	DBE
71.67	441.31172	441.31117	441.31098	-0.19	C27 H41 N2 O3	[M]+	-0.431	68.71	8.5

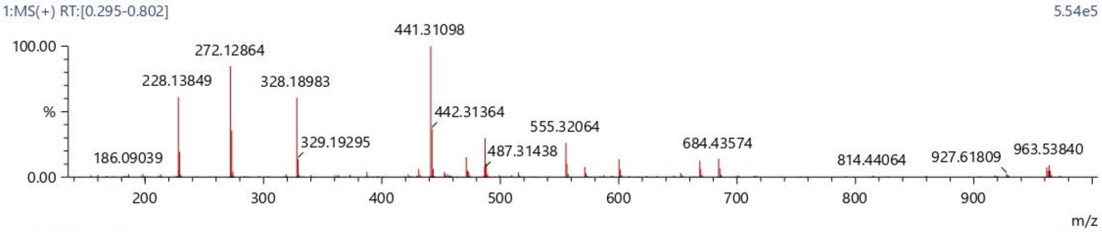

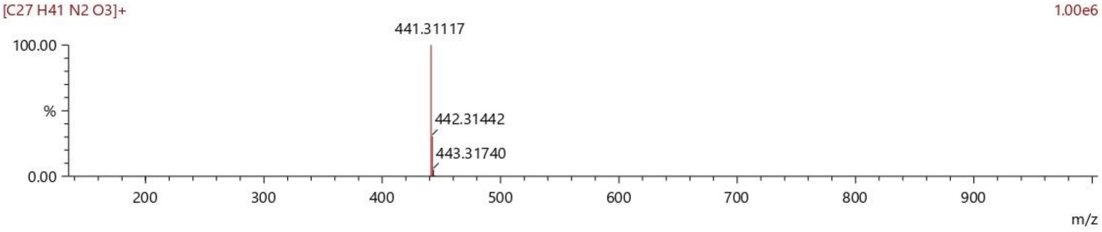

^{1}H NMR of **21** (500 MHz, $CDCl_3$)

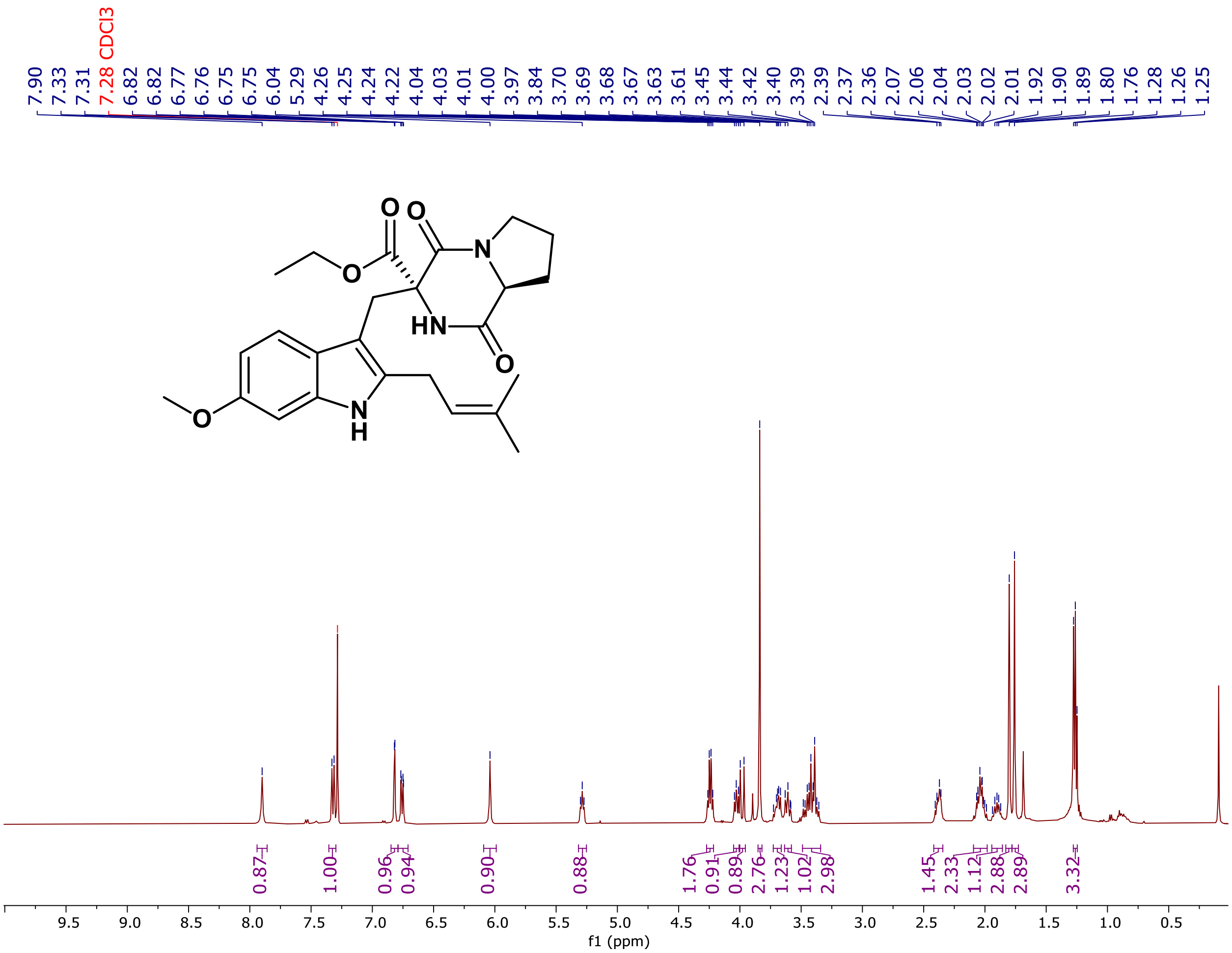

^{13}C NMR of **21** (500 MHz, $CDCl_3$)

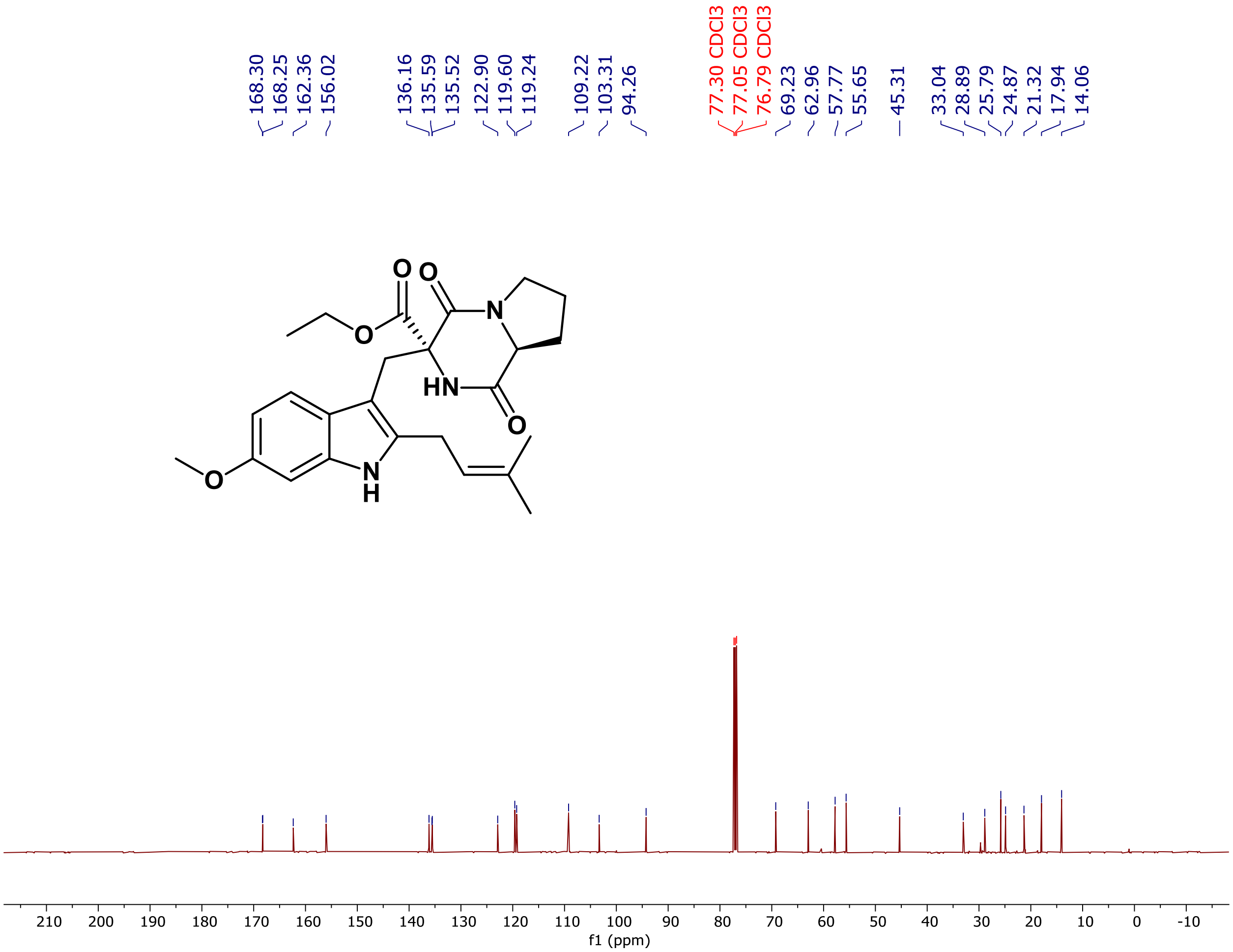

HRMS of **21**

Formula Predictor Report - TPS-A less polar_06042021 Analysis HRMS_36.lcd Page 1 of 1

Data File: C:\LabSolutions\Data\Vilashini Rajaratnam\06042021 Analysis\TPS-A less polar_06042021 Analysis HRMS_36.lcd

Elmt	Val.	Min	Max	Elmt	Val.	Min	Max	Elmt	Val.	Min	Max	Elmt	Val.	Min	Max	Use Adduct
H	1	25	35	O	2	2	6	P	3	0	0	I	3	0	0	H
2H	1	0	0	F	1	0	0	S	2	0	0					Na
C	4	20	30	Na	1	0	0	Cl	1	0	0					K
N	3	2	5	Si	4	0	0	Br	1	0	0					NH4

Error Margin (ppm): 500
HC Ratio: unlimited
Max Isotopes: all
MSn Iso RI (%): 75.00

DBE Range: -100.0 - 2000.0
Apply N Rule: no
Isotope RI (%): 1.00
MSn Logic Mode: AND

Electron Ions: both
Use MSn Info: yes
Isotope Res: 10000
Max Results: 10

Event#: 1 MS(E+) Ret. Time : 0.440 -> 0.787 - 0.147 -> 0.191 Scan# : 67 -> 119 - 23 -> 29

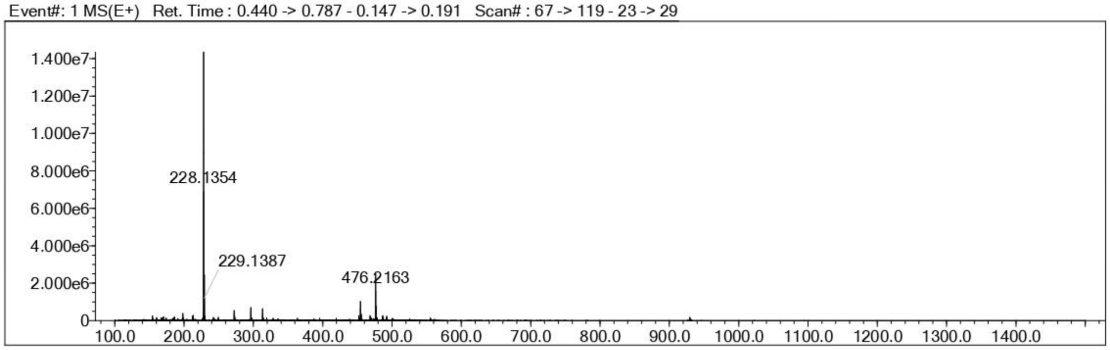

Measured region for 476.2163 m/z

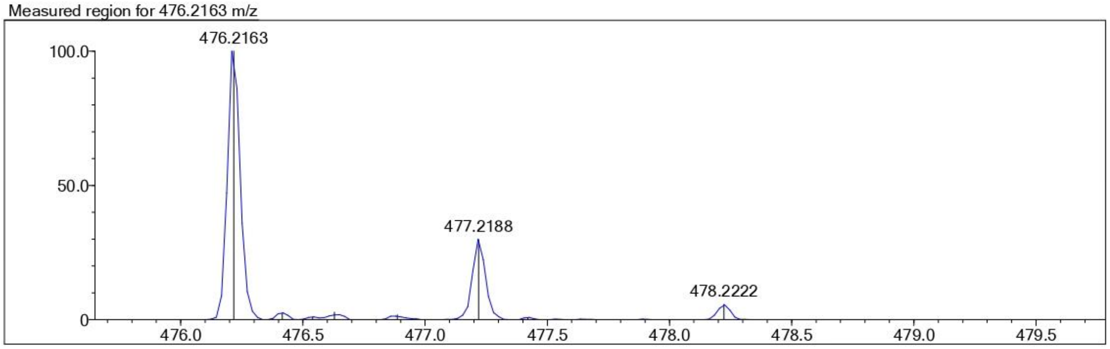

C25 H31 N3 O5 [M+Na]+ : Predicted region for 476.2156 m/z

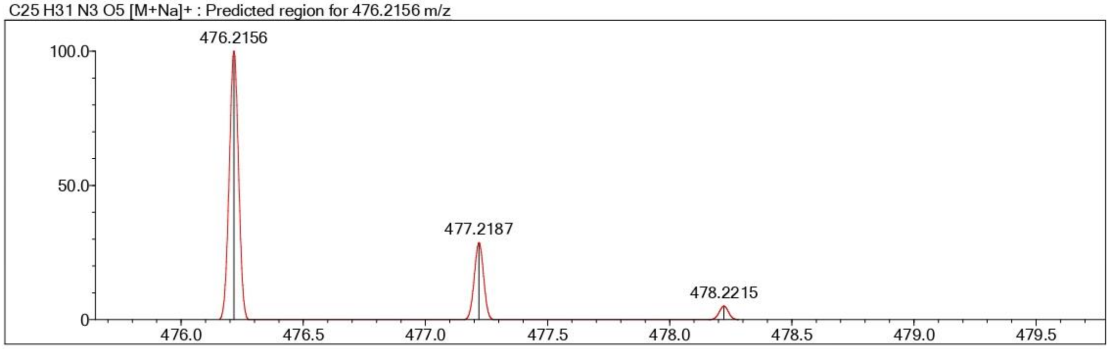

Rank	Score	Formula (M)	Ion	Meas. m/z	Pred. m/z	Df. (mDa)	Df. (ppm)	Iso	DBE
1	94.61	C25 H31 N3 O5	[M+Na]+	476.2163	476.2156	0.7	1.47	95.74	12.0

^{1}H NMR of **22** (500 MHz, $CDCl_3$)

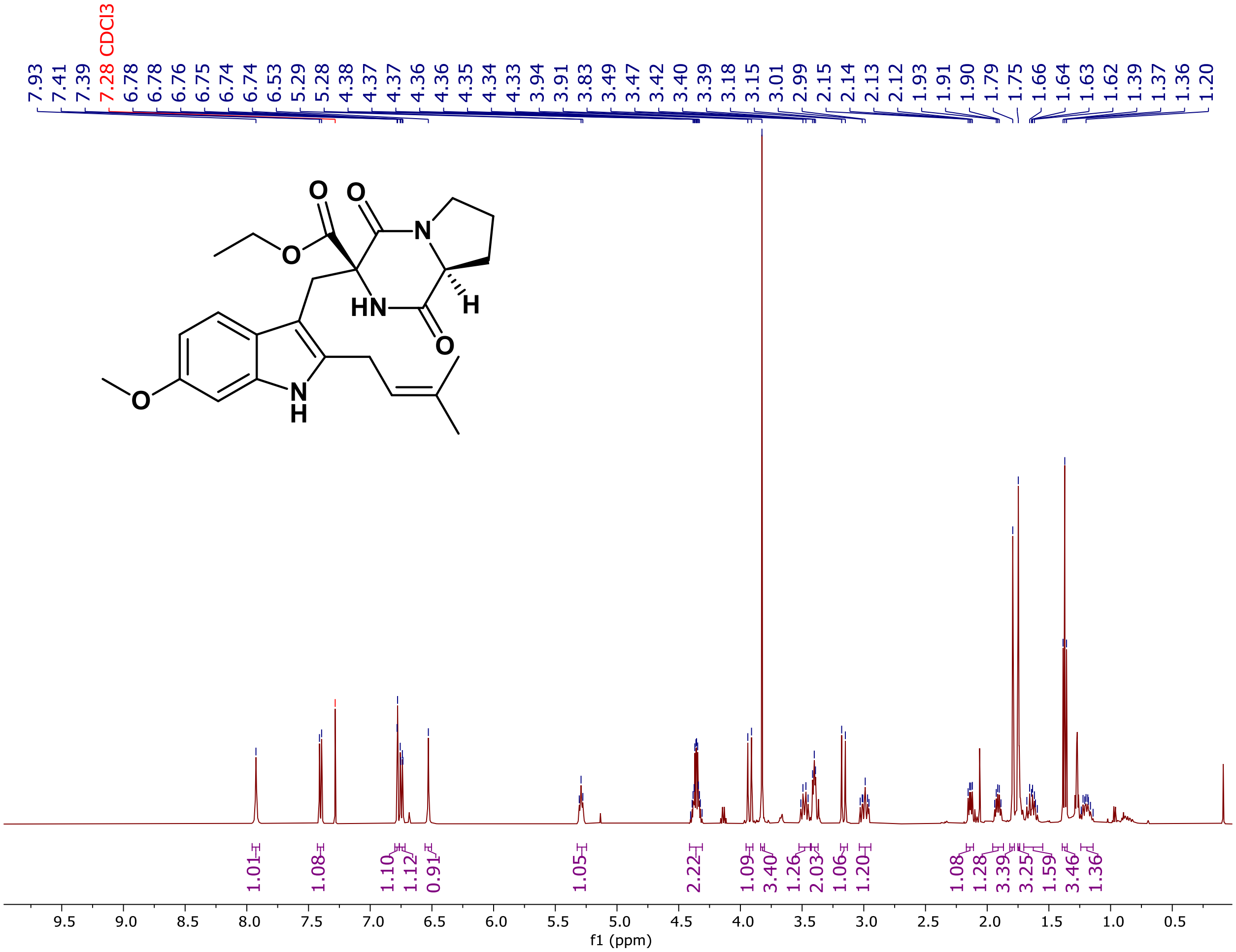

^{13}C NMR of **22** (500 MHz, $CDCl_3$)

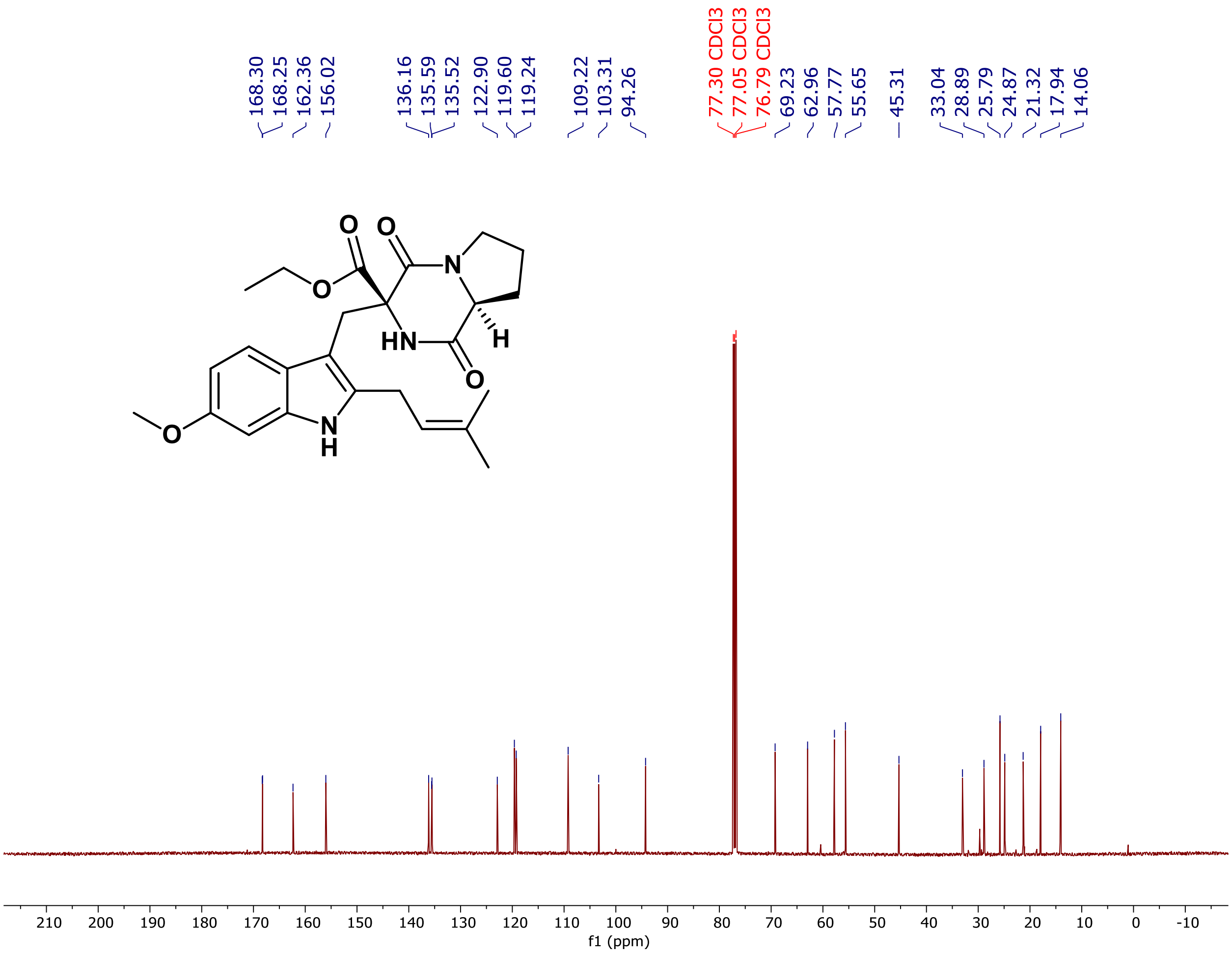

HRMS of **22**

5-23-2025.lcb

Formula Predictor Result	**C25 H31 N3 O5**
Mass	454.2325
Error Margin	100 ppm
DBE Range	Not Used
Electron Ions	Both configurations
HC Ratio	Not Used
Nitrogen Rule	Not Used

Score	Pred. (M)	Pred. m/z	Meas. m/z	Diff. (mDa)	Formulae (M)	Ion	Diff. (ppm)	Iso Score	DBE
84.41	453.22637	454.23365	454.23250	-1.15	C25 H31 N3 O5	[M+H]+	-2.532	84.33	12.0

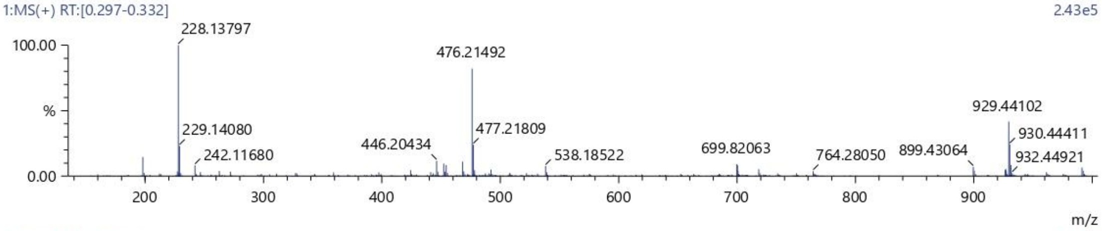

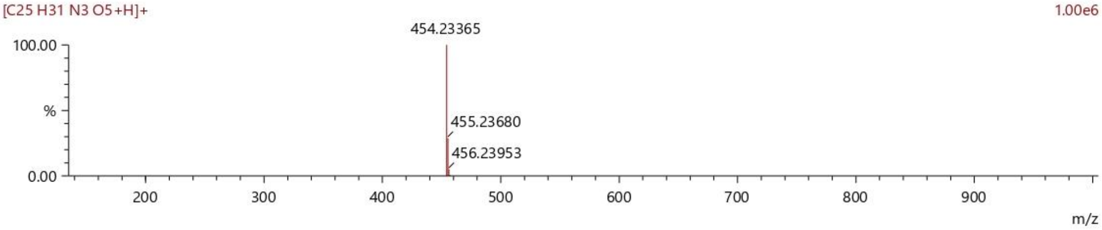

^{1}H NMR of **1** (500 MHz, $CDCl_3$)

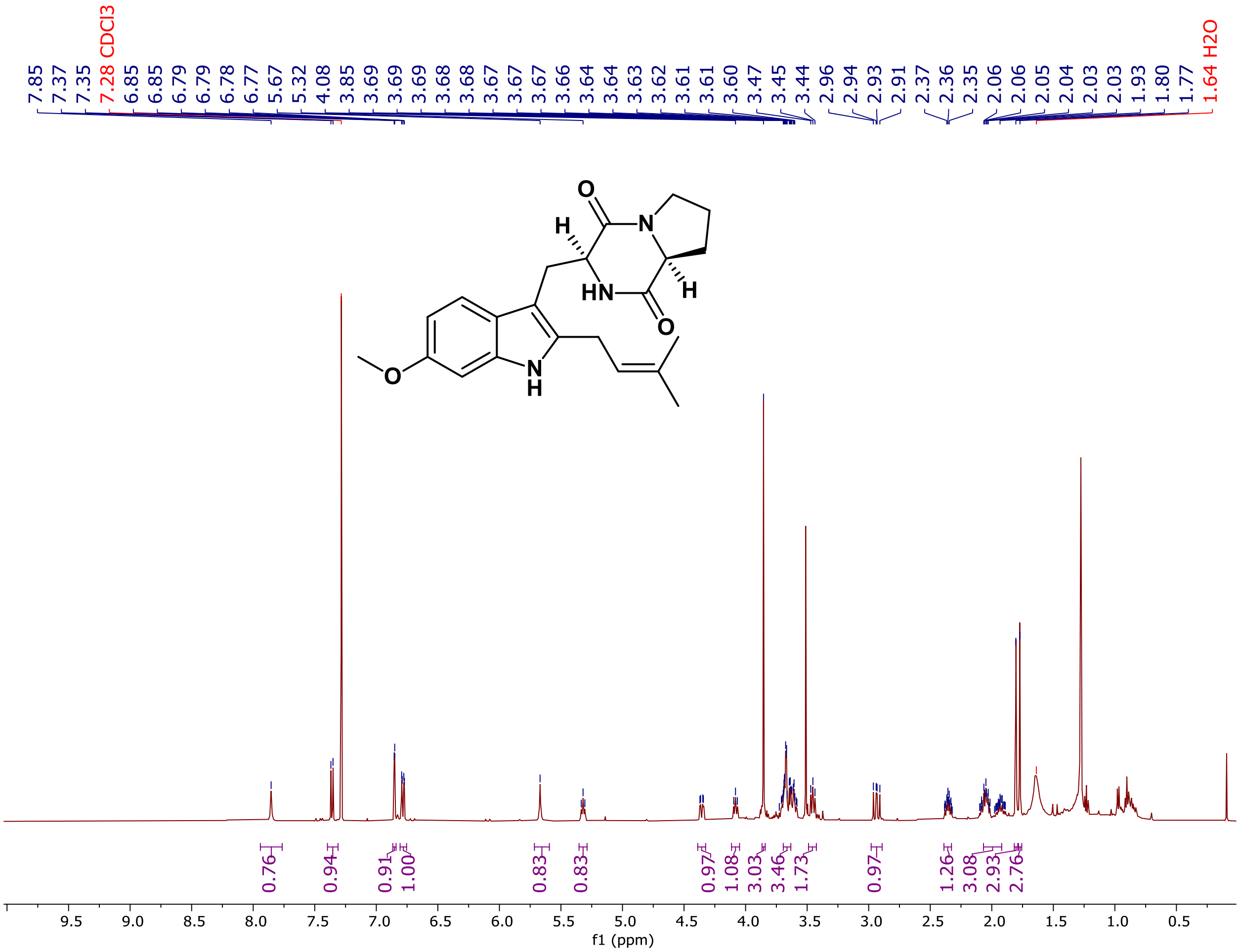

^{13}C NMR of **1** (500 MHz, $CDCl_3$)

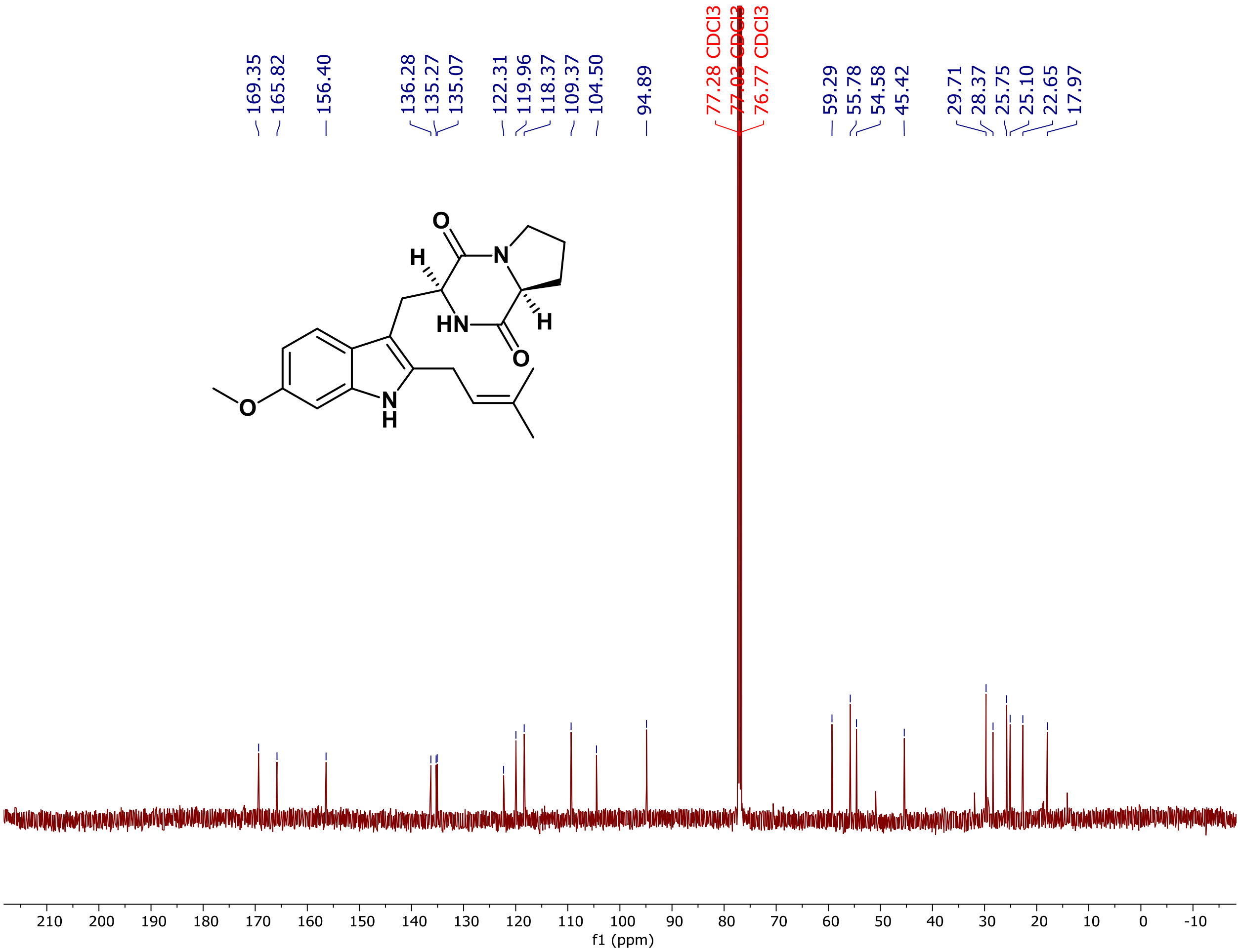

^{1}H NMR of **16** (500 MHz, $CDCl_3$)

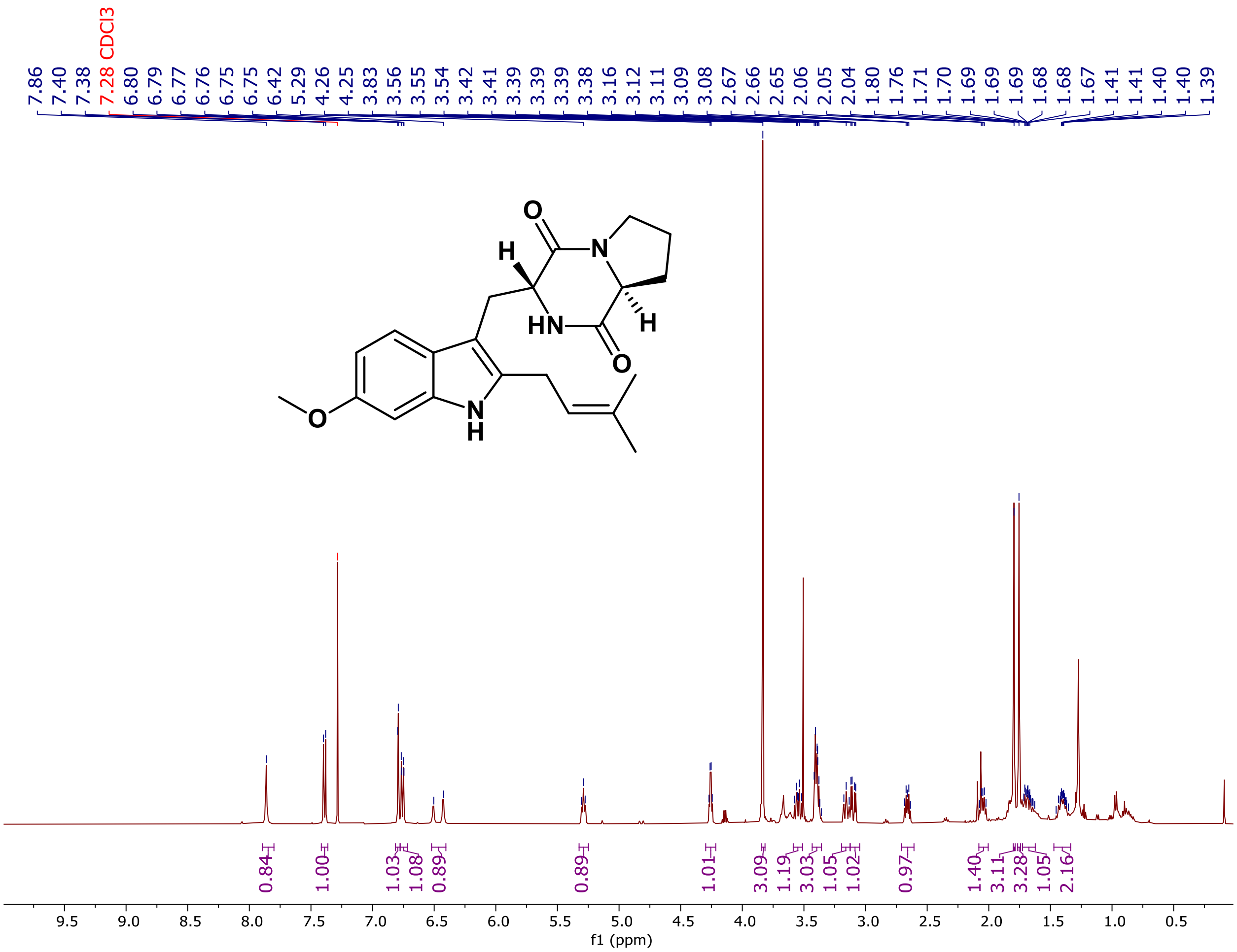

^{13}C NMR of **16** (500 MHz, $CDCl_3$)

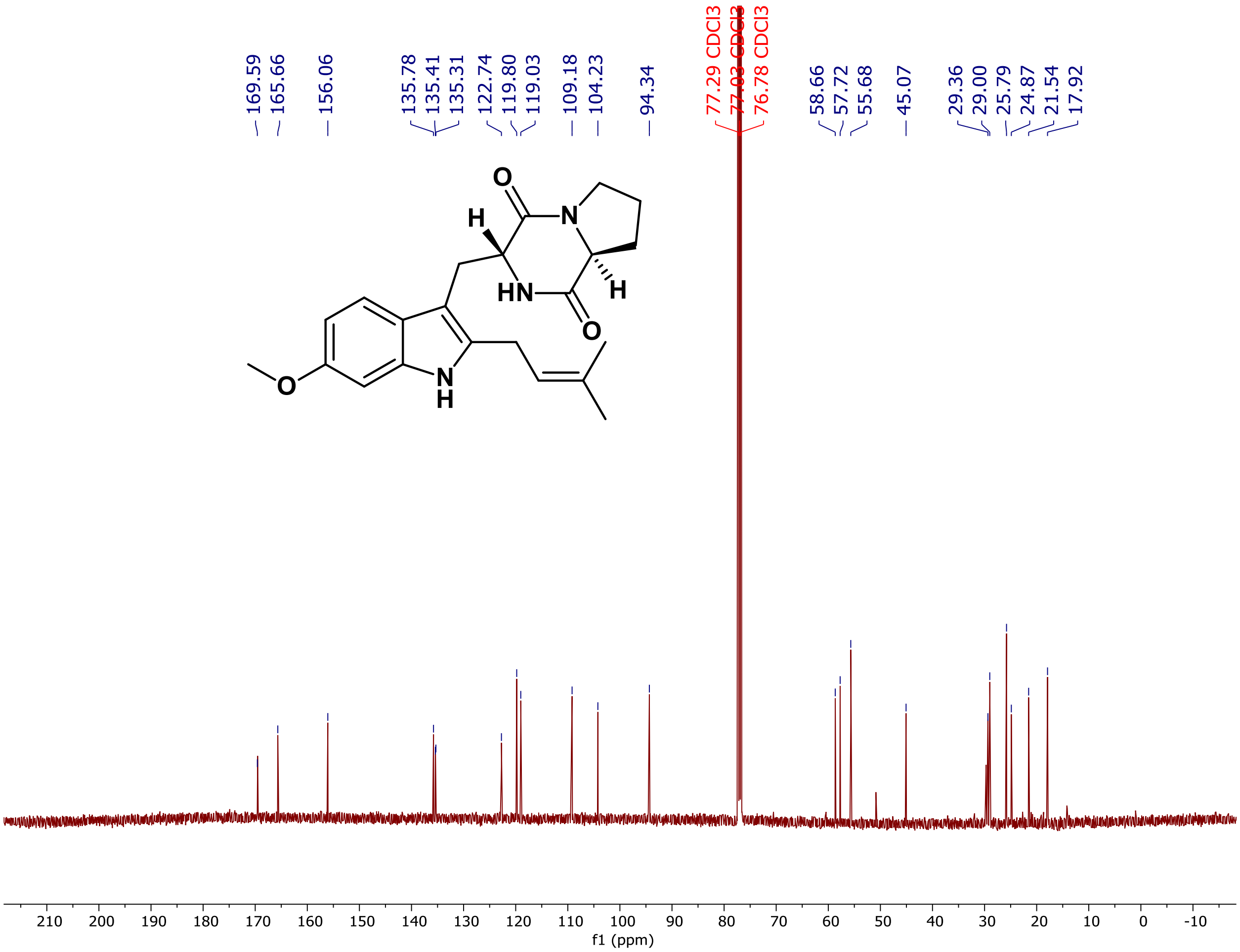

Supplement: Supplementary file 1 [file ao5c07992_si_001.pdf]
